# Tuning the Properties of Furandicarboxylic Acid-Based Polyesters with Copolymerization: A Review

**DOI:** 10.3390/polym12061209

**Published:** 2020-05-26

**Authors:** Zoi Terzopoulou, Lazaros Papadopoulos, Alexandra Zamboulis, Dimitrios G. Papageorgiou, George Z. Papageorgiou, Dimitrios N. Bikiaris

**Affiliations:** 1 Laboratory of Chemistry and Technology of Polymers and Dyes, Department of Chemistry, Aristotle University of Thessaloniki, GR-541 24 Thessaloniki, Greece; terzoe@gmail.com (Z.T.); azampouli@chem.auth.gr (A.Z.); 2 Department of Chemistry, University of Ioannina, P.O. Box 1186, 45110 Ioannina, Greece; lazaros.geo.papadopoulos@gmail.com (L.P.); gzpap@uoi.gr (G.Z.P.); 3 School of Engineering and Materials Science, Queen Mary University of London, Mile End Road, London E1 4NS, UK; d.papageorgiou@qmul.ac.uk

**Keywords:** biobased polymers, furan dicarboxylic acid, polyesters, copolymers, thermal properties, gas barrier properties, mechanical performance, biodegradation

## Abstract

Polyesters based on 2,5-furandicarboxylic acid (FDCA) are a new class of biobased polymers with enormous interest, both from a scientific and industrial perspective. The commercialization of these polymers is imminent as the pressure for a sustainable economy grows, and extensive worldwide research currently takes place on developing cost-competitive, renewable plastics. The most prevalent method for imparting these polymers with new properties is copolymerization, as many studies have been published over the last few years. This present review aims to summarize the trends in the synthesis of FDCA-based copolymers and to investigate the effectiveness of this approach in transforming them to a more versatile class of materials that could potentially be appropriate for a number of high-end and conventional applications.

## 1. Introduction

As the worldwide waste accumulation keeps increasing and the fossil-based energy sources and chemicals are rapidly depleting, governments, industries, and academia have turned their focus on developing methods to optimize the exploitation of natural resources toward a sustainable ‘green’ future. According to the European Commission (EC), bioeconomy is “the production of renewable biological resources and the conversion of these resources and waste streams into value-added products, such as food, feed, bio-based products and bioenergy” [1]. The EC has set a specific strategy on bioeconomy that includes an increase in the funding for research and innovation and scaling-up of the biobased industrial sector [2]. This strategy is reflected on the €3.7 billion public–private partnership, the Bio-Based Industries Joint Undertaking, which operates under Horizon 2020 [3].

Since the 1950s, the use of plastics dominates everyday life, and it is estimated than in 2050, the global cumulative plastic waste generation will exceed 25 billion tons, of which an impressive 12 billion tons will end up either in landfills or in the environment, and only a meager 9 billion tons will be recycled [4]. The effect of petrochemical-based plastics on the environment is multifaceted; it includes the depletion of petrochemical resources, the increase of the atmospheric CO_2_ levels, and the rapid accumulation of waste in both land and oceans. In this light, great efforts have been undertaken to replace conventional plastics with new, sustainable biobased plastics synthesized with monomers derived from biomass. The effective isolation of renewable monomers and the large-scale synthesis of their corresponding plastics is an area where both academia and industry have been focusing on during the last few years. 

One of the most interesting monomers derived from biomass is 2,5-furan dicarboxylic acid (FDCA), which is an oxidation product of furfural that is included in the top value-added chemicals from biomass list as compiled by the US Department of Energy [5]. Its importance arises from its chemical structure, as it contains a rigid furan ring and two di-acidic side chains that can easily yield condensation polymers, similarly to terephthalic acid (TPA). Not surprisingly, many companies are therefore either focused on producing or are planning to produce FDCA from biomass in the near future (e.g., Avantium, Novamont, AVA Biochem, Origin Materials, Corbion) or its dimethylester dimethyl furan dicarboxylate (DMFD) (DuPont) [6]. 

A plethora of polymers can be synthesized starting from FDCA such as polyesters, polyamides, polyurethanes, and thermosets [7]. As a result of its similarity with TPA, FDCA-based polyesters are assumed to be the biobased homologues of highly-popular TPA-polyesters such as poly(ethylene terephthalate) (PET), poly(propylene terephthalate) (PPT), and poly(butylene terephthalate) (PBT). Poly(ethylene 2,5-furan dicarboxylate) (PEF) is the most important polyester derived from FDCA due to its similarity with PET, and it is expected to start being commercialized in 2023 [8] and reach a market value of $129.3 million by 2025 [9]. In contrast with most Europe-based companies that are planning on commercializing PEF, DuPont has turned its focus on poly(propylene 2,5-furan dicarboxylate) (PPF) [10], most likely for fiber applications. In addition to their renewable nature, both polyesters have better mechanical, barrier, and thermal properties than their TPA homologues PET and PPF [11], as reported by a large body of literature [12,13,14,15,16,17,18,19]. The implementation of two big European projects, namely PEFerence (No 744409) funded by Horizon2020 and the COST action FUR4Sustain (CA18220) will boost innovation, aiming to overcome current obstacles and to push toward the commercialization of FDCA-based polyesters. 

Regardless of their great potential as biobased polymers, FDCA-based polyesters have their limitations. A number of them display slow crystallization rates, a lack of biodegradability, and high rigidity and fragility, which can limit their overall use. Numerous researchers have applied the method of copolymerization to modify the properties of FDCA-based polyesters with a variety of cyclic or aliphatic diols and/or diacids. A summary of the copolyesters reported in the open literature is presented in Table 1.

Nowadays, various copolyesters such as poly(butylene adipate-*co*-terephthalate) (PBAd-*co*-PBT), poly(1,4-cyclohexanedimethylene terephthalate-*co*-isophthalate) (PCHDMT-*co*-PCHDMI), poly(ethylene-*co*-1,4-cyclohexanedimethylene terephthalate) (PET-*co*-PCHDMT), poly(ethylene terephthalate-*co*-glycolate) (PET-*co*-PEG), poly(lactide-*co*-glycolide) (PLA-*co*-PGA) find applications in specialty packaging, agriculture, and medicine. The comonomers used provide either improved or new properties depending on the final application. For example, adipate units in PBAd-*co*-PBT copolymers (Ecoflex^®^, Origo-Bi) provide them with biodegradability [20]. Commercial products of copolyesters include Tritan^®^, Glass Polymer^®^, Eastar^®^, Vistel^®^ (thermoplastic resin), Dynacoll^®^ S, Petaflex^TM^, and others. Therefore, it may be of no surprise that copolymerization, as a well-known, widely applied designing method of polymers with tunable properties, is also expected to be applied on FDCA-based polyesters. A small number of patents has already been filed concerning FDCA-based copolyesters with pryomellitic dianhydride, pentaerythritol and their combinations [21], polyethers [22], isosorbide [23] and bis(hydroxymethyl)cyclohexane (cis, trans or both), 2,2-dimethyl-1,3-propanediol (PD), poly(ethylene glycol) (PEG), poly(tetrahydofuran), glycerol, pentaerythritol, lactic acid, 6-hydroxyhexanoic acid [24].

The literature on polymers with furan rings has been initially reviewed in 2009 [25], later on with a focus on polyesters in 2013 [11], in 2016 by our group [26] and more recently only a brief review on the progress of FDCA-based polyesters was published [27]. 

As of 2016, only a few publications on FDCA-based copolyesters were available. Since then, the number of publications and citations grew rapidly, revealing the increased scientific interest on this topic. It is also noteworthy that only a limited number of patents on FDCA-based copolyesters is available up to date [21,22,23,24]. The aim of this review article is to collect and sum up all the information provided by the published literature on FDCA-based copolyesters, with focus on the tuning of the properties depending on the type of comonomers used, as well as their potential applications.

## 2. Synthesis, Molecular Weight, and Randomness of FDCA-Based Copolyesters

As illustrated in Table 1, a wide library of FDCA-based copolyesters has been investigated in the literature. The vast majority of these copolymers were prepared by melt polycondensation reactions at high temperatures. A number of alternative approaches has also been investigated; the synthesis of cyclic furanoate oligomers has allowed for the preparation of furanoate copolymers, notably PBF copolymers, by ring-opening polymerization (ROP) [64,66,83,86,98,105]. Additionally, the use of enzymatic polymerization has been employed, which warrants a more sustainable synthetic procedure and milder reaction conditions [80,86,93].

### 2.1. Melt Polycondensation

**Scheme 1:**
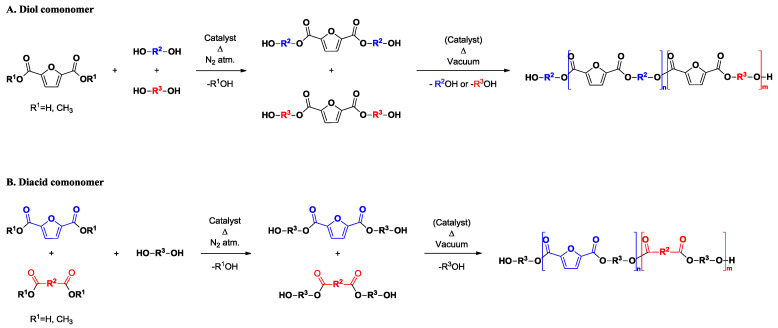
General scheme for the synthesis of copolymers by two-stage melt polycondensation.

Two-step melt polycondensation typically involves an initial esterification or transesterification step, followed by a polycondensation step, generally conducted at higher temperatures and under reduced pressure, as illustrated in Scheme 1. DMFD is the preferred starting monomer, due to its easier purification and higher thermal stability compared to FDCA; however, many research groups have used FDCA as well. Typical procedures include heating below 200 °C for the first step and over 200 °C for the second one. The duration varies between 1 and 7 h for each step. The end of the first step is often set when 90–95% of the theoretically produced methanol or water has been collected. The end of polycondensation is indicated by a constant torque value of the mechanical stirrer or by a net appearance of the so-called Weissenberg effect. Sometimes, solid-state polymerization is also performed in order to further increase the molecular weight of the copolymers.

The most popular catalysts used for the melt polycondensation procedure are titanium(IV) butoxide (TBT) and antimony(III) oxide (Sb_2_O_3_). Titanium(IV) isopropoxide is regularly used, solely or in combination with TBT [28,29,60,68,77,93,97,100]. Zinc(II) acetate is also often used for the first step (Sb_2_O_3_ is generally added for the polycondensation step) [30,31,34,35,36,48,58,59,102]. Dubois et al. have synthesized a home-made titanium-silica complex that exhibited high catalytic activity [37,45,56]. Lanthanum(III) acetylacetonate [73,79], polyphosphoric acid [72], cobalt acetate [41], dibutyltin oxide [33,68], and EG antimony [48] have also occasionally been used. Additives, mainly Irganox 1010, a sterically hindered phenolic primary radical scavenger common in the synthesis of many types of polymers, are periodically used as antioxidants and heat stabilizers [48,56,58,85,91].

When copolymerization aims at the introduction of lactyl or *ε*-oxycaproate units, two different strategies have been adopted. On the one hand, the previously mentioned two-step polymerization process is adapted, and an ROP stage is introduced [49,67,87,95,98]. In other words, esterification or transesterification yields furanoate oligomers; then, *ε*-caprolactone or lactide and a catalyst are added, and ROP takes place. Finally, vacuum is applied, and the temperature is increased for the polycondensation step. On the other hand, polylactide (PLA) or poly(ε-caprolactone) (PCL) oligomers can be prepared separately and added directly in a two-stage polymerization reaction [35,62,85].

The catalyst TBT is the most popular for the ROP step. Otherwise, as previously stated, TBT or Sb_2_O_3_ are used for the two other steps. A noteworthy exception is the use of a metal-free catalyst, 1,5,7-triazabicyclo[4.4.0]dec-5-ene, for the ROP of lactide by PHF oligomers, which was conducted at room temperature for 5 min [98]. Very high average molecular weights were not achieved, but lower ones have also been reported. 

Generally, the composition of the copolymers is consistent with the feed ratio of monomers. Differences arise when the monomers exhibit important reactivity or volatility differences. For example, PEF copolymers with 1,4-butanediol (BDO) [29], 1,5-pentanediol (PeDO) [37], and 1,6-hexanediol (HDO) [45] systematically incorporate a lower content of ethylene furanoate (EF) units, which is attributable to the higher volatility or lower reactivity of EG compared to the three other diols. This is also striking when cyclic diols such as 1,4-cyclohexanedimethanol (CHDM) [32,65] or 2,2,4,4-tetramethyl-1,3-cyclobutanediol (CBDO) [34,35] are used. These diols have higher boiling points than linear diols and thus are often incorporated in higher ratios. Isosorbide on the other hand tends to be incorporated in slightly lower percentages compared to the feed ratios due to the lower reactivity of the secondary hydroxyl group [67].

### 2.2. Ring-Opening Polymerization (ROP)

When it comes to the structure of the products, random copolymers are mostly obtained. Block or multiblock copolymers are obtained in copolymers incorporating polyether segments, such as poly(ethylene glycol) (PEG) or poly(tetramethylene glycol) (PTMG) [46,47,48,56,88,89,90,91,93,94,101]. When ROP with CL or lactide is included in the polymerization, the outcome depends on the experimental conditions, and both random [49,54,85] and block copolymers [54,87,95,98] have been obtained. The structure depends mainly on whether monomers or oligomers are employed as starting products and on whether the conditions are harsh enough to cause some depolymerization of PLA or PCL oligomers, or favor transesterification reactions. A number of works have reported the formation of diethylene glycol units, which are attributed to etherification side-reactions [28,37,38,41,45,49,56].

**Scheme 2:**
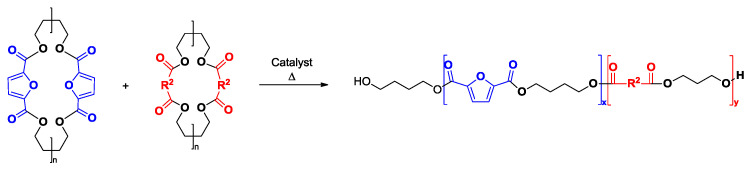
General synthesis of copolymers with PBF by ring-opening polymerization (ROP).

The research group of Dr. Muñoz-Guerra has synthesized various furanoate-containing cyclic oligomers (mixtures of dimers, trimers, and tetramers mainly) and copolymerized them by entropically-driven ROP (Scheme 2) [64,66,69,83,86,105]. One of the advantages of ROP is the high polymerization rate, which results in relatively milder conditions and shorter reaction times compared to traditional two-step melt polycondensations. ROP is actually the preferred polymerization strategy for lactide and caprolactone (CL). Morales-Huerta et al. employed aliphatic as well as aromatic comonomers to prepare FDCA copolymers with stannous octoate (Sn(oct)_2_) as the preferred catalyst. Temperature conditions were optimized, and the temperatures leading to higher reaction rates were between 200 and 220 °C, with lower ones leading to lower reaction rates. PBF-*co*-PBT copolymers were synthesized both by ROP and two-step melt polycondensation, and much higher weight-average molecular weights were obtained by ROP (70,000 g/mol for ROP versus 40000 g/mol for two-step melt polycondensation) [64]. In general, the obtained molecular weight (Mw) ranged between 5000 and 75,000 g/mol, depending on the comonomers. *Candida arctica* lipase B (CALB) was also studied for ROPs, specifically the ROP of cyclo(butylene furanoate) with cyclo(butylene succinate) or CL [83,86]. Lower temperatures (up to 150 °C) and longer reaction times were used. In the case of cyclo(butylene succinate) where chemical and enzymic catalysis were compared, it is noteworthy that the Mw increased with increasing butylene furanoate content when Sn(oct)_2_ was used, while the opposite trend, i.e., increasing Mw with increasing butylene succinate content, was observed when CALB was used. The Mw could be higher (high butylene succinate content) or lower (higher butylene furanoate ratio) for CALB-catalyzed ROPs depending on the monomer feed ratio [83]. Apart from one instance, where some cyclo(butylene succinate) loss was noted, the composition of the copolyesters was in good correlation with the monomer feed ratio, with some slight deviations. The structure of the obtained copolymers was mostly random, indicating that extensive transesterification reactions take place concomitantly with ROP. Copolymers with CL and isomannide units tended to exhibit a more blocky structure, depending on the feed ratio [66]. 

### 2.3. Enzymatic Synthesis

Enzymatically catalyzed polymerizations are interesting alternatives to polycondensation reactions, as they generally require milder conditions and exhibit higher selectivity. As a result, apart from being less energy-intensive and more sustainable, fewer side reactions occur. In the case of FDCA polymers, this is particularly interesting, as milder conditions would prevent decarboxylation reactions that result in undesired low Mw products compared with the traditional melt polycondensation method. This was clearly shown in the case of PBF-*co*-PBS copolymers, where the ones synthesized via melt polycondensation at temperatures up to 250 °C had M_n_ between 15 and 40 g/mol [79], while copolymers of the same composition synthesized at temperatures between 120 and 150 °C with enzymes had M_n_ between 21 and 50 g/mol [83]. Additionally, the thermal stability of polymers prepared with either method was similar. On the downside, if a substrate is not suitable for an enzyme, low yields are observed. In all, the enzymatic synthesis of polyesters is characterized by mild conditions compared to melt polycondensation and although a small number of studies is available at the moment to directly compare the properties of copolymers prepared with both techniques, it has been shown that high molecular weight polyesters with good thermal stability can be prepared using both methods. However, not every substrate can undergo enzymatic polymerization and the distinct activity of the enzyme toward different building blocks is observed [80]. Moreover, the industrialization of this method is not considered viable at the moment, as economic limitations are still present. 

**Scheme 3:**
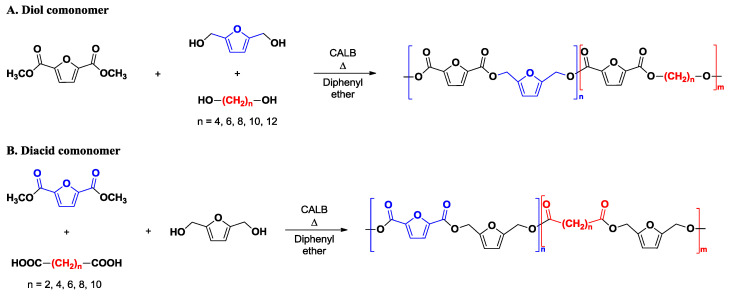
Enzymatic synthesis of 2,5-furandicarboxylate and 2,5-bis(hydroxymethyl)furan copolymers, (**A**) diol comonomer, and (**B**) diacid comonomer.

As mentioned previously, enzymically catalyzed ROP was successfully investigated by Morales-Huerta et al. [86]. Enzymically catalyzed polycondensations have been reported by Todea et al. [106] and Maniar et al. [80]. Maniar et al. prepared copolymers from DMFD, 2,5-bis(hydroxymethyl)furan, and aliphatic linear diols (Scheme 3a) or the corresponding diacid ethyl esters (Scheme 3b), having 4–12 carbon atoms [80]. Polymerizations were conducted in diphenyl ether for 2 h at 80 °C under atmospheric pressure and then under vacuum, for 48 h at 80 °C and 24 h at 95 °C. The average degree of polymerization ranged from 5 to 120 (number average) and 7 to 270 (weight average). Diols were generally better substrates compared to the corresponding acids, i.e., higher polymerization degrees were obtained, and longer diols were preferred to shorter ones (the highest degree of polymerization was obtained with 1,8-octanediol). These differences were attributed to the substrate specificity of *Candida arctica* lipase B (CALB). Additionally, the polymerization degree dropped significantly when the aromatic content increased. Todea et al. copolymerized 5-hydroxymethyl-2-furoic acid with CL [106]. Three different immobilized CALB enzymes were investigated. Temperatures ranged between 40 and 80 °C, with polymerization being favored by higher temperatures. Linear and cyclic adducts were obtained, but the polymerization degrees were low (10–25).

### 2.4. Reactive Blending

Reactive blending is another strategy to prepare copolymers with tailored properties. Reactive blending consists of heating polymer blends above the melting temperatures of their components, inducing chain scissions and transesterification reactions, resulting ultimately in the formation of copolymers. Guidotti et al. introduced 1,4-cyclohexane subunits in PPF polymers by reactive blending of the corresponding homopolymers [61]. From the ^1^H NMR spectra, the degree of randomness (the probability of finding 1,4 cyclohexane units next to furan units) was found to be proportional to the reaction time, i.e., the block length decreased as the reaction proceeded. Poulopoulou et al. simulated reactive blending in DSC pans by heating blends at 20 °C/min and quenching them at −30 °C [107,108]. PEF-*co*-PPT, PPF-*co*-PPT, and PEF-*co*-PBF random copolymers were prepared this way. A decrease in IV values was observed compared to the parent homopolymers.

In conclusion, interesting, more sustainable, synthetic strategies have been developed as alternatives to the widely used two-step polycondensation polymerizations. The results are encouraging, but for the time being, these alternatives have a narrow scope. CALB is a widely used enzyme due to its temperature and solvent tolerance; however, its substrate specificity might be limiting. ROP polymerizations are very interesting, provided that suitable cyclic oligomers can be synthesized. An instance of a carbodiimide-catalyzed esterification has also been reported, but low Mw were obtained [103]. On the other hand, the high temperatures used for two-step polycondensations tend to cause degradation and side reactions in FDCA copolymers, which are less thermally stable than the terephthalate counterparts, resulting in discolored polymers. Additionally, they are energy-demanding, which may be a drawback for large-scale applications. The development of new, more active, or metal-free catalysts should not be neglected in the search for novel copolyesters with better properties.

## 3. Thermal Properties

### 3.1. Glass Transition (T_g_)

**Figure 1:**
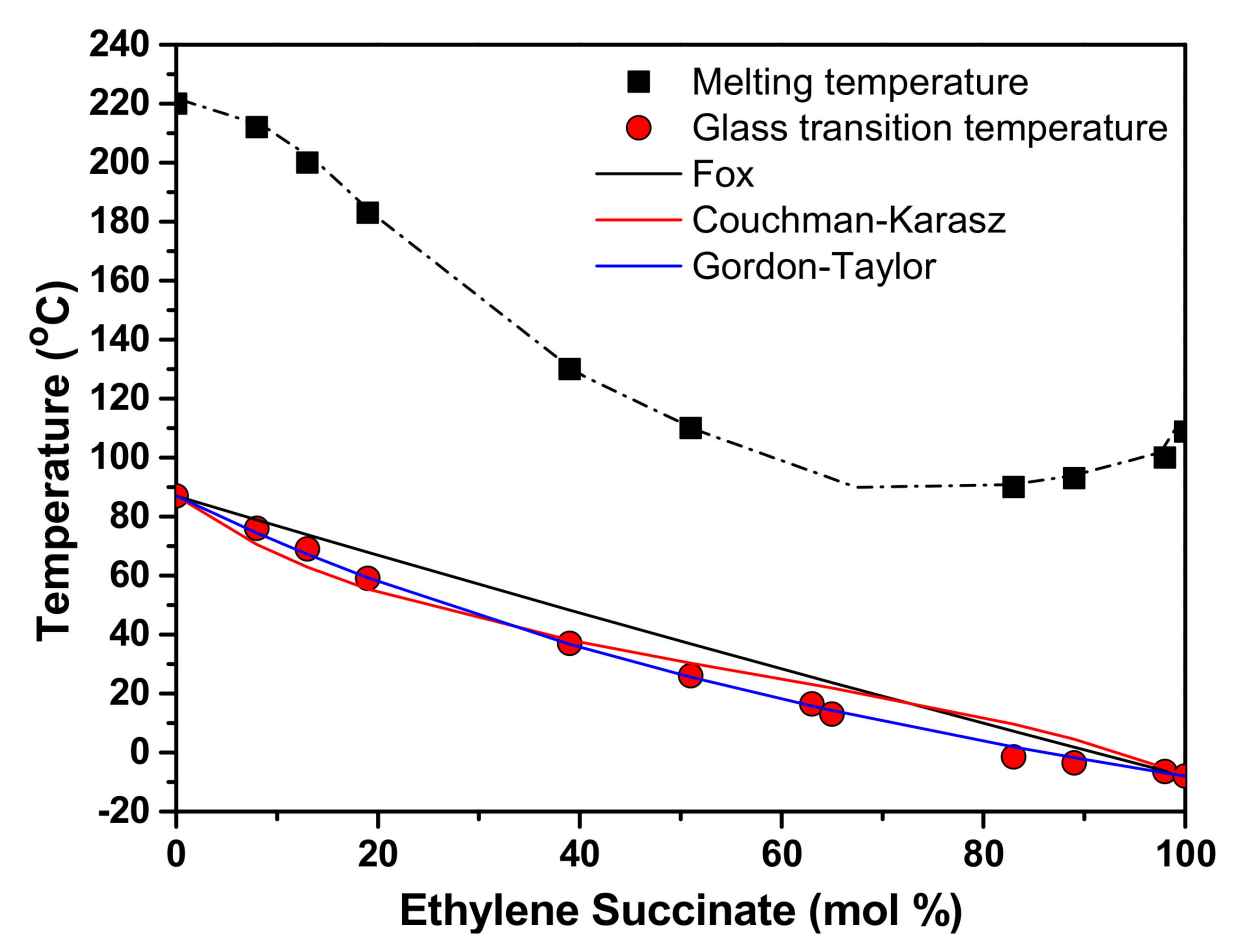
Alterations of the thermal characteristics of the materials depending on the comonomer ratio in PEF-*co*-PES copolymers [84].

One of the most important parameters that determines the possible applications of a material is the *T*_g_. The copolymerization of furan polyesters with various monomers is a tool employed extensively to tune properties of value, and the *T*_g_ is no exception. Generally, when copolymers are synthesized, the *T*_g_ of the materials can be found at intermediate temperatures between the respective T_g_ of the two homopolymers. Especially for amorphous random copolymers, a monotonic change in the *T*_g_ that depends on the composition can be observed [50,52,100]. This phenomenon has been described by several mathematical models that take different parameters under consideration. Among the studies referenced in this review, the most employed model [28,45,50,65,68,70,73,77,78,79,84,97,99,100] is the Fox equation [109]. However, it does not always fit adequately the experimental values, since it is a rather simplified mathematical model that only considers the *T*_g_ of the pristine materials and the composition of the copolymer. There are other models that take additional parameters into account such as the heat capacity associated with the glass transition that can be applied for a more accurate modeling, namely the Gordon–Taylor [110], the Couchman–Karasz [111], and the Kwei [112] equations. An example is presented in Figure 1, where the *T*_g_ of PEF-*co*-PES copolymers is best described with the Gordon–Taylor equation [84]. A combination of these models should be employed by researchers when applicable, since valuable information regarding the *T*_g_ and the randomness of the polymers’ structure can be extracted. 

**Figure 2:**
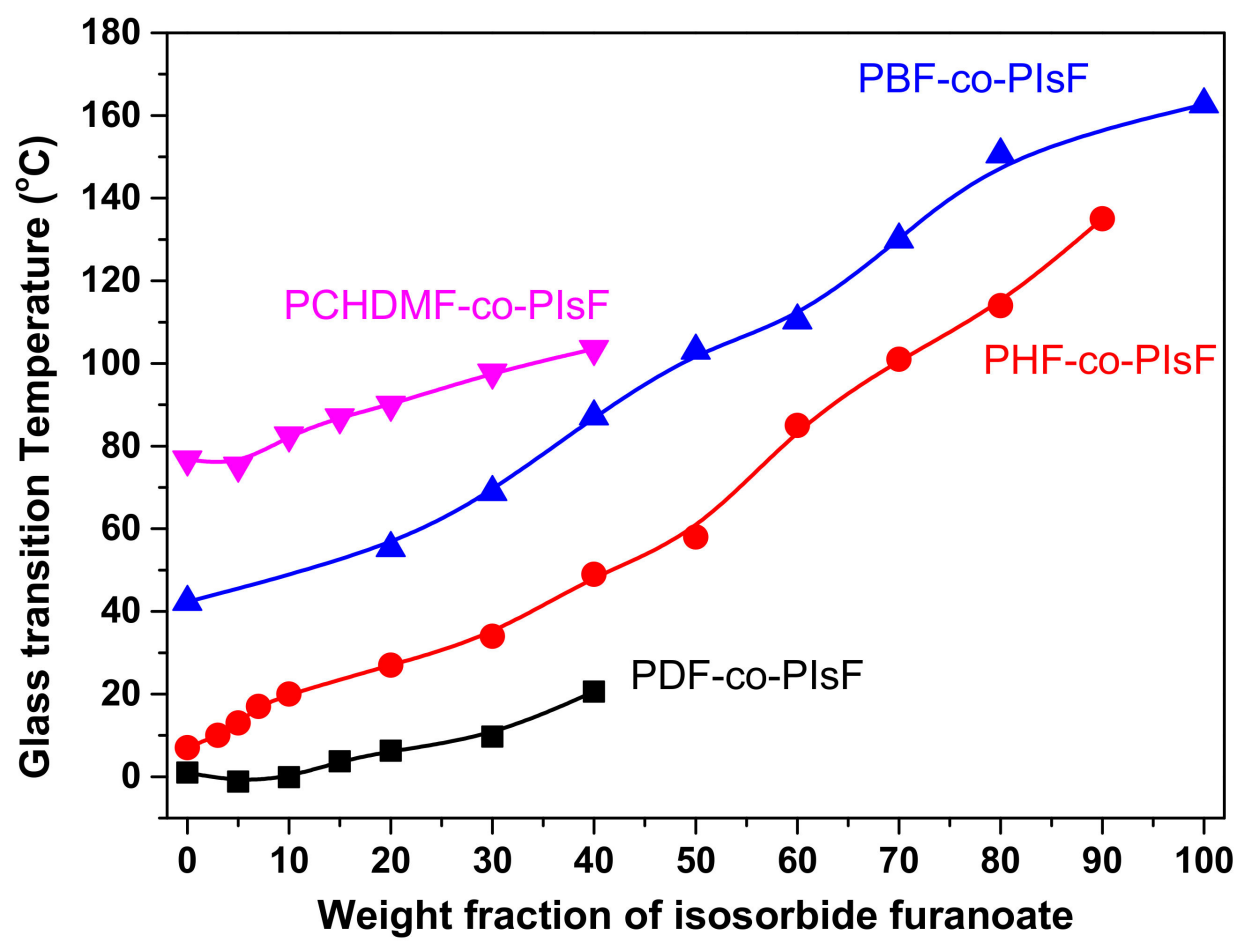
Effect of isosorbide furanoate content on the *T*_g_ of furan-based copolyesters. Data adopted from bibliography [68,97,99,100]. The lines are to guide the eye.

From a synthetic approach, 2,5-FDCA is an excellent monomer to utilize when aiming for materials with high *T*_g_. In all studies described above where two acids and one diol were used for the synthesis of the copolymers, incorporating 2,5-FDCA in the polymer’s structure results in higher *T*_g_ compared to the respective homopolymer, whether derived from an acid with cyclic moieties such as 2,4-FDCA, TPA, and cyclohexane dicarboxylic acid or a linear one such as succinic, adipic, sebacic, etc. The only instance where the *T*_g_ was reduced was in a study by Kainulainen et al. [63], where the bifuran diester was used as a comonomer, and it was due to the reduced mobility of the bifuran ring compared to the furan ring. On the other hand, when two diols are used to prepare copolymers from 2,5-FDCA, two main strategies can be singled out. The use of cyclic diols such as isosorbide [68,97,99,100], CHDM [59,65], and CBDO [34] leads to increased *T*_g_, since the chain mobility of the end products is severely hindered. At the same time, increasing the length of aliphatic diols used always leads to higher chain mobility and thus to lower *T*_g_ values. These results are clearly depicted in Figure 2, where the *T*_g_ variation of PIsF copolymers with various aliphatic diols is shown.

### 3.2. Melting and Crystallization

The crystallization of FCDA-based copolyesters is of utmost importance, as it determines how these materials will be processed, their crystallinity, and ultimately their properties. The tuning of the thermal (and the overall) properties of the copolymers can be performed by adjusting the comonomer ratio in the feed. As a result, a number of studies has focused on the development of random and block copolymers based on 2,5-FCDA and their subsequent crystallization and melting characteristics. 

#### 3.2.1. Block Copolymers 

**Figure 3:**
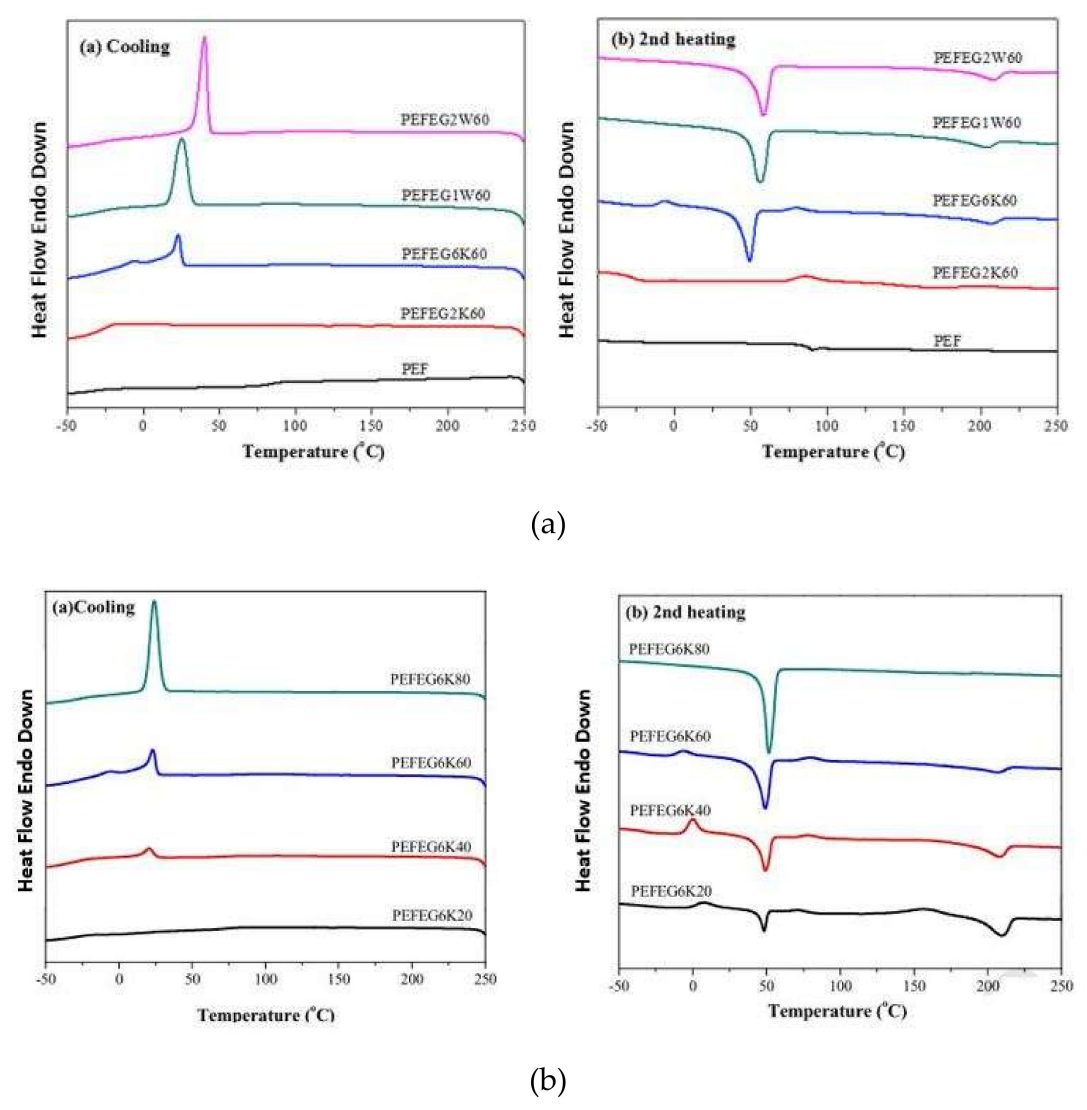
Thermal transitions of PEF-*co*-PEG copolymers: (**a**) fixed ester:ether ratio 40:60, increasing the molecular weight of PEG from 2 K to 20 K, (**b**) fixed molecular weight of PEG at 6 K, increasing the ester:ether ratio from 80:20 to 20:80 [47].

The high stiffness of the furan ring can benefit the formation of 2,5-FDCA block copolymers, where the furan-based polyesters act as the hard segment and can be considered as physical crosslinking points. A number of polymers were used in the literature as a soft segment for the creation of 2,5-FDCA-based copolymers such as PEG [47,48,88,89], poly(tetramethylene glycol) (PTMG) [56,94], PPO [93], and PLA [98]. The thermal behavior of these materials is quite similar and heavily dependent on the block length, as well as to the content of each block. The rigid, aromatic furan segments act as nuclei and are responsible for crystallization while the linear, soft segments are associated with the amorphous region of the materials, and thus with the *T*_g_. The effect of the molecular weight of PEG and the ether–ester ratio of PEF-*co*-PEG copolymers is presented in Figure 3.

**Figure 4:**
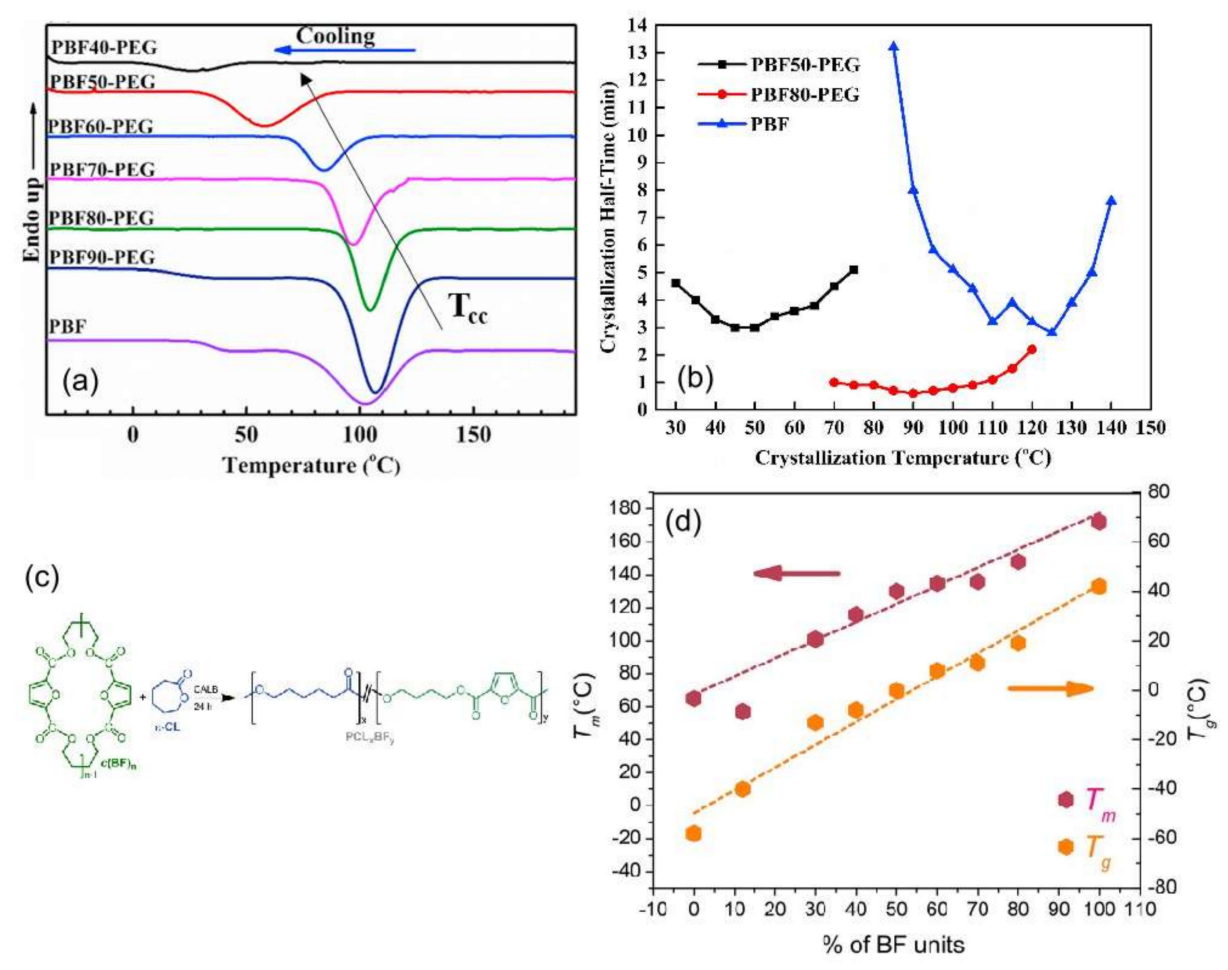
(**a**) DSC cooling scans of PBF-*co*-PEG block copolymers, (**b**) crystallization half-time for PBF50-PEG, PBF80-PEG copolymers, and neat PBF [89], (**c**) e-ROP reaction leading to PBF-*co*-PCL copolyesters and (**d**) *T*_m_ and *T*_g_ of PBF-*co*-PCL against the content of the polymer in butylene furanoate units [86].

As mentioned above, PEG is the most common polymer to be copolymerized with furan-based polymers, and the range of Mw of the PEGs that have been employed ranges from 600 to 20,000 g/mol [47,48,88,89]. As in the case of random copolymers, the presence of the rigid furan ring inhibits or disrupts severely the crystallization of the PEG block. The work of Wang et al. [47] has shown that when increasing the PEG chain length and content, the materials are able to crystallize more easily, while the enthalpy of melting of the PEG block and of the crystallization of the copolymers also increases significantly. The same conclusion has been exported in the case of Hu et al. [89], where PEG essentially acted as a plasticizer for PBF, increased the crystallinity, improved the chain mobility of the copolymers, and eventually assisted in the reorganization, packing, and crystallization (Figure 4a). For the case of isothermal crystallization, the introduction of PEG enhanced the crystallization rates significantly as expected, as can be realized from the graph of crystallization half-times (Figure 4b).

Another interesting work from the group of Munoz-Guerra reported the preparation of blocky PBF-*co*-PCL by copolymerizing cyclic oligo(butylene 2,5-furandicarboxylate) and CL via enzymatic ROP (Figure 4c) [86]. The block copolyesters displayed a semi-crystalline structure, while it is worth noticing that the *T*_g_ and *T*_m_ of the PCL increased significantly with the introduction of furanoate units (Figure 4d). 

**Figure 5:**
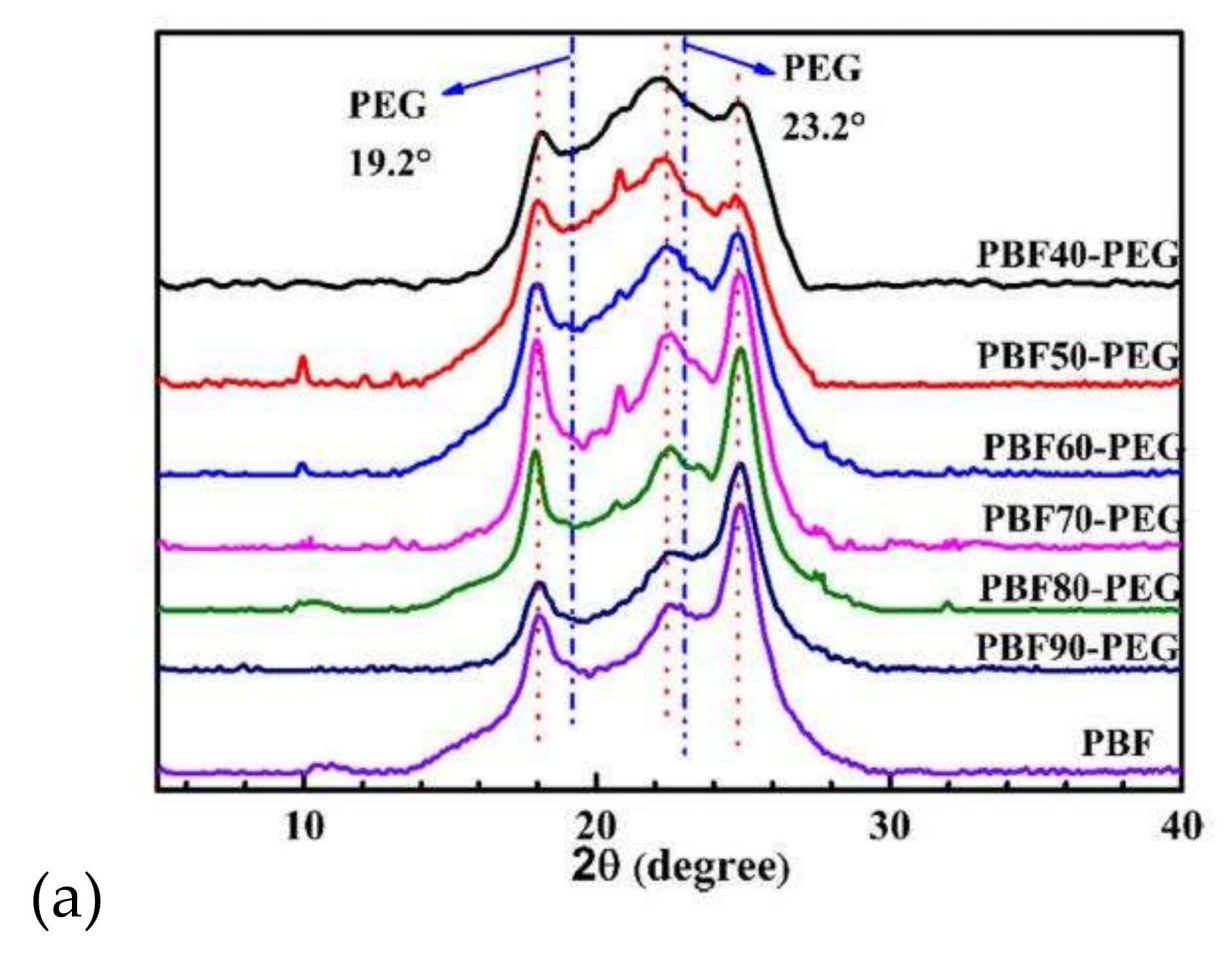

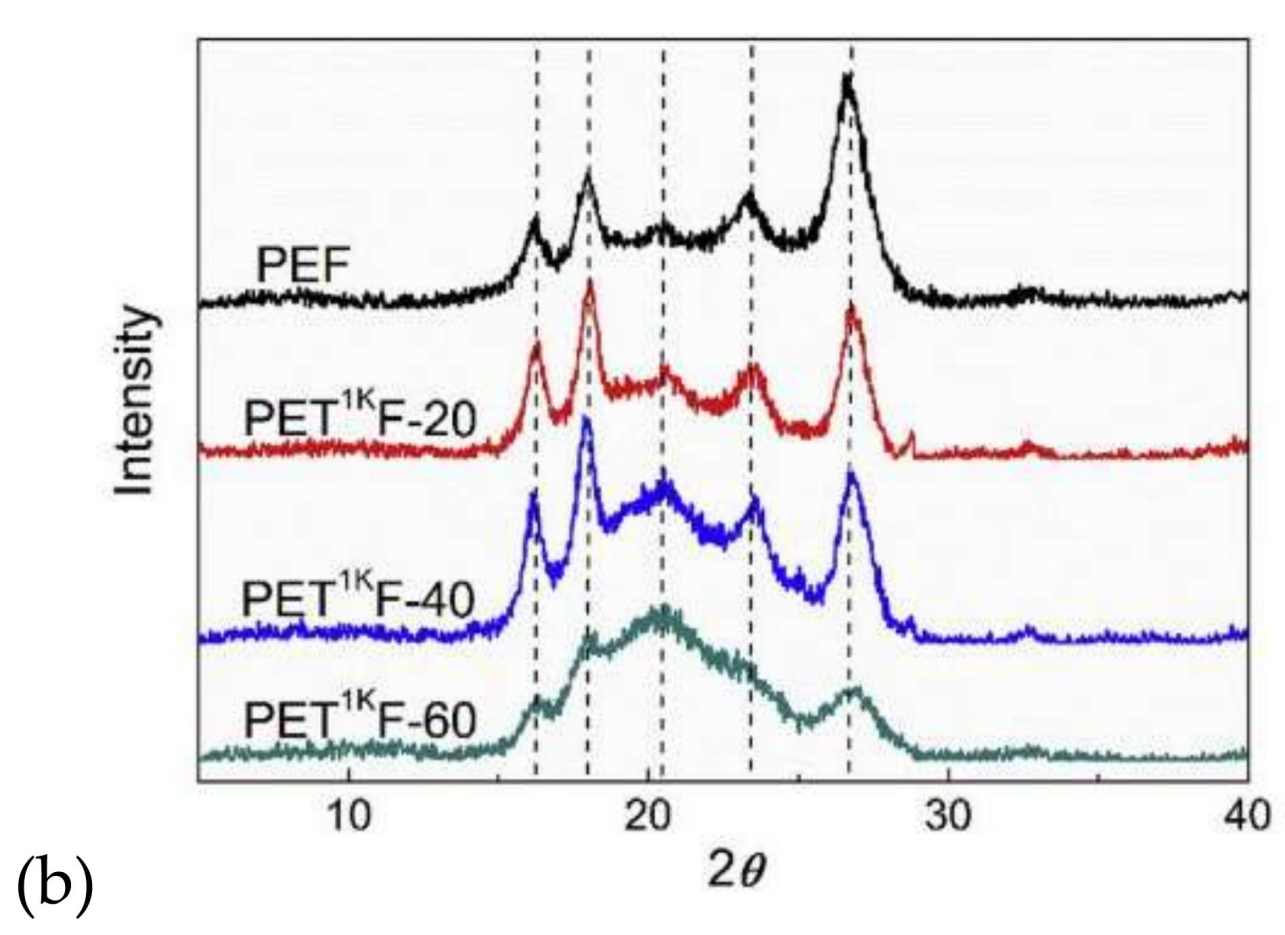
(**a**) XRD diffractograms of PBF-*co*-PEG block copolymers [89] and (**b**) PEF-*co*-PTMG block copolymers [56].

X-ray diffraction (XRD) results also confirm the above results, as in all cases, the diffractograms present the peaks of the furan homopolymer and with the introduction of higher contents of the soft segment, the intensity of the peaks is diminishing. Relative results are shown in Figure 5.

#### 3.2.2. Random Copolymers

Generally, in the case of copolymers where both components are able to crystallize, the crystallinity decreases with an increase of the minor component’s content, as a result of crystalline lattice incompatibility [113]. On the other hand, if there is compatibility between the crystallisable units within each crystal lattice, *co*-crystallization will occur. Two possibilities of *co*-crystallization exist: isodimorphism, which is observed in most cases of random copolymers and is associated with two crystalline phases and pseudo-eutectic behaviour [114]; and isomorphism, where there is one crystalline phase containing both comonomer units at all compositions [115]. With furan copolymers, isodimorphism is the dominant, if not the only *co*-crystallization phenomenon observed. In these cases, when low comonomer content materials can crystallize, crystallization happens by the exclusion of these segments from the crystal lattice of the dominant comonomer. In a series of works from our group, we investigated the copolymerization of one of the most important members of the furan family, PEF, with an aliphatic succinic acid copolymer for the preparation of PEF-*co*-PES random copolymers and with PET for the preparation of PEF-*co*-PET random copolymers. For the case of PEF-*co*-PES copolymers, the *T*_m_ and *T*_g_ were found to decrease with increasing ethylene succinate content, while an increase in the ethylene furanoate content caused a slight decrease in the cell dimensions of the copolymers. A pseudo-eutectic behavior, associated with isodimorphic *co*-crystallization, was also observed at an ethylene furanoate/ethylene succinate ratio of 35/65 (Figure 8). Likewise, for the PEF-*co*-PET random copolymers, the samples with the high terephthalate content crystallized faster, while the thermodynamic analysis of the *T*_m_ depression revealed that a small amount of the copolymer units was able to be introduced into the homopolymer. The broad *T*_m_ of the copolymers is an indication of their versatility, as it can be tuned in accordance with the planned application. Similar observations were made in biobased random copolyesters containing 2,5-FCDA units such as in the case of PBF-*co*-PBT [64], PEF-*co*-PBF [29], PEF-*co*-PESeb [51], PHF-*co*-PIsF [97], PBF-*co*-PImF [66], and PBF-*co*-PBC [84]. The random insertion of segments in the main chain of the copolyesters interrupts the packaging of the chains, and overall, the increase of the 2,5-FCDA content leads to slower crystallization rates and a large number of imperfect crystals. 

The incorporation of isomers in the case of copolymers disrupts the crystallization of the polymer chains. In the work of Thiyagarajan et al. [28], the authors prepared a series of copolyesters by combining two isomers of FDCA (2,5- and 2,4-FDCA) with linear aliphatic diols such as EG, PDO, and BDO. With respect to the *T*_g_, the DSC results revealed a synergetic effect of the combination of the two isomers, as the samples containing 5 to 15 mol% 2,4-FDCA displayed a *T*_g_ higher than that of 2,5-PEF. However, the copolymers did not show any cold crystallization features after quenching, as a result of the presence of the unsymmetrical 2,4-isomer that disrupts the 2,5-FDCA-based repeating units and subsequently the crystallization. The results are in agreement with the work by Bourdet et al. [116], which showed the criticality of the position of the carboxylic groups with respect to the furan ring on the ability of the materials to crystallize. Therefore, the integration of isomers can be used as a way to control the degree of crystallinity of homopolymers and create amorphous samples easily. 

**Figure 6:**
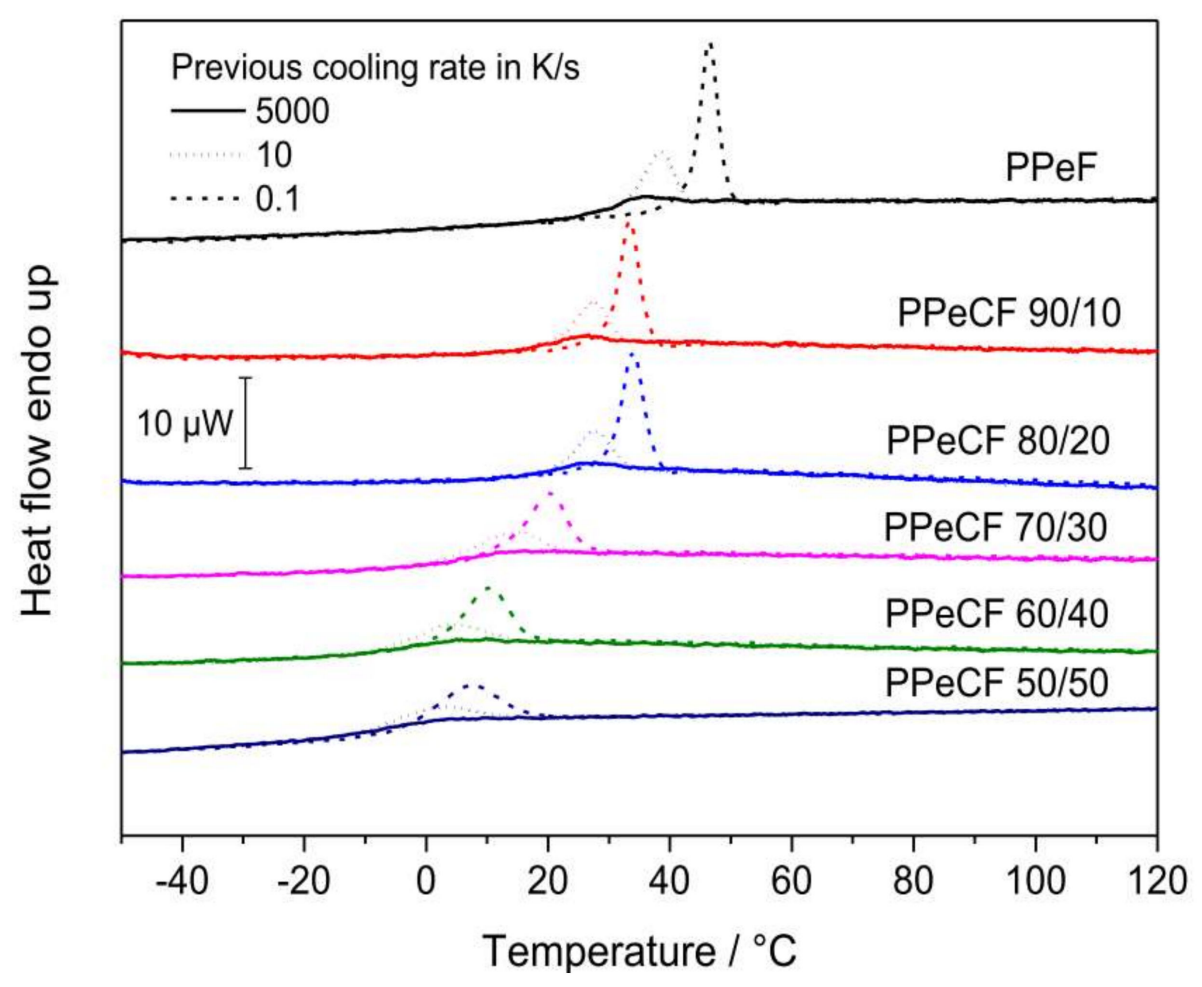
Fast scanning calorimetry (FSC) data of *T*_g_ for PPeF-*co*-PCL copolymers [95].

Besides conventional DSC, fast scanning calorimetry (FSC) has also been employed for the investigation of furan-based copolymers. Kasmi et al. [95] prepared PPeF-*co*-PCL and PHF-*co*-PCL copolymers by combining CL with PPeF and PHF with different molar ratios. As Figure 6 shows, the addition of CL enhanced the crystallinity and the crystallization rates, while it also reduced the *T*_g_ and the *T*_m_ of the copolymers.

Another important conclusion that came out from this study is that the flexibility provided by the CL units influences more extensively the less flexible pentylene polyesters compared to the hexamethylene ones.

**Figure 7:**
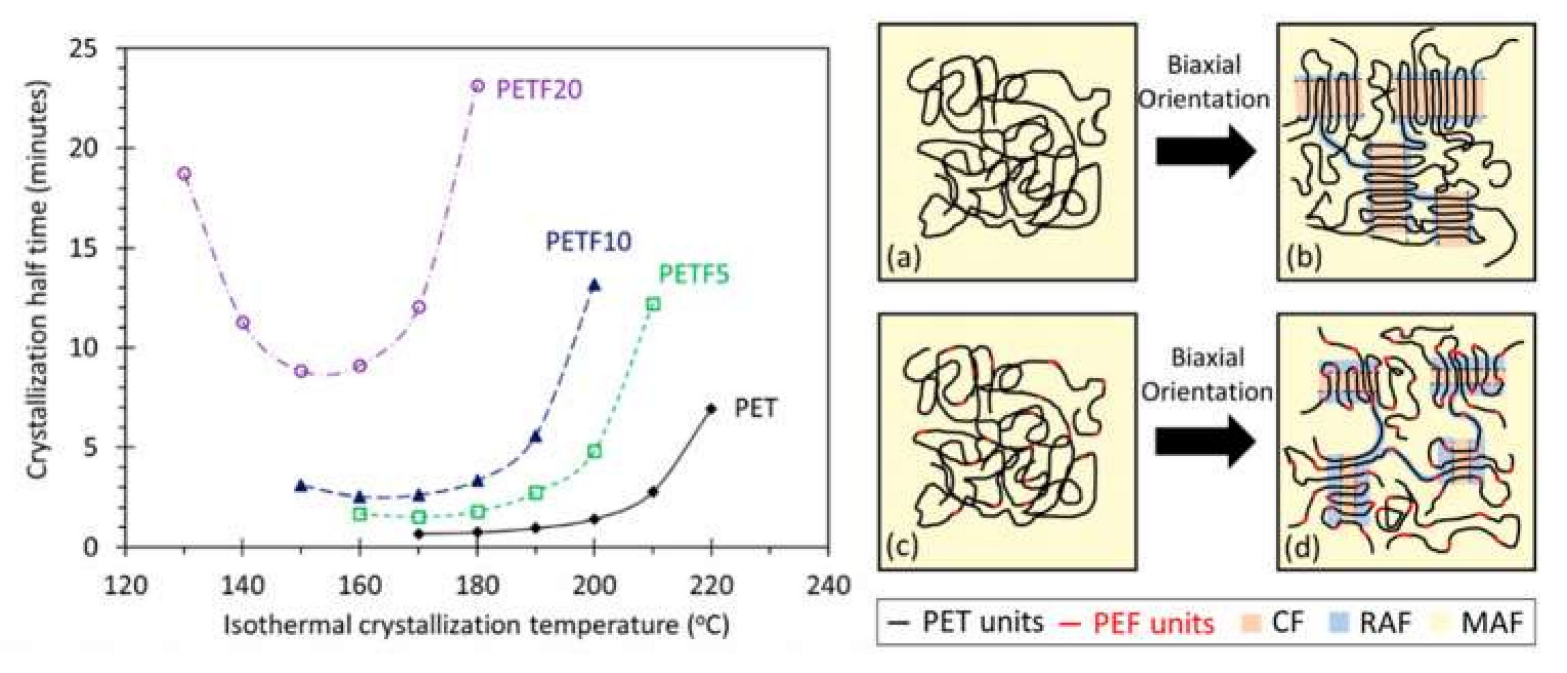
Crystallization and orientation of PEF-*co*-PET copolymers. Left: Crystallization half-times calculated from the DSC isotherms at different crystallization temperatures cooling from the melt for PET and PEF-*co*-PET copolymers with 5, 10 and 20% ethylene furanoate units (PETF5, PETF10 and PETF20 respectively). Right: Simplified schematic showing amorphous and crystalline fractions in chain folds and inter lamellar regions of (**a**) unoriented PET, (**b**) biaxially oriented PET, (**c**) unoriented PEF-*co*-PET random copolyester, and (**d**) biaxially oriented PEF-*co*-PET random copolyester; CF:crystalline fraction, RAF: rigid amorphous fraction, MAF: mobile amorphous fraction [41].

Finally, even though the majority of the published studies focused on the crystallization of the copolymers within the DSC chamber, Joshi et al. used strain-induced crystallization as a result of biaxial orientation to evaluate the percentage of rigid and amorphous fraction within their PEF-*co*-PET samples. In this case, in contrast to the results reported by Sun et al. [40] and Konstantopoulou et al. [39], the authors observed a decrease of the *T*_g_ as a result of the difference in Mw of the homopolymers and varying amounts of the more flexible diethylene glycol in the polymer backbone. After isothermal crystallization, the minimum values of crystallization half-time were reduced at lower temperatures with the introduction of increasing amount of PET within the polymeric backbone, since the *T*_m_ of the copolyesters was also reduced (Figure 7). The biaxial stretching of PET is well-known to lead to strain-induced crystallization as a result of the orientation of the polymer chains; however, given that it is faster than thermal crystallization, it leads to a higher number of imperfect crystals and an increase in the rigid amorphous phase between lamellae. For the case of the PET homopolymer, crystallization takes place due to the orientation of the mobile amorphous phase (Figure 7). On the other hand, the isodimorphic nature of the PEF-*co*-PET copolymers leads to the furan units acting as impurities within PET due to their random orientation and high stiffness, and the majority of the units are excluded from the crystalline phase. Therefore, during biaxial orientation, there is the suppression of segmental mobility due to the low temperatures, fast processes, and rigidness of the furan ring, which leads to the entrapment of a more rigid amorphous fraction in the chain folds and between lamellae, as shown in Figure 7. This process overall leads to an enhancement in the mechanical and barrier properties of the PEF-*co*-PET copolymer and is another indication that conventional PET materials can be replaced by their enhanced-performing copolymer counterparts. 

**Figure 8:**
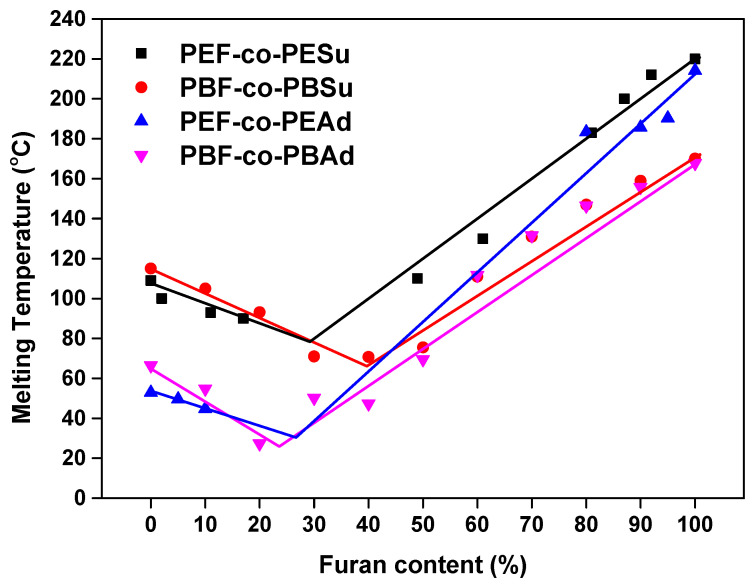
Eutectic behavior of alipharomatic copolymers [50,52,73,79]. The lines are just to guide the eye.

**Figure 9:**
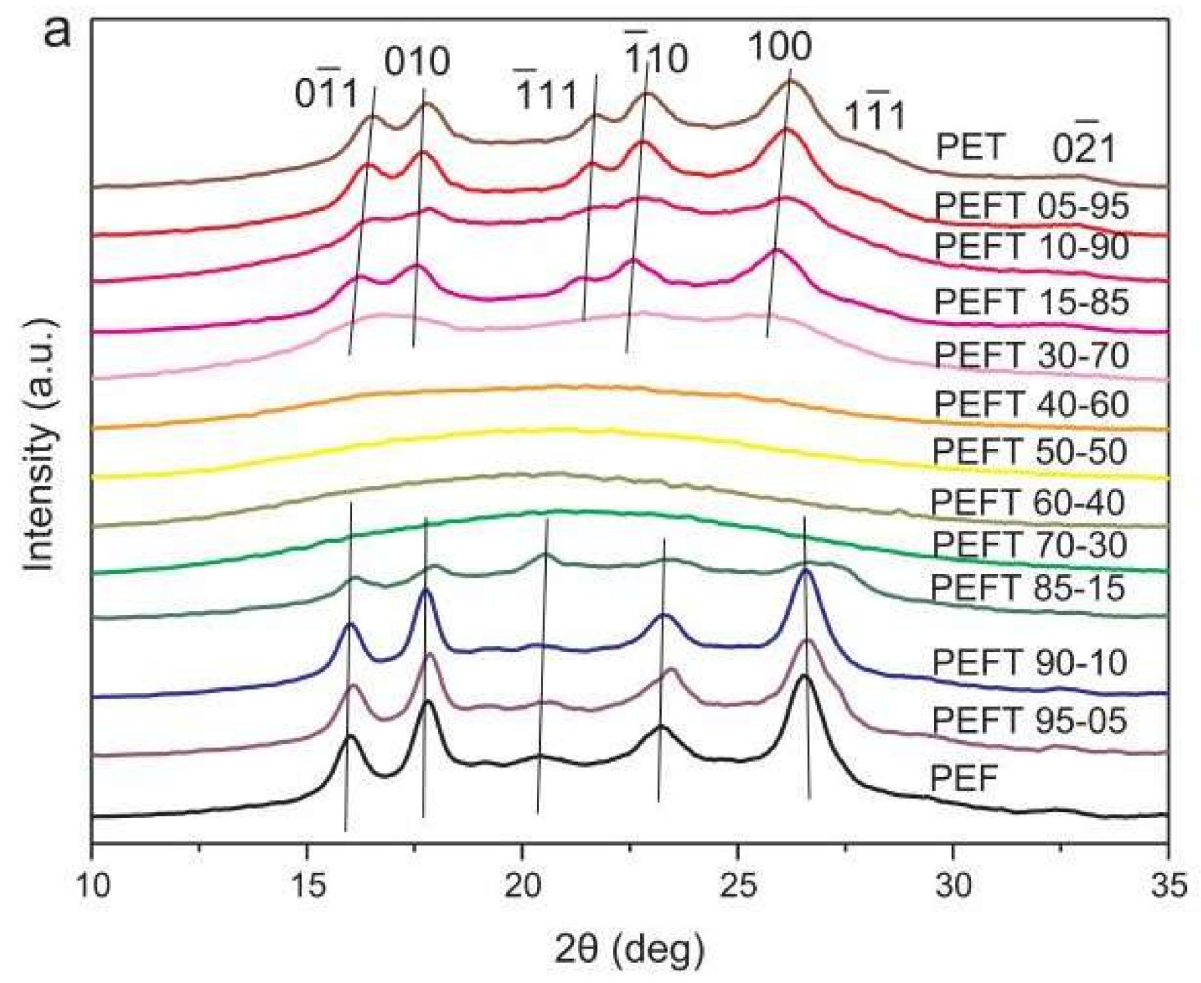
XRD diffraction patterns of random PEFT copolymers [39].

In summary, for furan copolymers, isodimorphism is the dominant, if not the only *co*-crystallization phenomenon observed. In these cases, when low comonomer content materials can crystallize, crystallization occurs by the exclusion of these segments from the crystal lattice of the dominant comonomer. However, this leads to a decrease of the lamellar thickness and thus to a depression of the *T*_m_. On the other hand, when the comonomer ratio evens out, the randomness of the polymer chains further hinders their ability to organize in a crystal structure, resulting in amorphous polymers. The structure of the comonomers can dictate the extend of this phenomenon, as it can be seen in the following graphs (Figure 8 and Figure 9). 

### 3.3. Thermal Stability 

**Figure 10:**
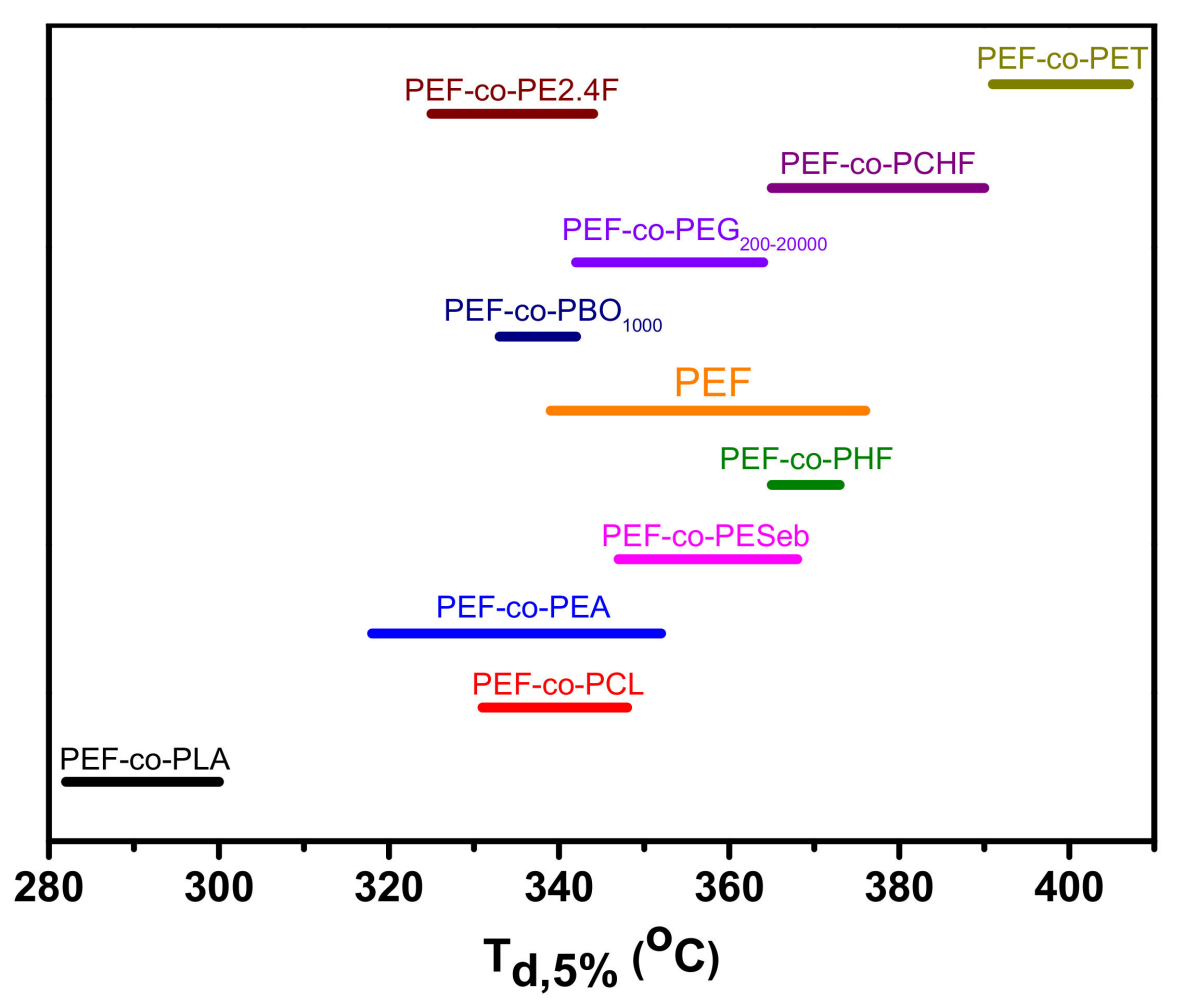
Thermal stability of PEF-based copolymers [28,39,45,49,50,51,54,56].

Thermal stability is another major factor to consider while designing a polymer, as it greatly affects its potential applications but also the conditions under which it is handled during manufacturing (e.g., extrusion, molding). While polymer properties such as Mw and crystallinity can affect the thermal degradation, the chemical structure also plays a major role. As it can be seen in Figure 10, while the PEF of different molecular weights from different studies had a temperature that corresponds to 5% mass loss (*T*_d, 5%_) ranging from 339 to 376 °C, PEF-derived copolymers resulted in an even broader *T*_d,5%_ range (Figure 10). In this section, copolymerization of PEF with different monomers will be assessed with regard to the thermal stability of the resulting materials, and their categorization will be attempted. 

#### 3.3.1. Copolymers with Cyclic Diols

**Figure 11:**
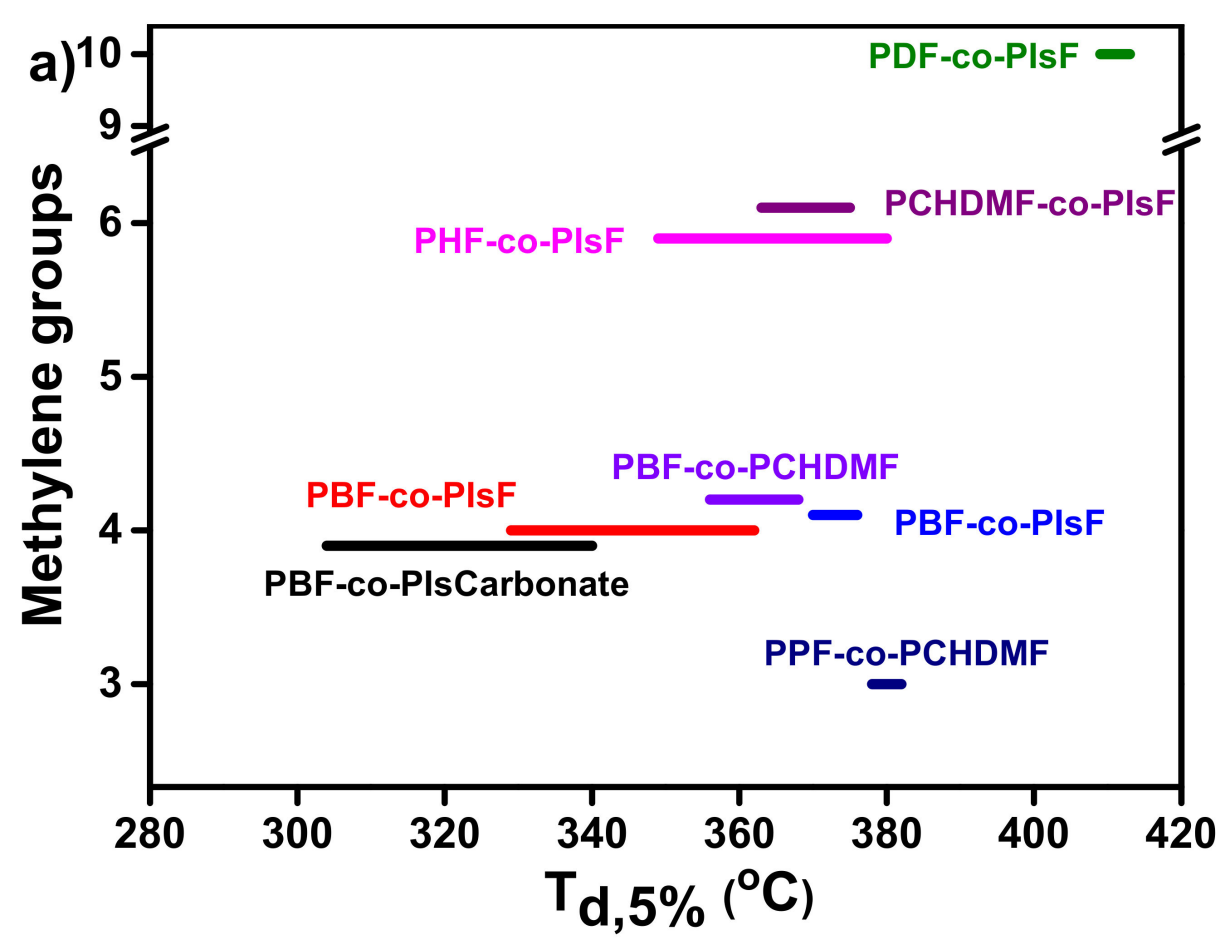

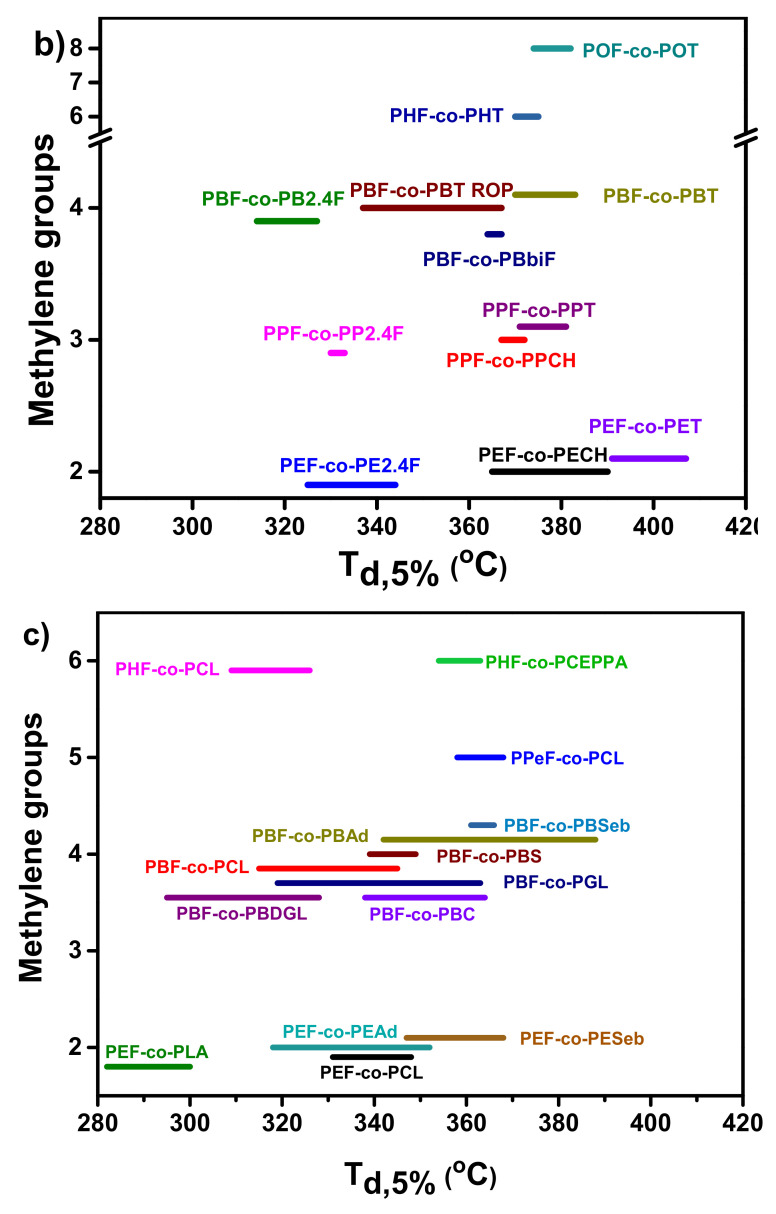
Effect of the number of methylene groups and the comonomer used on the T_d,5%_ of FDCA-based copolyesters: (**a**) with cyclic diols [59,65,68,70,97,99,100], (**b**) with cyclic dicarboxylic acids [28,42,51,61,63,64], and (**c**) with acyclic diacids and α hydroxy acids [49,50,51,73,77,78,84,86,95,96].

Cyclic diols have been used in several cases to modify the thermal properties of FDCA-based polyesters, as materials derived from such structures present high *T*_g_. Depending on the number of methylene groups of the polyester and the type of the cyclic diol, the thermal stability of the materials can also be controlled, and a wide range of thermal stabilities can be obtained. Cyclic diols that have been copolymerized with FDCA polyesters include CHDM, Is, and CBDO. CHDM was found to improve the thermal stability of PPF [59], PBF [59], and PIsF [99] as the starting decomposition temperature increased with increasing CHDM content. For the first two series of copolymers, the temperature at which degradation occurs with the fastest rate (*T*_d.max_) was also higher, which is in accordance with the CHDM content. PEF, PPF, and PBF [34] were copolymerized with low contents of CBDO (10 and 18 mol%), and the materials presented similar thermal stability with the furan homopolyesters. Finally, isosorbide was copolymerized with PBF [68], PHF, and PDF. PHF [97] and PDF copolymers presented enhanced thermal stability and higher *T*_d.max_ with the incorporation of isosorbide moieties in the polymer chains. PBF was copolymerized with both isosorbide and isosorbide carbonate with different outcomes. In one study [68], the copolymers’ thermal stability was improving with increasing isosorbide content, while in another study [70], isosorbide carbonate lowered the thermal stability of the materials. The addition of the carbonate linkage increased the reactivity of the secondary hydroxyl groups, but the resulting copolyester was less thermally stable. A summary of the effect of the different comonomers on the *T*_d,5%_ of FDCA-based polyesters is presented in Figure 11.

#### 3.3.2. Copolymers with Cyclic Diacids

Cyclic acids have also been utilized to produce materials with elevated thermal stability. FDCA polyesters have been acknowledged over the years as potential replacements of their terephthalic counterparts, and naturally their copolymerization has been studied in great extent. Other than that, 1,4 cyclohexane dicarboxylic acid and other furan derived acids such as 2,4-FDCA and bifuran dicarboxylic acid were also studied as potential comonomers. The copolymerization of FDCA with such compounds led to polymers with enhanced thermal stability compared to the neat furan polyesters provided in the respective studies. Copolymers of TPA and 2,5-FDCA with a variety of aliphatic diols were examined by Min et al. [42], and their thermal stability was exceptional. All materials’ degradation occurred in a single step above 350 °C, at intermediate temperatures compared to the respective homopolymers. TPA, 2,5-FDCA and BDO copolymers were also prepared by Morales Huerta et al. [64] by ROP, and while high Mw was achieved, the materials presented significantly lower decomposition temperatures compared to the ones on the study of Min et al. [42]. In the same work, a trend was observed concerning the decomposition initiation temperatures of the materials. As the length of the aliphatic diol increased from two to six methylene groups, the thermal stability lowered, but when it increased again from six to eight methylene groups, the materials showed improved thermal stability. Analogous results were found for the pristine PEF, PPF, PBF, and POF polyesters [117]. The same trend was observed in the study by Thiyagarajan et al. [28] concerning copolyesters based on 2,5 and 2,4 furan dimethylesters and short chain diols, namely EG, PDO, and BDO. The authors investigated the activation energy (E_a_) for thermal degradation via modulated-temperature–TGA measurements, and the defining factor for calculating E_a_ was found to be the length of the diol used. Finally, 1,4 cyclohexane dicarboxylic acid and bifuran dicarboxylic acid were used as comonomers in materials derived from PEF [33], PPF [61], and PBF [63]. The synthesized materials presented excellent thermal stability, with decomposition initiation temperatures above 360 °C that were between those of the respective homopolymers. The above are summarized in Figure 11b. 

#### 3.3.3. Copolymers with Acyclic Comonomers

One of the most challenging features in the development of novel polymeric materials is the combination of good mechanical properties with biodegradability. In this scope, 2,5-FDCA polyesters have been copolymerized with over 25 different aliphatic monomers to achieve the materials’ properties optimization. Three main categories stand out: copolymers with a,ω-dicarboxylic acids, ω-hydroxy carboxylic acids, and polyethers. The thermal stability of the resulting copolymers is concentrated in Figure 11c. 

Aiming to expand or refine the properties and/or potential applications of the parent homopolymers, aliphatic acids have been extensively used as comonomers for furan-based polyesters. Naturally, the addition of the aliphatic moieties in the polyester reduced the thermal stability of the materials compared to the neat polyesters. However, no specific trend can be observed regarding the structure of the comonomers, except that symmetrical monomers such as succinic [52,70,79,82] adipic [50,72,73], and sebacic acid [51] usually lead to materials of better stability compared to ω-hydroxy carboxylic acids such as lactic acid [54,55,98], CL [49,85,87,95], and glycolic acid [76,77,92]. The broad range of molecular weights of the synthesized materials leaves little room for any insightful deductions.

The incorporation of aliphatic polyols in furan polyesters has also been studied in depth to produce thermoplastic copolyester elastomers. PEF [46,47,48], PBF [88,89,93,94], and poly(neopentyl glycol 2,5-furandicarboxylate) (PNF) [101] have been copolymerized with PEG of various Mw, PPO_1000_, and PTMG_1000_. With the exception of one study [94], the long aliphatic chain polyethers lead to materials of lowered thermal stability compared to homopolyesters.

Finally, there is a number of studies involving furan polyesters derived from the combination of two aliphatic diols [50,71,102]. In those cases, the thermal stability of the materials depended on the monomer ratio, as the degradation temperatures were found to be between the respective degradation temperatures of the two homopolymers.

## 4. Mechanical and Thermomechanical Properties

### 4.1. Tensile Properties

**Figure 12:**
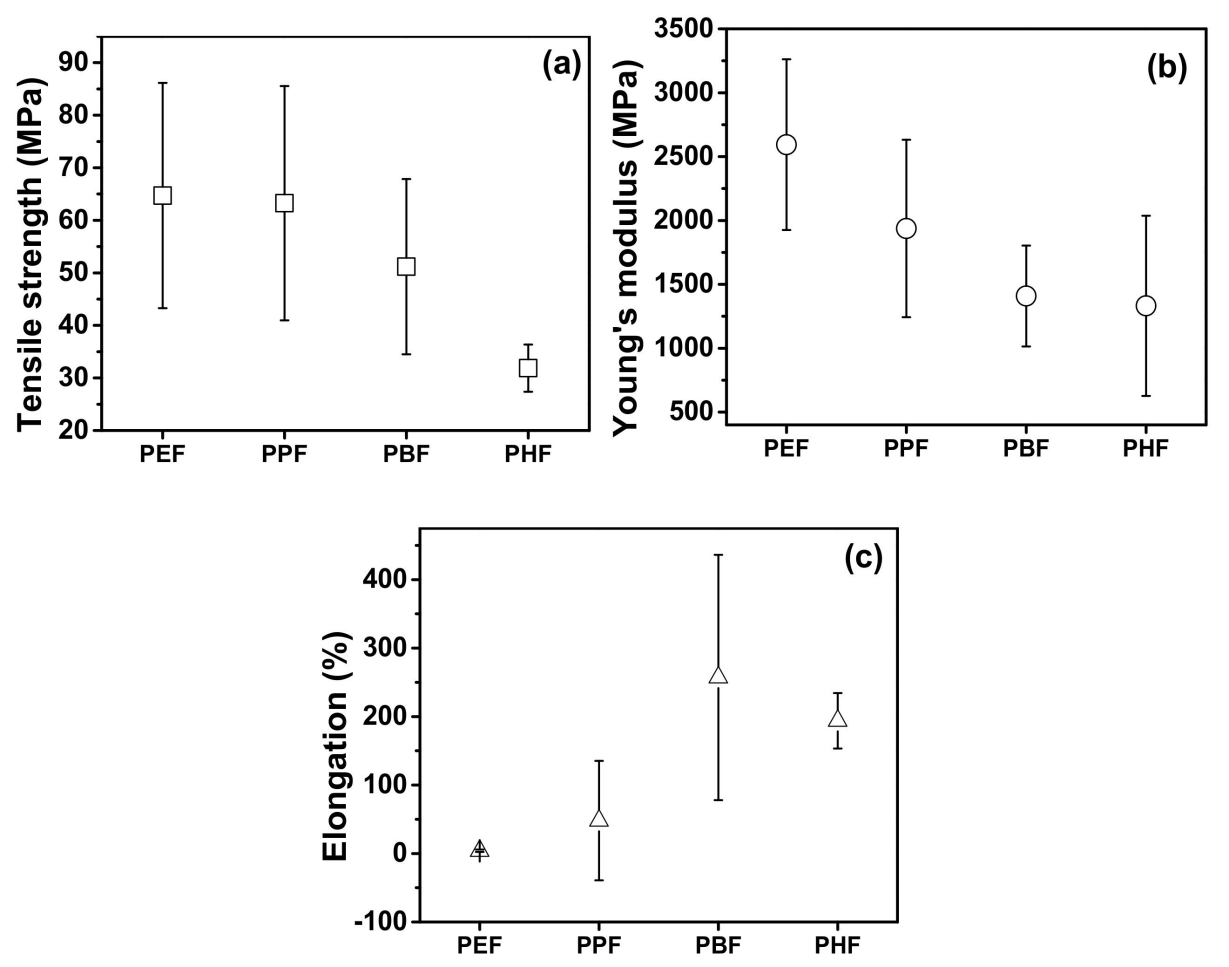
Summary of the effect of the number of methylene groups on the (**a**) tensile stress at break, (**b**) Young’s modulus and (**c**) elongation at break of FDCA-based polyesters (average values from Table A1.).

The applications of polymeric materials are directly linked to their mechanical properties. Tensile properties including tensile stress at break (*σ*_b_), Young’s modulus (*E*), and elongation at break (*ε*_b_) are the first mechanical properties to be evaluated, especially for polymers used as film packaging. There are various values reported on the tensile properties of FDCA-based homopolyesters, and they are summarized in Table A1. As those values depend heavily on molecular weight and crystallinity, the deviation between different publications is huge, so the mean values of *σ*_b_, *E*, and *ε*_b_ were calculated to try and determine the effect of the alkylene chain length on *σ*_b_, *ε*_b_, and *E* (Figure 12). Moreover, many studies did not report the crystallinity of the tested specimens that are usually prepared by injection or compression molding and can differ from the crystallinity calculated from the second heating step of DSC measurements usually reported, making the direct comparison between studies unreliable. However, in Figure 12, a trend on the tensile properties is observed when increasing the number of the methylene groups; *σ*_b_ and *E* decrease, while *ε*_b_ increases. Poly(alkylene 2,5-furandicarboxylate)s with small diols are stiff polymers because of the rigid furan ring. As the alkyl chain length increases, so does its mobility, resulting in softer and weaker polyesters.

#### 4.1.1. Copolysters of FDCA with Comonomers Containing Cyclic Units

Inserting PEF in the macromolecular chain of PET is a method applied to increase the biobased content and reduce the gas permeability of PET. The introduction of PEF moieties in PET did not significantly affect its *σ*_γ_ or *E*, in a wide range of compositions, and the stress–strain curves were typical for hard and tough polymers. *ε*_b_, on the other hand, decreased from 236 ± 35% down to 187 ± 20% at 20 mol% PEF content [40]. Joshi et al. investigated the tensile properties of both unoriented and biaxially oriented PETF copolymer films [41]. Unoriented PEFT had better *E* and tensile stress at yield (*σ*_γ_) values, and smaller *ε*_b_, even though their *X*_c_ was smaller. Oriented PEF-*co*-PET films had significantly increased *E* and *σ*_γ_ as well, again with slightly reduced *X*_c_ values. This peculiar behavior was explained through the increase of the rigid amorphous fraction caused by the PEF moieties. 

Rigid cyclic diols such as CHDM and CBDO have been explored as means to improve the toughness of furan-based polyesters. A PEF-*co*-PCHDMF copolymer showed *E* and *σ*_b_ but also an impressive increase of elongation of PEF up to approximately 3500%, which suggests better molecular flexibility. The copolymerization of either PEF, PPF, and PBF with PCHDMF or PCBDOF or their combination led to an increase of both *E* and *σ*_b_ and decrease of *ε*_b_ [33,34,36]. The PEF copolyesters with a CHDM:CBDO:EG molar ratio of 35:45:20 showed the best combination of tensile performance with *σ*_b_ = 88 MPa, *E* = 2140 MPa and *ε*_b_ = 67%, which is significantly improved in comparison with PEF [36]. Block copolymers of PPF and PPCH also had impressively increased elongation at break, and all tensile properties were found directly dependent on block length [61]. Large block lengths give larger percentages of crystallinity, which in turn results in larger *σ*_b_ and *E* values. 

**Figure 13:**
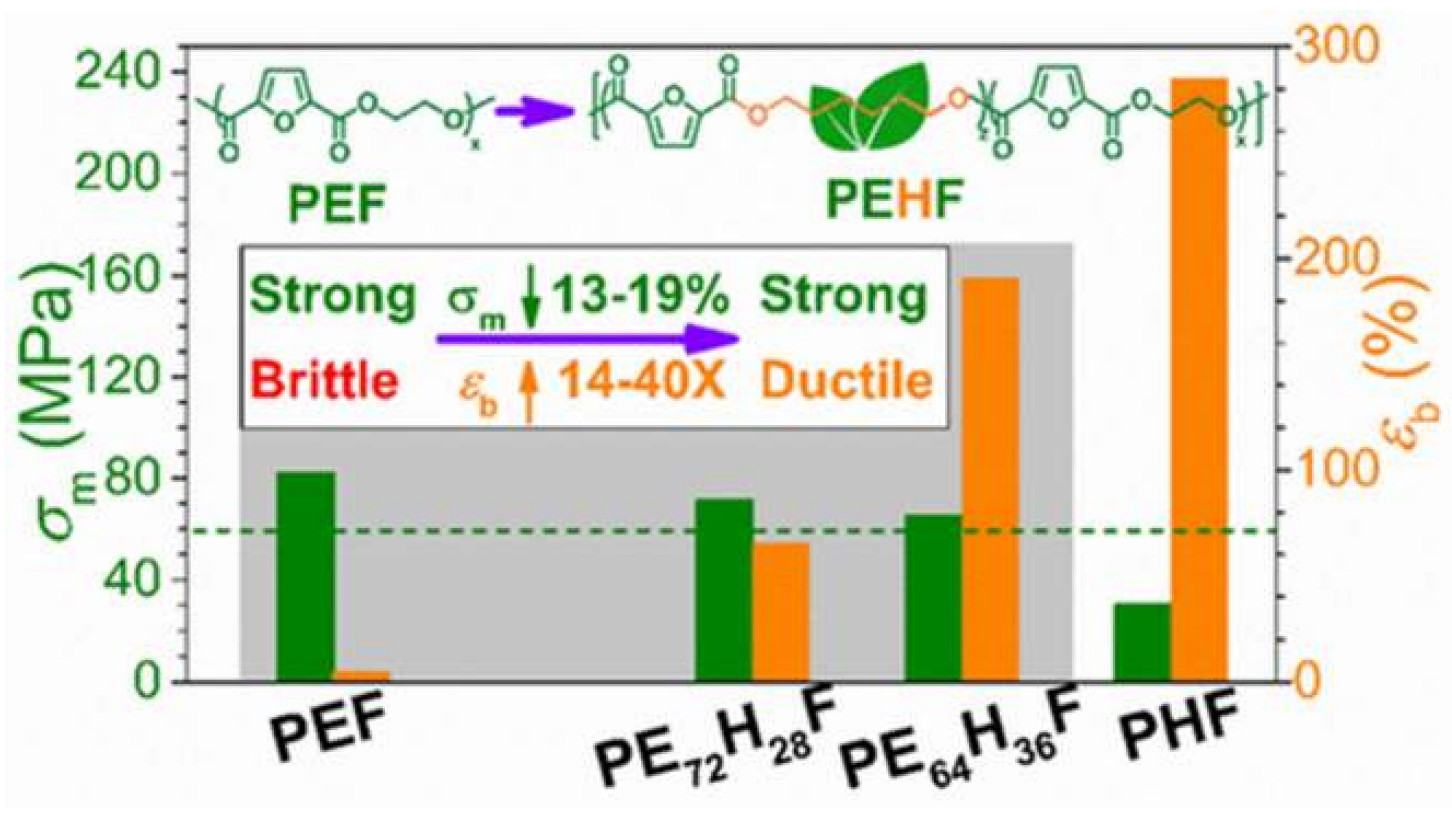
Effect of hexylene 2,5-furan dicarboxylate content on the mechanical properties of PEHF copolymers [45].

The combination of PEF, PPF, and PBF with other FDCA-based polymers has been reported as an approach to improve their mechanical properties [37,45,63]. Interestingly, small contents of PPeF and PHF yielded copolymers with tensile properties better than those of bottle-grade PET [37,45]. The effect of PEF–PHF composition on the tensile properties is presented in Figure 13.

Small amounts (6 and 10 mol%) of PPF copolymerized with PBF enhanced its tensile properties significantly, owning to the rigid nature of the PPF units, which seems to be a more important factor than its lower crystallinity [71].

To induce crystallizability in PIsF-based copolymers, Chebbi et al. inserted 1,10-decanediol in the polymerization mixture [100]. Low isosorbide contents seemed to enhance tensile properties, and the main factor that influenced them was concluded to be chain rigidity. The very large rigidity of PIsF was controlled also by copolymerization with PCL units [67], which gave copolymers with promising mechanical properties. For 50–80 mol% CL content, *σ*_β_ and *E* improved in comparison to neat PCL and in contrast with neat PIsF, which cannot be molded into testing specimens due to its brittleness, its copolymers were successfully compression molded. As the proportion of CL units increased, the polymers transformed from brittle thermoplastics without a yield point to brittle thermoplastics with a yield point, to a strong tough plastic with a yield point in CL content 80 mol%. The elongation and tensile strength of PIsF was also improved by the insertion of PBF units [68]. Quyang et al. found that when adding also dimethyl succinate and isosorbide carbonate units in PBF-*co*-PBS-*co*-PIsC copolymers, the tensile properties could be tuned [70]. In general, PIsF units are very efficient in improving thermal properties, but are rigid and of low molecular weight and tend to lead to the creation of brittle copolymers. A comparison of *σ*_b_, *E* and *ε*_b_ between PBF-*co*-PIC, and PBF-*co*-PIC-*co*-PBSu copolymers is presented in Table 2, which shows that a small amount of succinate units (10–20%) in PBF-*co*-PIC improves all mechanical properties. However, the single addition of isosorbide carbonate, even if it yields copolymers with similar [η] is incapable of enhancing the mechanical properties of PIsF-*co*-PBF. 

#### 4.1.2. Copolyesters of FDCA with Acyclic Comonomers

In contrast with cyclic comonomers, acyclic ones are used to counteract with the brittleness of PEF and PBF. EG and BDO are commonly used biodegradable comonomers for the production of ductile FDCA-containing copolymers [47,48,56,89,90,91,94]. When amorphous PEG of low Mw is used (1000–6000 g/mol), a decrease in tensile strength and Young’s modulus is observed along with the significant increase in elongation [47,56,89,91,94]. Bigger PEG segments (e.g., 10,000 and 20,000 g/mol) on the other hand reduce the *ε*_b_ and increase the *E* because of their high crystallinity [90]. 

The mechanical behavior of PBF-based copolymers with glycolic acid or diglycolic acid depended heavily on their composition [76,77,92]. These flexible comonomers, when added in small amounts, yield copolymers with ductile fracture and a yield point, while when added in large amounts, a rubber plateau appears instead, which is accompanied with a lower elastic modulus and tensile stress at break values. As the flexible diacid amount increases, the amorphous phase mobility decreases the *T*_g_ and the crystallinity and the copolymers transform from rigid plastics to soft elastomers.

**Figure 14:**
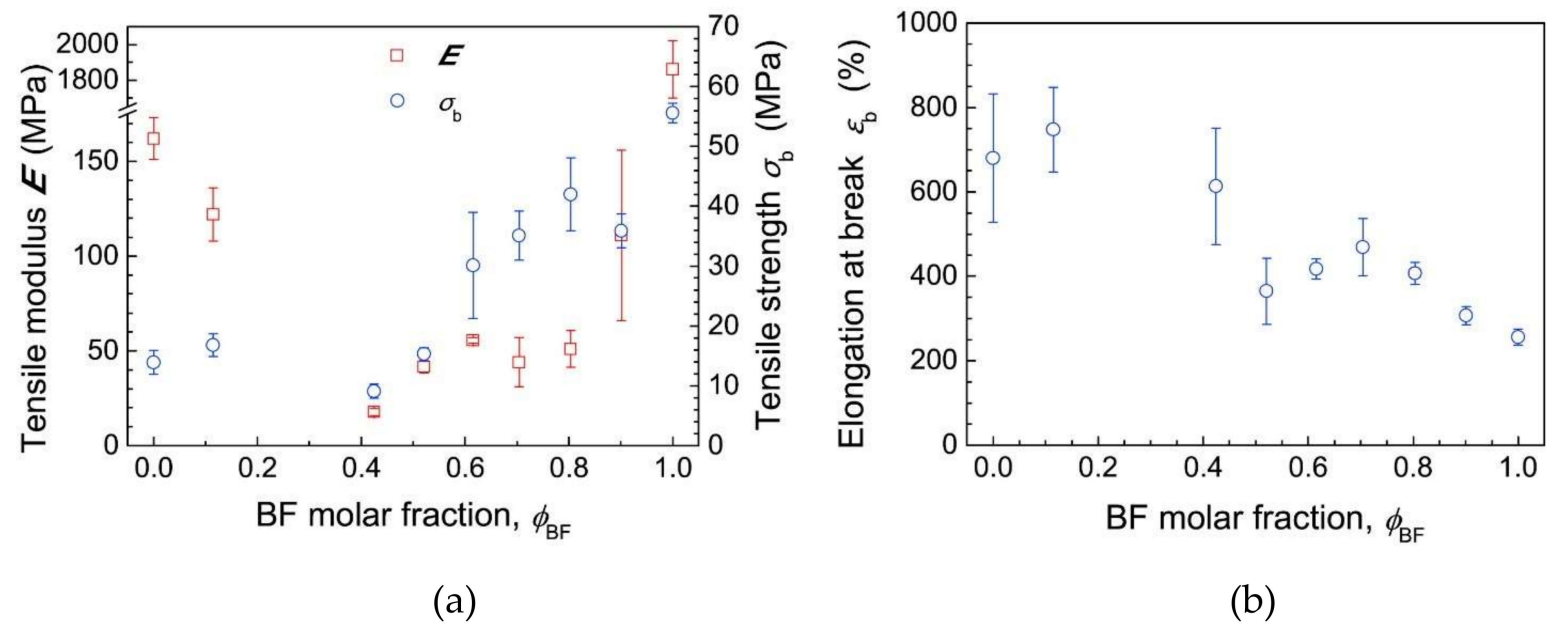
Composition dependences of the (**a**) Young’s modulus, tensile strength, and (**b**) elongation at break of PBF-*co*-PBAd copolyesters [73].

Crystallinity is a crucial factor that controls the tensile properties of polymers [62]. For example, the mechanical properties of PBF-*co*-PBS copolymers were found to depend on multiple factors besides composition (Mw, *T*_g_, crystallinity, and thermal history), explaining the different effect of small and large contents of butylene furanoate units on mechanical properties [79]. Small amounts of butylene furanoate in copolymers with succinate units decrease the overall crystallinity that causes a reduction in tensile strength and increase of elongation [79,82]. Copolymerization with adipic acid [73], caprolactone [49,87], and sebacic acid [51] also successfully improves elasticity and rebound resilience. When the molar ratio of the flexible units is large (>50%), the copolymers behave similar to thermoplastic elastomers. The effect of composition on the tensile properties of PBF-*co*-PBAd copolymers is presented in (Figure 14).

The rubbery behavior of PNF-*co*-PPTMGF copolymers was studied with cyclic tensile testing that revealed an increasing shape recovery ratio (up to over 90%) with increasing PTMG content [101].

**Figure 15:**
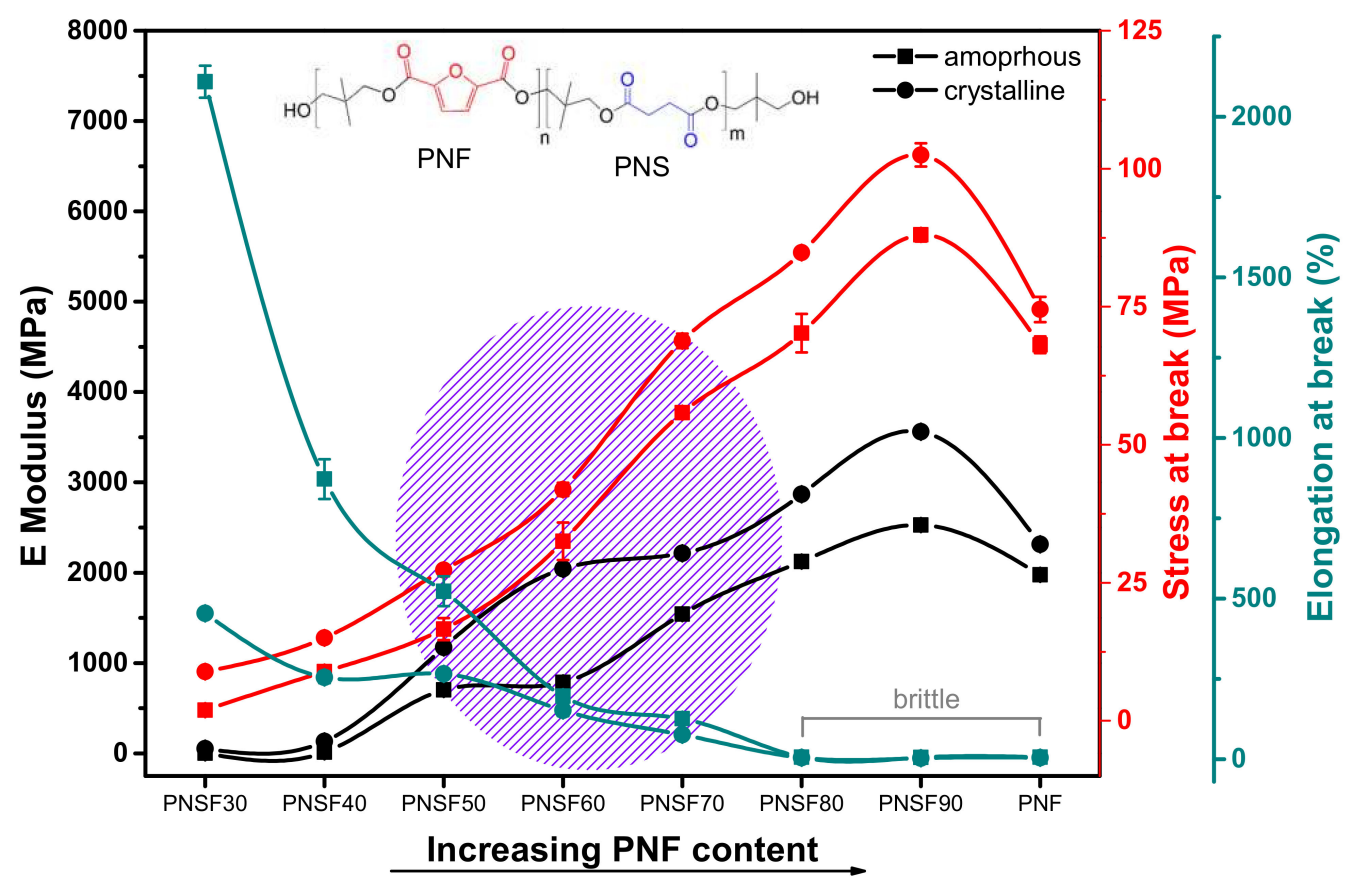
Effect of comonomer content on the tensile properties of amorphous and annealed PNF-*co*-PNS copolymers. The highlighted area shows the copolymers with adequate balance between the tensile properties. The lines are just a guide to the eye. Data adopted from Hu et al. [102].

PNF, when copolymerized PNS resulted in biodegradable polyesters with tunable mechanical properties [102]. The effect of the comonomer ration on *E*, *σ*_b_, and *ε*_b_ is presented in Figure 15. Increasing the PNF content increased *σ*_b_ and *E* and reduced the *ε*_b_. In all cases, thermal annealing provided all copolymers with enhanced strength. The ideal ratios were concluded to be PNSF50-PNSF70, as their *E* and *σ*_b_ values exceed those of most of the available biodegradable packaging polymers. 

### 4.2. Impact Properties

**Figure 16:**
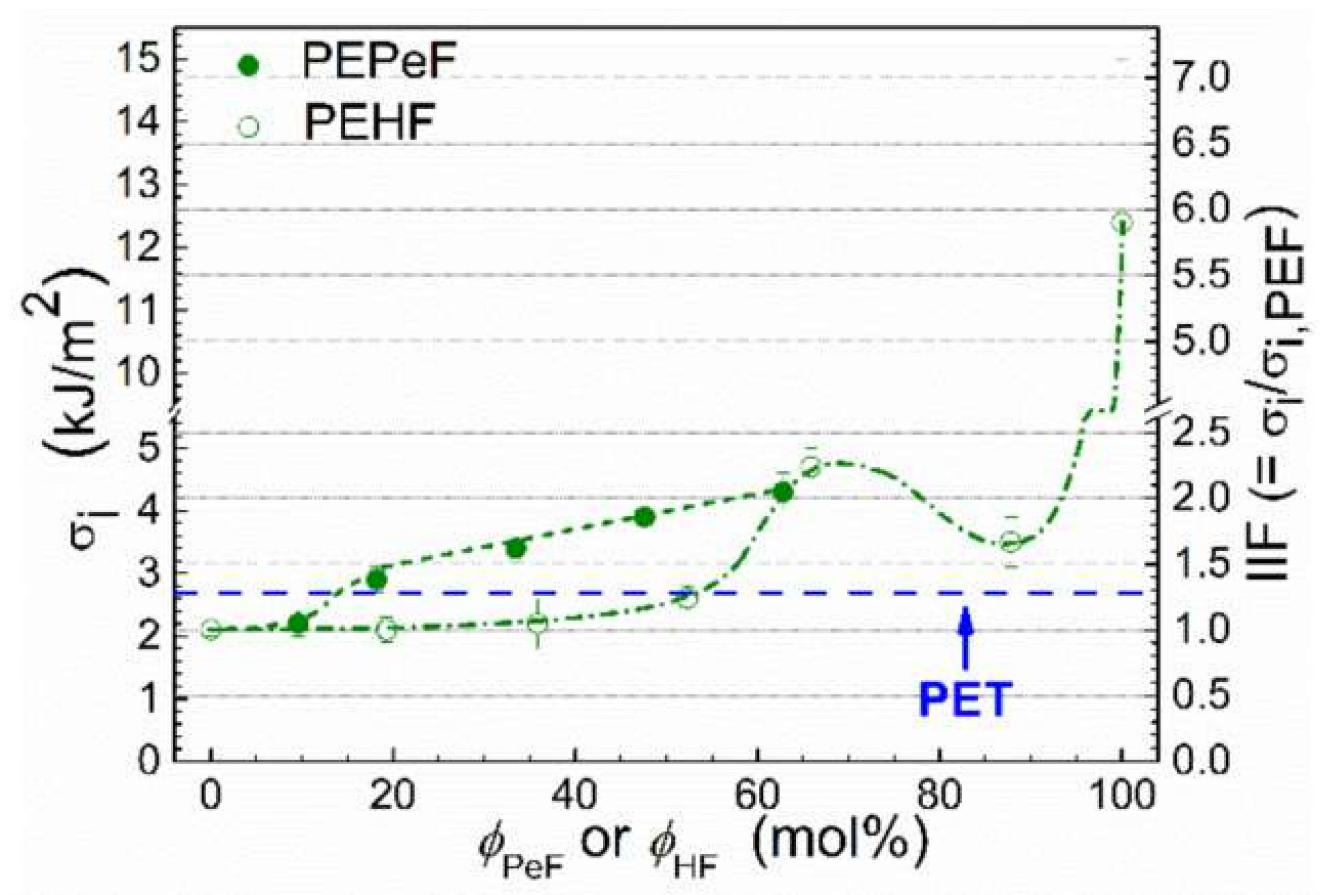
Composition dependence of the notched Izod impact strength (σ_i_) and impact improvement factor (IIF, defined as σ_i_/σ_i,PEF_) of PEF-*co*-PPeF and PEF-*co*-PHF copolyesters [37].

PEF-based copolymers with PTMG content ≥30 wt % displayed a 3-fold improvement of the notched izod impact strength, in comparison with PEF [56]. PBF-*co*-PEG_1000_ copolymers also presented excellent toughness during notched izod impact tests, as the specimens did not break for the samples with 60 wt % and 20 wt % PEG content, while the specimen with 10 wt % PEG had an impact strength value of 11.5 kJ/m^2^ [89]. The notched impact strength of PEF-*co*-PHF copolymers depended on their composition, with values being subject to the increase of the HF unit content, which was attributed to their ability to crystallize [45]. The incorporation of PPeF in PEF via copolymerization increased the impact strength of PEF from 2.1 up to 4.2 kJ/m^2^ [37]. In contrast, bottle-grade PET has a notched impact strength value of 2.7 kJ/m^2^. The dependence of impact strength on the composition of PEF-*co*-PPeF and PEF-*co*-PHF copolyesters is presented in Figure 16.

### 4.3. Dynamic Mechanical Analysis

DMA is a powerful tool for measuring transitions in polymers. It is estimated to be 100 times more sensitive to the glass transition than DSC, and it resolves other more localized transitions such as side chain movements that are not detected in the DSC. In addition, the technique allows the rapid scanning of a material’s modulus and viscosity as a function of temperature, strain, or frequency [118]. For furan-based polyesters, DMA analysis offers valuable information concerning the temperature where β and α transition occur, indicating the temperature range where the polyester possesses the stiffness to resist deformation and the flexibility to not shatter under strain [38]. 

**Figure 17:**
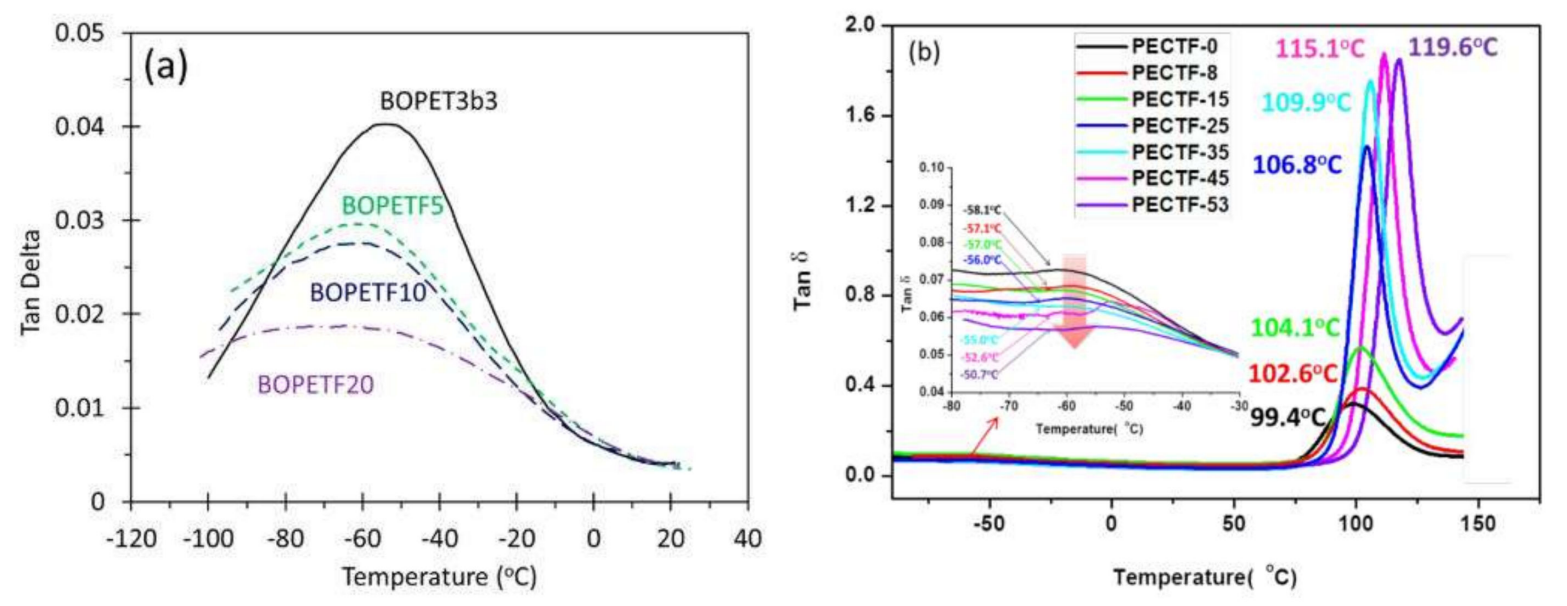
Influence of the introduction of FDCA on the polymer’s structure on (**a**) *T*_g_ [41] and (**b**) *T*_g_ and β relaxation [36].

The β transition is found at sub ambient temperatures and is related to the local motions or/and the reorientation of the carboxyl groups in the amorphous phase [70,84,89,90]. It has also been associated with the steric configuration transition of monomers such as CBDO and CHDM [36]. The furan ring displays a natural hindrance to motion due to the presence of the oxygen atom [16,18]. As a result of this feature, FDCA was introduced in PET to suppress the chain segment mobility. As Figure 17 shows, the restricted chain motion of the resulting copolymers resulted in tan δ peaks of reduced intensity compared to pristine PET [38,41].

On the other hand, for furan-based copolyesters, the introduction of a substituted or linear monomer in the polymer chain leads to increased chain motion, which can be detected by the increased intensity of the β relaxation peak in the graphs of tanδ as a function of temperature [70,84,89,102]. Other factors that contribute in the β relaxation of the polymer are its molecular weight and crystallinity. The increased intensity of the β relaxation is also an indication for enhanced gas permeability, but this will be addressed in a following section. 

**Figure 18:**
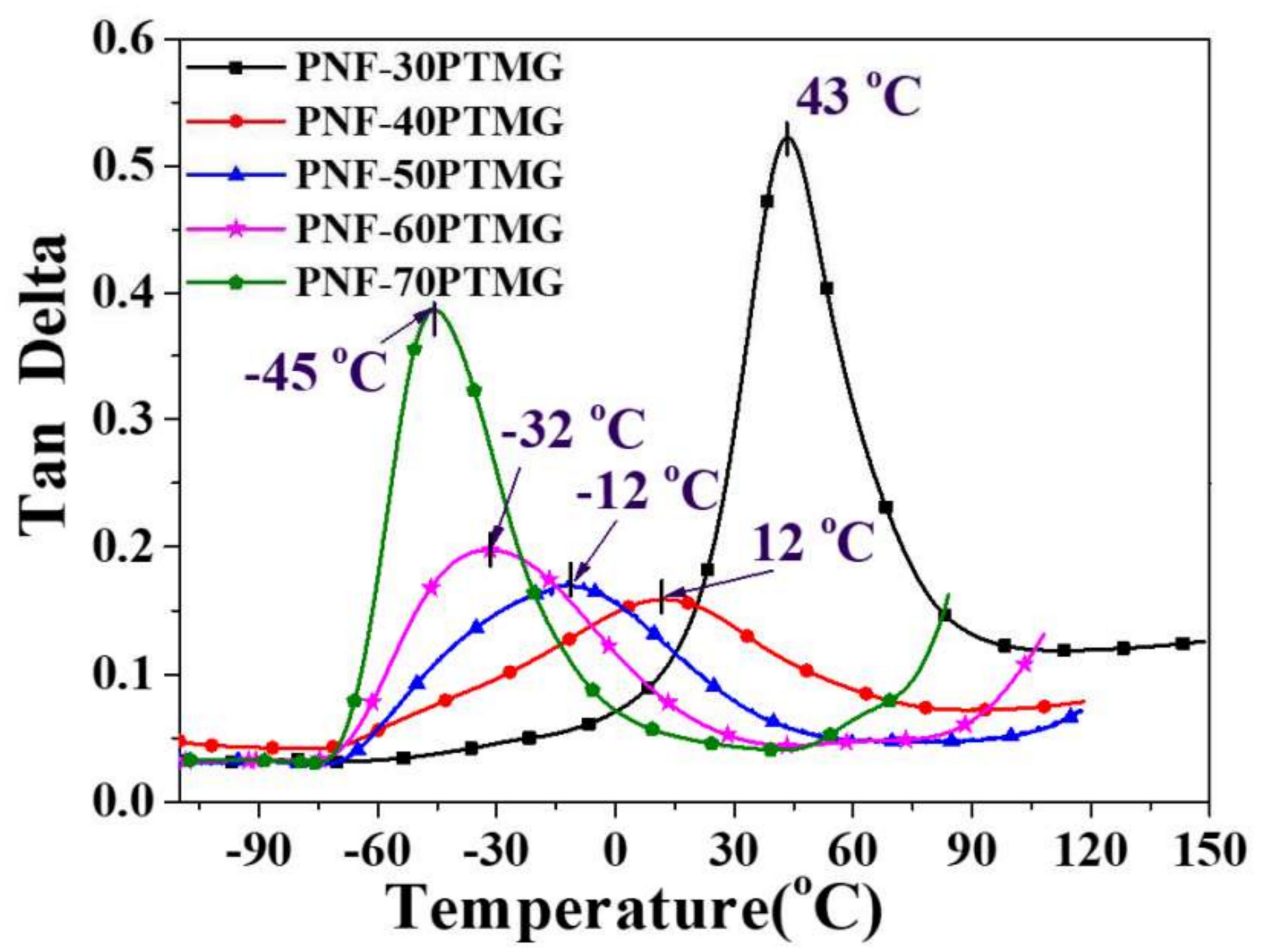
Evolution of α relaxation with the introduction of “soft” poly(tetramethylene glycol) (PTMG) segments in poly(neopentyl glycol 2,5-furandicarboxylate) (PNF) [101].

The other major information that can be extracted from DMA is the temperature of the α relaxation. It is expressed as a big loss in storage modulus (E’) or as the main peak of the tanδ versus temperature plot. It is often identified as the glass transition temperature (*T*_g_), indicating the threshold above which reorganization of the polymer chains takes place. However, significant differences compared to the *T*_g_ calculated with DSC can be obtained, up to 20 °C [102]. Depending on copolymer composition, the tanδ peak can offer valuable information. For example, it is very common to evaluate the influence of the flexible segment on the phase structure of block polyester-ether copolymers [58,88,90,93,101]. When a miscible continuous phase is formed, one clear α relaxation peak is expected, while for immiscible blocks, multiple peaks related to the *T*_g_ of each different block are anticipated. Simultaneously, the sharpness of the α relaxation indicates the homogeneity of the amorphous phase. If there is a dominant block in the composition of the copolymer, the tanδ peaks are sharp and narrow [58], while a broad peak appears when the amorphous phase consists of both polymer blocks [101]. The above are clearly depicted in Figure 18.

**Figure 19:**
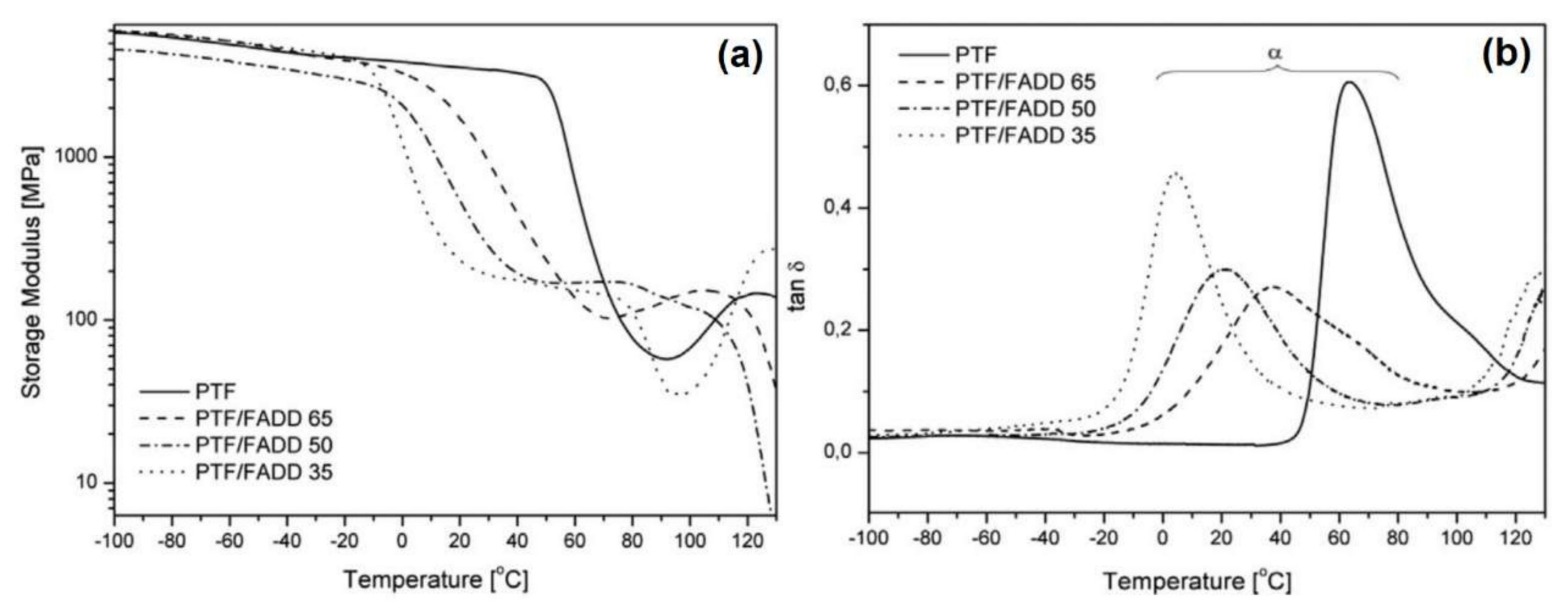
(**a**) Storage modulus and (**b**) tanδ graphs showing how crystallization phenomena can influence the intensity of the tanδ in PPF-*co*-PFADDF copolymers [58].

However, the intensity of the tanδ peak can be influenced by thermal transitions as well. For instance, in block copolymers, it is possible that two different thermal phenomena occur in the same temperature range—for example, the crystallization or the melting of the “soft” block and the glass transition of the “rigid” block. When this happens, an increase in storage modulus can be observed shortly after the big loss attributed to the *T*_g_ [90]. The distinction can be made by an increase of the storage modulus shortly after the big loss attributed to the *T*_g_. A clear example of how crystallization phenomena can alter the intensity of tanδ is given in Figure 19.

**Figure 20:**
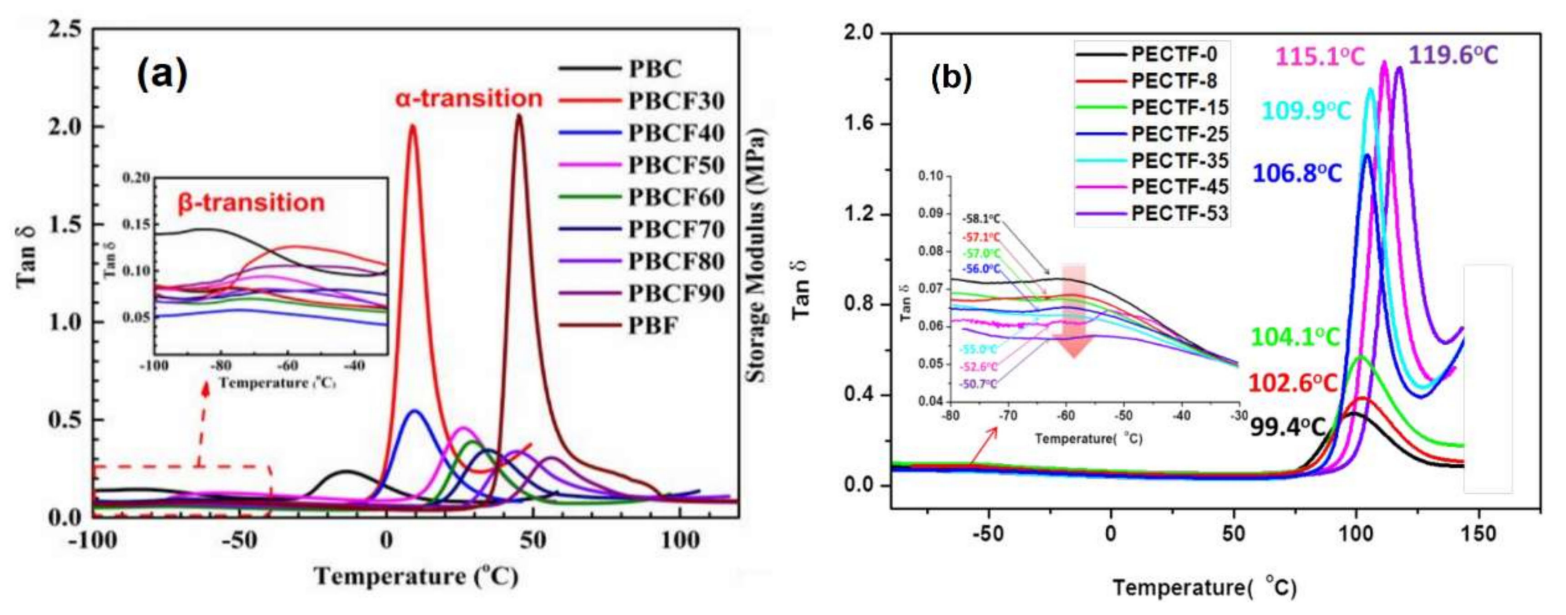
Shifting of α relaxation to higher temperatures with increasing “rigid” composition in (**a**) PBF-*co*-PBC [84] and (**b**) PEF-*co*-PCBDOF-*co*-PCHDMF copolymers [36].

Finally, for random copolymers, the rigidity of the structure can be evaluated from the shifting of α relaxation to higher temperatures. Usually, it is derived from the introduction of furan moieties, but other cyclic monomers were also found to have the same effect on increasing the polymers’ storage modulus [36,63,84]. For example, the shifting of the α relaxation with the insertion of furan [84] and CBDO [36] are shown in Figure 20.

## 5. Gas Barrier Properties

**Figure 21:**
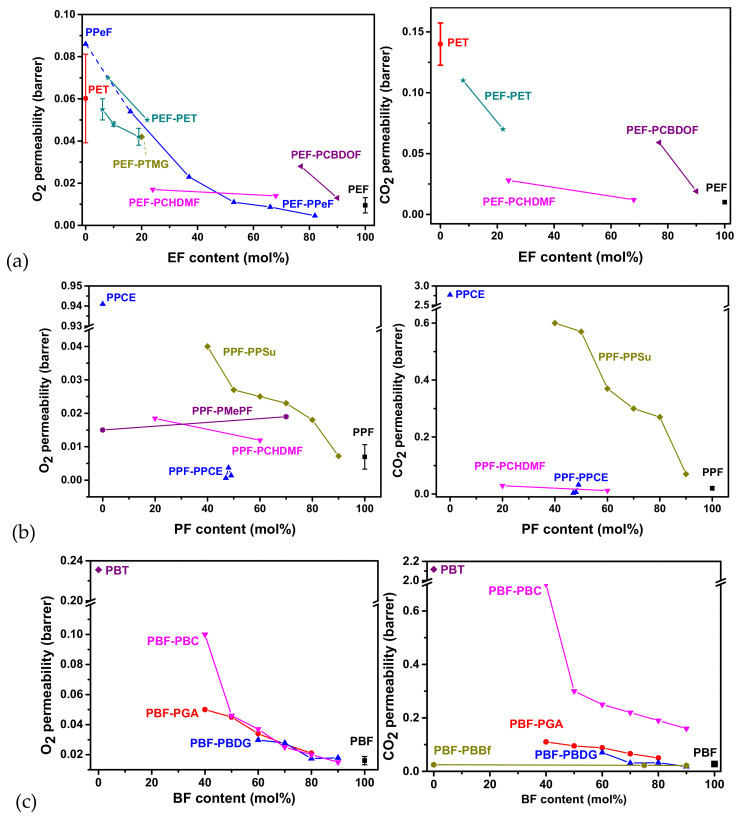
Effect of comonomer ratio on gas barrier properties of FDCA-based copolyesters (**a**) PEF, (**b**) PPF, and (**c**) PBF (data adopted from bibliography). Permeability of PBT values were obtained from [51].

A highly desirable and attractive feature of furan-based polyesters is their excellent gas barrier properties. Barrier properties depend on the chemical structure, the crystallinity, the Mw, and the thermal processing history of polymers [102]. The hindrance in the flipping of the furan ring causes a decrease in the diffusion coefficient, which provides FDCA-based polyesters with very low O_2_, CO_2_, and H_2_O permeability values. The polar interactions of the furan ring also contribute to the improved barrier performance. This superiority in the barrier properties in comparison with PET means that the potential furanoate polyester films will not require any additional layers for food packaging applications. In general, the published studies prove that copolymers with FDCA show improved barrier properties in comparison with the homopolymers without it. A summary of the findings is presented in Figure 21, where the effect of different comonomers on the O_2_ and CO_2_ permeability of PEF, PPF, and PBF is presented. 

One of the main reasons that led to the surge of research and intense efforts for the mass production of PEF is its impressively better gas barrier properties when compared with PET. PEF-*co*-PET copolymers exhibit reduced permeability in O_2_ and CO_2_, from 0.08 and 0.16 barrier (1 barrier = 10^−10^ cm^3^·cm/cm^2^·s·cmHg) of PET to 0.05 and 0.07 barrier with 20% EF units, respectively [40]. Joshi et al. measured the permeability of PEF-*co*-PET films before and after biaxial orientation, which reduced permeability, even if the crystallinity was reduced, because the rigid amorphous fraction and subsequently the number of furan units in the mobile amorphous phase increased [41]. Other polymers that exhibit good barrier properties when copolymerized with PEF are PCBDOF and PCHDMF. PCHDMF can improve the thermal properties of PEF, PPF, and PBF without significantly increasing its gas permeation values and exhibit improved barrier properties in comparison with PEN [30,34,35]. The linear comonomer BDO significantly increases the O_2_ permeability of PEF because of the flexibility of the PTMG soft segments [56].

PBF-*co*-PBbF amorphous films exhibited improved permeability to O_2_ in comparison with PBF, PBbF, PET, and PBT [63]. PBC units in PBF deteriorated the barrier properties while improving the ε_b_ [84]. PBF-*co*-PGA were prepared as potential replacements of commercial PBAd-*co*-PBT that has poor barrier properties, as glycolic acid has both excellent barrier properties and rigidity [92]. The copolymers were 53–118 times less permeable in CO_2_, up to 15 times less permeable in O_2_, and 6 times less permeable in H_2_O in comparison with PBAd-*co*-PBT. PBdGA in PBF did not affect heavily the barrier properties in small diglycolate contents, making them good candidates for packaging applications [77]. PBF-PEG copolymers showed better O_2_, CO_2_, and H_2_O barriers than commercial PBAd-*co*-PBT and PLLA [90].

The gas transmission rates of PPF-*b*-PPCH copolymers with short block lengths were smaller than those of both PPCH and PPF because of the crystallinity of the PPCH segment [61]. The high barrier properties were maintained in temperatures >*T*_g_. PPF-PCHDMF has smaller CO_2_ and O_2_ permeability than PET and PEN, but slightly larger than PPF [59]. The same effect was observed in PPF-*co*-PPS copolymers, with the permeability increasing while increasing the propylene succinate content [62]. PMePF had a slightly larger O_2_ permeability than PPF, and their copolymer was in between the two homopolymers [60], and it improved compared with commercial fossil-based polyesters. Succinic acid did not affect much the permeability of PNF in O_2_ and CO_2_ [102]. That was attributed to the steric hindrance the side methyl groups of neopentyl glycol that counteracts the flexibility of succinic acid. It was also noticed that increase in the molecular weight imparted smaller permeability values to the copolymers.

## 6. Optical Properties

One of the most important properties of a polymer when it comes to packaging applications is transparency. The transparency of polymer films depends mostly on crystallinity, since light scatters when it reaches crystalline regions. (Especially in the case of FDCA-based polyesters, transparency is a crucial parameter for their impending commercialization, because one of their main problems has been discoloration due to catalysts or low purity monomers). In the past, the coloration of FCDA-based polyester as a result of catalysts or side reactions (e.g., decarboxylation) or low-purity monomers has been considered as a limitation for their use in applications where transparency is required [119]; however, careful consideration of the synthesis conditions led to solutions in the coloration problems. For example, polymer blending has helped resolve this problem [120], along with the use of titanium (IV) isopropoxide or triphenyl phosphite during esterification [121]. In general, random copolymers tend to be more transparent than their corresponding homopolymers. PEF, PPF, and PBF films are hazy after thermal annealing with inadequate transparency [34].

**Figure 22:**
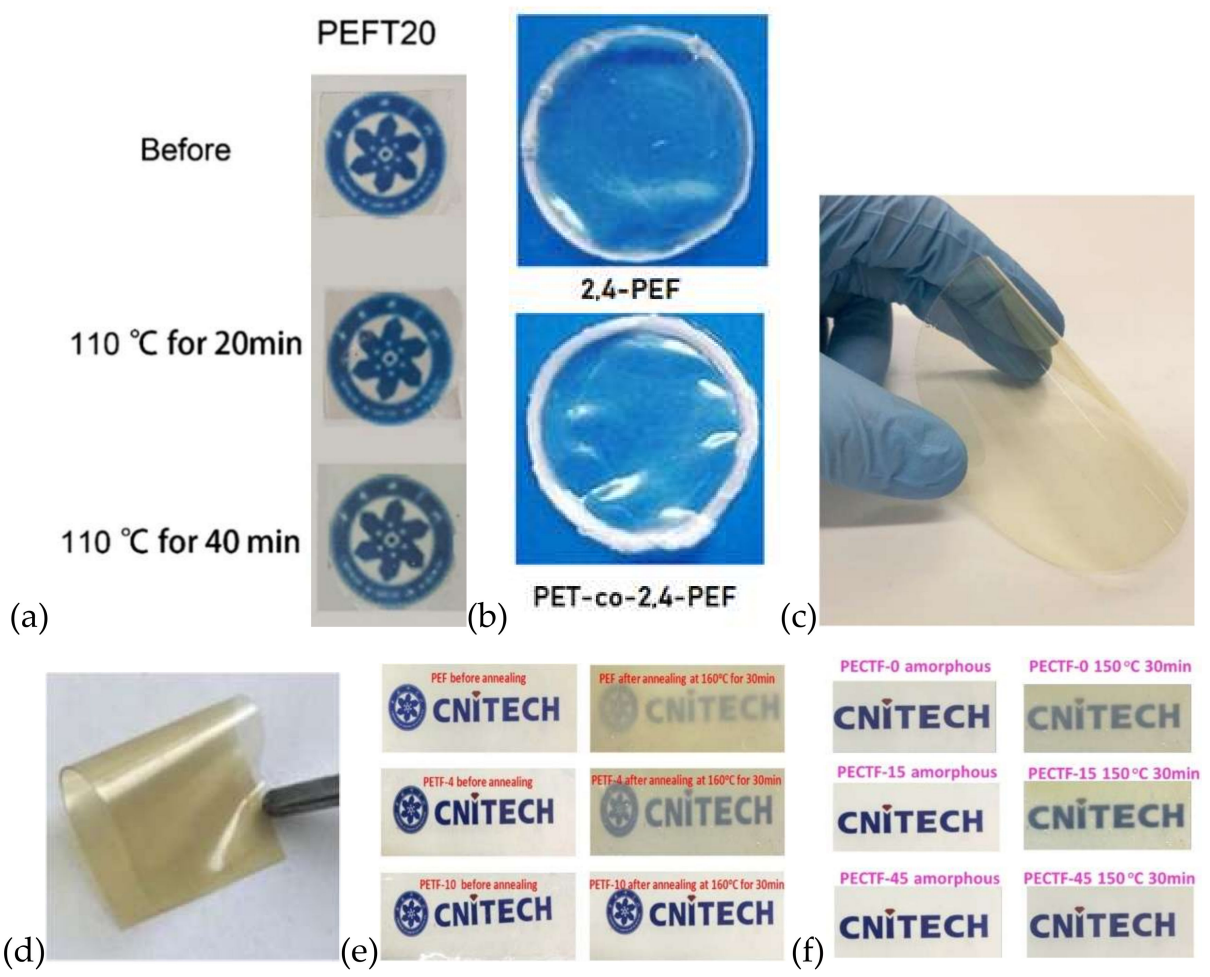
Images of films of various FDCA-based copolymers: (**a**) PEFT20 [40], (**b**) 2,4-PEF and PET-*co*-2,4-PEF [44], (**c**) PBF90Bf10 [63], (**d**) PPF-*co*-PPCH 90 [61], (**e**) PEF, PEF-*co*-PCBDOF copolymers [35], (**f**) PCDHDMF and PEF-*co*-PCDHDMF-*co*-PCBDOF copolymers [36].

PEFT20 copolymer films synthesized from DMFD and dimethyl terephthalate, with catalyst Sb_2_O_3_ and temperature up to 270 °C were transparent both before and after annealing (Figure 22a), in contrast with PET film, which became opaque after annealing [40]. Unlike 2,5-PEF, 2,4-PEF homopolyester and PET-*co*-2,4-PEF (10/90–(50/50) copolyesters synthesized from DMFD with catalyst titanium(IV) isopropoxide and at temperature up to 210 °C were transparent (Figure 22b), which was because of their amorphous nature [44]. PBF-*co*-PBbF copolymers exhibited both excellent transmittance and UV absorption values because of their conjugated bifuran moieties [63]. However, as seen in Figure 22c the copolymer has a light-yellow color, as opposed to a PBF homopolymer with comparable purity. The synthesis of these particular polymers took place with TBT at temperature up to 250 °C with monomer DMFD. PPF-*co*-PPCH films (Figure 22d) were also more yellow than their corresponding homopolymers when synthesized at 240 °C from FDCA 98% with TBT catalyst [61]. The low purity of the monomer can explain their discoloration.

PPF-*co*-PCHDMF 20/80 synthesized with DMFD had acceptable optical properties that were similar of those of PET with a little higher haze [59]. PEF-*co*-PCBDOF copolymers (Figure 22e) were colorless and transparent before annealing, and after annealing, only the one with 10 mol% CDBO content remained transparent [35]. Films of PPF-*co*-PCBDOF were also transparent and with no discoloration before and after annealing, but PBF-*co*-PCBDOF crystallized enough to become hazy [34]. PEF-*co*-PCDHDMF-*co*-PCBDOF copolymers (Figure 22f) were clear before thermal treatment, and after heating at 150 °C for 30 min, only the one with a CHDM:CBDO:EG ratio of 35:45:20 remained transparent [36]. All the colorless copolymers reported were synthesized from DMFD with catalysts zinc acetate and/or Sb_2_O_3_.

## 7. Biodegradation 

According to the International Union of Pure and Applied Chemistry (IUPAC), a biodegradable polymer is a “polymer susceptible to degradation by biological activity, with the degradation accompanied by a lowering of its molar mass” [122]. Biobased polymers can be either biodegradable (e.g., PLA, PHA, poly(alkylene succinates) etc.) or non-biodegradable, such as FDCA-based homopolyesters and other drop-in plastics (bio-PET, bio-PE). Scientists have been exploring biodegradable polymers as a solution to the plastic waste accumulation problem, envisioning the design of biobased and biodegradable polymers with tuned degradation rates depending on the application. Ideally, biodegradable plastic waste would be collected along with organic waste and would be composted, but this requires the regulation of standardized sorting and microbial degradation procedures with the aim of minimizing their carbon and energy footprints [123].

The most prevalent strategy for turning non-degradable polyesters to degradable ones is their copolymerization with aliphatic dicarboxylic acids, as aliphatic polyesters degrade through hydrolytic mechanisms. The main factors that affect hydrolytic degradation are the chemical structure, molecular weight, morphology, crystallinity, hydrophilicity, and temperature [124]. Polymer biodegradation is usually estimated in the laboratory through the mass loss in specific media, and sometimes the reduction in molecular weight is also measured. However, a lack of consistency makes the comparison between different studies and the drawing of generalized conclusions difficult. Nowadays, there are standardized protocols by the ISO and the ASTM, so the biodegradation methods used by different research groups should become more and more consistent [125]. 

Νon-biodegradable, alipharomatic polyesters such as PET and PEF can be hydrolyzed by specific enzymes [126,127]. PEF with different Mws was successfully hydrolyzed by Cutinase 1 from *Thermobifida cellulosilytica*, opening the possibility for the functionalization and recycling of monomers, as FDCA and its oligomers were released during incubation [128]. The biodegradation conditions and the percentage of maximum mass loss values of all FDCA-based copolymers reported in bibliography are presented in Table A2.

**Figure 23:**
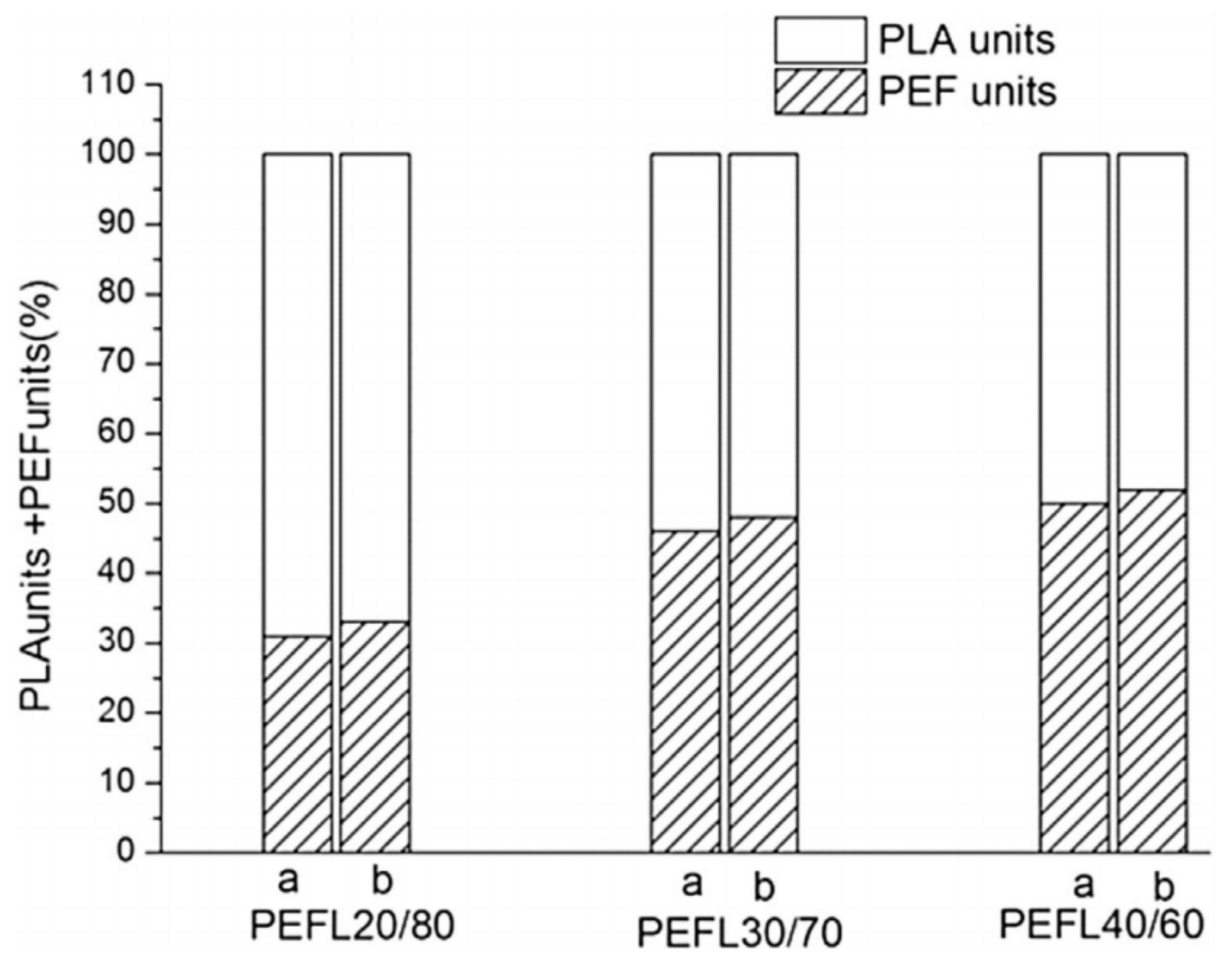
Mole fraction of lactide and furan units in soil before (**a**) and after (**b**) degradation [55].

The first attempt to provide PEF with biodegradability was in 2014 by copolymerizing it with PLA [54]. The PEF-*co*-PLA copolymers were significantly hydrolyzed in SBF over a period of 12 weeks, reaching 60% for 93 mol% lactide. Their Mw might have contributed to this fast mass loss as it was in the range of 7000–8000 g/mol. The degradation rate of the copolymers seemed to depend more on the lactide content, and as a result, the smaller *T*_g_ and higher water absorption rather than crystallinity. The degradation of PEF-*co*-PLA copolymers with 60, 70, and 80 mol% lactide was studied both in simulated body fluid (SBF) and garden soil [55]. The Mw of these copolymers was higher, from 70,000 to 130,000, so its weight loss in SBF was smaller (up to 25% after 55 days), while in soil mass loss reached approximately 65%. As expected, increasing the amount of PLA units increased its mass loss rates, but interestingly enough, after calculating the ratio of lactic and furanic units after degradation (Figure 23), it seemed that the cleavage of esters randomly took place in both comonomers. 

**Figure 24:**
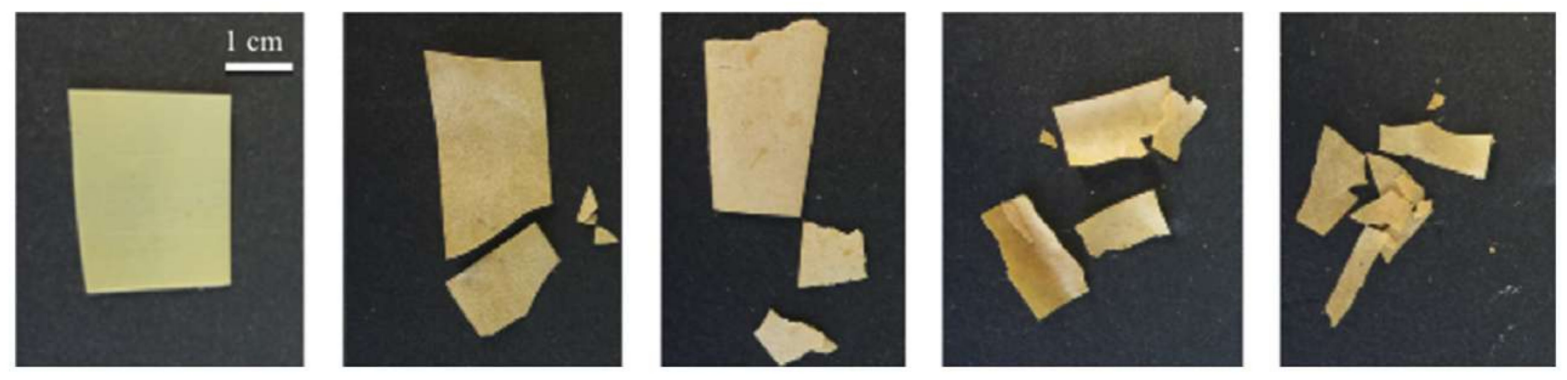
Pictures of the polymeric films recovered after 21 days of incubation in compost. From the left to the right: PBF, P(BF90BDG10), P(BF80BDG20), P(BF70BDG30), and P(BF60BDG40) [77].

PGA, which is similar to PLA but without the side methyl group, was copolymerized with PBF and could successfully induce degradation [77,92]. It was selected because it has better barrier properties and is more sensitive to hydrolysis than PLA and PCL. The non-specific scission of the esters of PGA cause the PBF-*co*-PGA copolymers with Mw = 6850–8950 g/mol to lose up to 60% of their weight under enzymatic hydrolysis [92]. When using diglycolic acid instead of glycolic acid, Soccio et al. found that its copolymers with PBF were more hydrophilic and compostable (Figure 24) in comparison with PBF, while crystallinity increased as the degradation progressed, proving that amorphous regions are more prone to the attack of microorganisms [77].

PEG is another linear polymer that can provide other polyesters with biodegradability. Small amounts of PEG induced biodegradability in PEF [48] and PBF [89,90], which depends on the Mw of PEG and its molar content in the copolymers. The PEG moiety increases the hydrophilicity of the copolyesters and subsequently its water uptake, leading to polymers susceptible in the degradation in water and soil. PPPOF on the other hand increased the mass loss rate of PBF-*co*-PPPOF insignificantly after 12 weeks in either phosphate buffer solution (PhBS) or PhBS with lipase [93]. This could be due to the side methyl group of PPO that could be preventing the enzymes and water to reach the sensitive to hydrolysis ester bond.

Succinic acid is another biobased dicarboxylic acid of great interest as it can yield biodegradable polyesters with attractive properties. Consequently, it has been employed as a biodegradable and biobased comonomer in FDCA-based polyesters with promising results. PEF-*co*-PES copolymers exhibited weight loss in PhBS/lipase up to 12.5 wt % after 1 month [52] and PPF-*co*-PPS lost up to 35% of their mass after 1 month in similar conditions [62], which could be attributed to their amorphous character in contrast with PEF-*co*-PES, which possesses some crystallinity. PBF-*co*-PBS with PBF contents 40–60 mol% lost only 2% of their weight after 21 weeks in PhBS [75], 1% in acidic conditions, 52% in alkaline conditions, and 90% after 180 days in compost [74]. As degradation progressed, the percentage of crystallinity of the polymers increased. PBF-*co*-PBS was also found compostable according to ISO 14855-1:2005 for furan molar contents 5%, 10%, and 20% [82]. Similar results were obtained for PBF-*co*-PBS copolymers prepared by ROP in different hydrolysis conditions, with mass loss up to 17.5% for succinate content 60 mol% in the presence of enzymes [83]. Increasing the succinate content also led to accelerated mass loss rates. Finally, PNF-*co*-PNS copolymers had a slow but still present weight loss in both PhBS and PhBS/lipase solutions [102]. The slow rate was attributed to the steric hindrance caused by neopentyl glycol and cold crystallization at 37 °C.

Adipic acid is one of the most attractive biobased monomers that yields biodegradable polyesters, since one of the most well-known biodegradable commercial copolymers is PBAd-*co*-PBT, which possesses a balance between its physical properties and its biodegradation rate [72]. Consequently, some of the first efforts to prepare biodegradable and biobased copolymers with FDCA included adipic acid as a comonomer. Both PEF and PBF have been combined with PEAd and PBAd respectively, in an effort to determine the biodegradability in relation to the physical and mechanical properties [50,72,74,75]. In the study of Papadopoulos et al., the enzymatic hydrolysis rate of PEF-*co*-PEAd copolymers was affected more by the comonomer content, rather than crystallinity or Mw [50]. PBF-*co*-PBAd with BF content <75 mol% was hydrolyzable by enzymes [72]. PBF-*co*-PBAd with 10 mol% BF degraded faster than PBAd due to its smaller melting temperature. In the absence of enzymes, they only lost approximately 5% of their initial weight after 22 weeks [75]. During this time, the intrinsic viscosity of all copolymers decreased exponentially, and the degradation rate slowed down with increasing both the BF content and percentage of crystallinity. To extend this study, the authors performed hydrolysis experiments on PBF-*co*-PBAd with BF contents 40–60 mol% in different pH values and in composting conditions [74]. Under alkaline conditions, degradation was greatly accelerated, while all copolyesters were found compostable according to ISO 14855-1:2005 and GB/T 19277.2-2013 and degraded faster than their TPA-based counterparts. Besides high adipate content, crystallinity was believed to be beneficial to microorganism adhesion and erosion, resulting in faster degradation rates. 

PCL is a polyester that undergoes rapid degradation under the influence of enzymes. Biodegradable PBF-*co*-PCL elastomer copolymers have been reported recently, and their enzymatic degradation was evaluated [85,86]. Hu et al. prepared the copolymers from DMFD with final molecular weights 6790–11,250 g/mol, that exhibited up to 20% weight loss after 40 days of enzymatic hydrolysis [85], and Morales-Huerta et al. prepared them from cyclic oligo-BF with Mw = 22,000–50,000 g/mol with weight loss of about 55% after 40 days [86]. Even if the two studies concerned copolymers with the same structure with differences only on their Mw and sequence distribution, hydrolyzed by the same enzyme, the lack of a consistent, standardized process for the evaluation of enzymatic hydrolysis rates does not allow the immediate comparison of the results of the two studies. That is because different concentrations of the enzyme were used, preventing the readers from ascertaining which properties led to the different hydrolysis rates. However, it is clear that upon increasing the molar content of CL units, the hydrolysis rate increases. PCL has also been introduced to PPeF and PHF to provide them with biodegradability [95]. Indeed, both series of copolymers showed accelerated mass loss in comparison with the furanic homopolyesters. PHF-*co*-PCL showed faster hydrolysis than PPeF-*co*-PCL. PHF-*co*-PCL had lower [η] values than PPeF-*co*-PCL, smaller *T*_g_ and larger crystallinity, highlighting the important role of the *T*_g_ on degradation rates.

PBF-*co*-PBC copolymers hydrolyzed under the influence of enzymes in PBC contents 40–70 mol% because of the susceptibility of the carbonate units to biodegradation [84]. PDF-*co*-PIsF copolymers lost a significant amount of weight in garden soil; however, this was not enough to be considered biodegradable according to ISO 14855-1:2005 [100]. However, this small biodegradation is important, as these copolymers do not contain aliphatic dicarboxylic acids, but both repeating units have a furan ring. Similarly, PImF showed up to 50% mass loss during enzymatic hydrolysis, and the corresponding PBF-PImF copolymers were also biodegradable [66]. Poly(ester carbonate)s PIsF-PBF-PIC-PBS with high molecular weights were noticeably degradable in comparison with PBF, and that biodegradation originated from the butylene succinate and the butylene carbonate units [70]. 

After reviewing the reports on biodegradation of the copolymers, it is obvious that most studies indicate that the chemical structure of the comonomers and its consequent hydrophilicity are the main factors that affect the hydrolysis rate. Secondary parameters include *T*_g_, crystallinity, and Mw. The use of a common, standardized biodegradation experimental procedure would help scientists’ study in more detail the effect of different physicochemical properties on the biodegradation rates of polyesters, making their tuning easier.

## 8. Potential Applications 

The main applications of FDCA-based polyesters are believed to be associated with packaging. PEF is anticipated to replace PET in the manufacturing of bottles, films, and consumer goods. Currently, the industry is focusing on the development of transparent PEF bottles with a sustainable cost. PPF has the potential of finding applications in both packaging, due to its extraordinary barrier properties, as well as in fibers, similarly to its terephthalic counterpart PPT. When the problems of monomer purity and cost will be fully addressed, the commercialization of these polymers is expected to grow fast, and versatile applications will be explored in the continuous effort to reach a sustainable economy. Similar to conventional plastics, the properties of FDCA-based polyesters will need tuning depending on the application, and the already published research is going to accelerate that process. 

Many authors orient their studies having in mind the final product and its desired properties. Copolyesters with FDCA are designed in a way to be able to be used as bottles [32], food packaging transparent films [61,62,63], biodegradable films [62,82], fibers [43,48], self-healing or shape-memory materials [47,85,103], thermoplastic elastomers [56,58,86,87,94,101], impact modifiers [79], conductive films [31], polyester binders as precursors for polyurethane coatings [129], and degradation-accelerating fillers in biodegradable polymers [62]. Specialty food packaging is likely to have a leading role due to the excellent gas barrier properties of FDCA polyesters, which can successfully be imparted with biodegradability while maintaining their biobased character, their mechanical properties, and their transparency. The biodegradable copolymers could also find applications on tissue engineering, as long as they remain biocompatible, but further studies are required to explore that path [90].

PEF-*co*-PET copolymers could be used to replace PEI-*co*-PET copolymers in packaging applications, since they could give transparent films with improved barrier properties [40,44,129]. The approach of copolymerizing PEF with PET is also an alternative method of improving the barrier properties of PET with using a biobased copolymer, instead of the usual polyamide. Avantium also reported that blending PET with PEF yields more transparent and less permeable bottles than blending with polyamide [130]. The role of isophthalic acid that FDCA is substituting is reducing the crystallinity of PET. PEF-*co*-PET was also successfully spun into fibers by melt-spinning and hot-drawing with similar or better tenacity to PET fibers [43]. The copolymerization of FDCA with cyclic diols can give biobased alternatives to amorphous, durable and tough petrochemical-based copolymers such as Tritan™, which is a PET copolymer with CBDO that is suitable for heat-resistant drink bottles [30,32,34,35,36,65,100]. 

A plethora of biodegradable and fully biobased copolymers has been explored, as discussed in Section 7, providing with a multitude of different, tunable properties that depend on the comonomer composition. When mechanical and thermal properties are adequate, the copolymers could be used as biodegradable and biobased heat-resistant and strong items such as containers [70,84,97,102]. Some are discussed as the biobased alternatives of PBAd-*co*-PBT, which also possess better barrier and mechanical properties, such as PBF-*co*-PGA [92]. Polyactive™ is a biodegradable PET-*co*-PEO copolymer used for drug delivery and tissue engineering that could potentially be replaced by PBF-*co*-PEG copolymers [90,91]. PBAd-*co*-PBT, known as Ecoflex^®^, can be replaced by PBF-*co*-PBAd or PBF-*co*-PBS copolymers [72,74,75]. 

FDCA copolymers with *O*-acetylvanillic acid were used as components of a thermotropic polyesters to reduce its melting point, as replacements for the commonly used petroleum-derived flexible spacers [104]. 

## 9. Concluding Remarks 

Sustainability has become an integral part of polymer science and will remain in the forefront of research and development of new biobased plastics. Polymers derived from FDCA are expected to play a leading role in the following years as part of the bioeconomy initiative that is promoted nearly worldwide. To ensure the reduction in the use of fossil-based plastics and the accumulation of their waste, authorities have to support research organizations and industries both financially but also with legislation and by educating the public, while keeping in mind the ultimate goal of stabilizing the atmospheric greenhouse gas levels and putting a halt on global climate change. The dominance of biobased polymers will depend heavily on the advances on lignin valorization and isolation of high-purity monomers that will allow the production of cheap, colorless plastics. 

After reviewing the available literature, copolymerization is clearly a valuable method for the tuning of the properties of FDCA-based polyesters. They can be comparable or even better than commercial, fossil-based polymers in terms of physicochemical properties. This will allow their use in diverse applications that can extend further from specialty packaging. Simultaneously, copolymerization helps with overcoming some of the problems that are related with FDCA, such as coloration, high cost, and a lack of biodegradation. Some aspects that must not be overlooked are the methods of waste disposal of bioplastics, their effect on microplastic formation in the oceans, and life cycle assessments. Biodegradation studies that simulate the environment only concern soil, but as the severity of the accumulation of polymeric microplastics in water masses is recognized, the degradation of new biobased polymers in aquatic environments will have to be evaluated, too. In this direction, new testing standards need to be implemented. 

Several research groups from all over the world have published valuable data on a plethora of copolyesters that are synthesized in the typical polyester synthesis infrastructures. Hopefully, this work will help accomplish the dream of a sustainable future that will require a close collaboration of industries, scientists, and governments. 

## Figures and Tables

**Table 1:** Copolyesters with 2,5-furandicarboxylic acid (FDCA) reported in the literature. Each polymer’s abbreviation is defined in the “repeating units” columns.

Copolymer	Repeating Units (Abbrev.)		Structure	Reference
**PEF with comonomers containing cyclic units**				
poly(ethylene 2,5-furandicarboxylate-*co*-ethylene 2,4-furan dicarboxylate) (PEF-*co*-2,4 PEF)	2,5-PEF	2,4-PEF	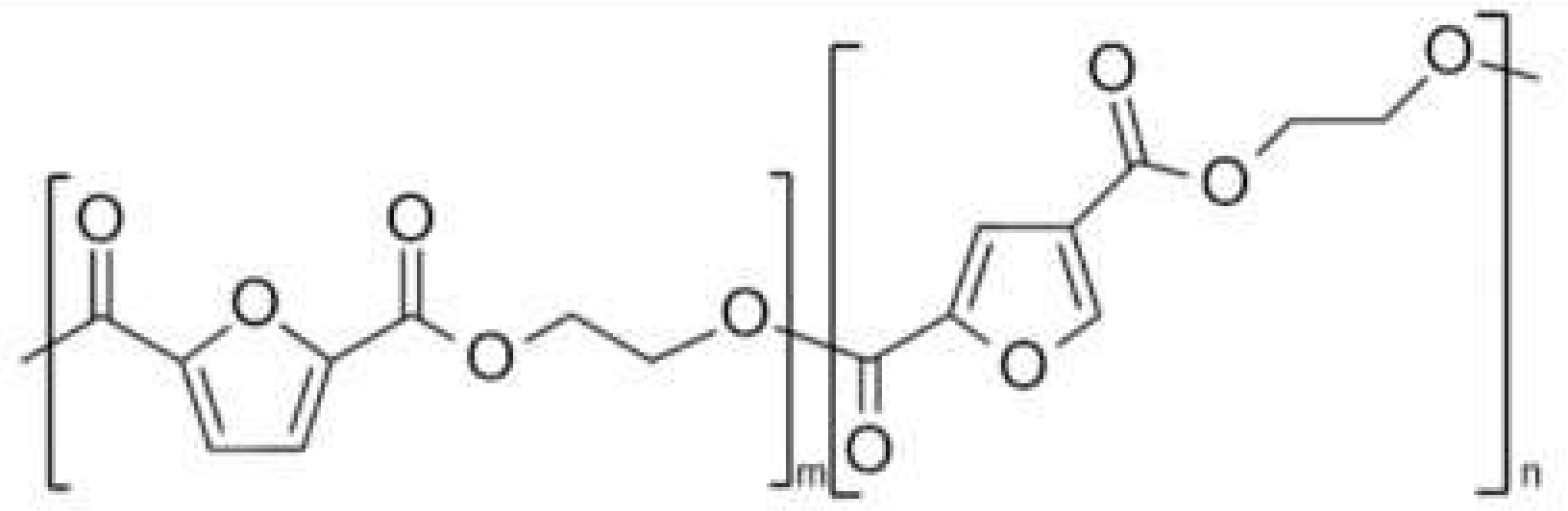	[28]
poly(ethylene 2,5-furandicarboxylate)-*co*-butylene 2,5-furandicarboxylate) (PEF-*co*-PBF)	PEF	PBF	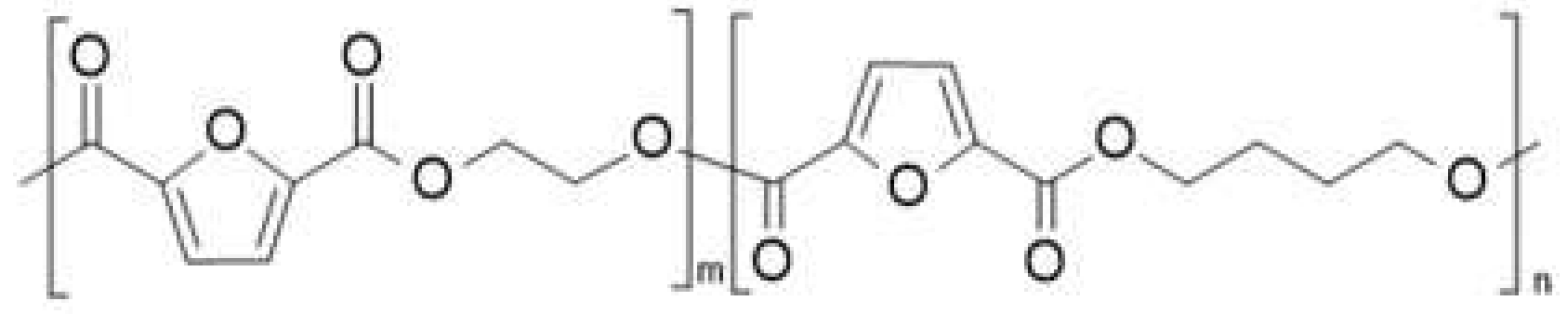	[29]
poly(ethylene 2,5-furandicarboxylate-*co*-1,4-cyclohexanedimethylene 2,5-furandicarboxylate) (PEF-*co*-PCHDMF)	PEF	PCHDMF	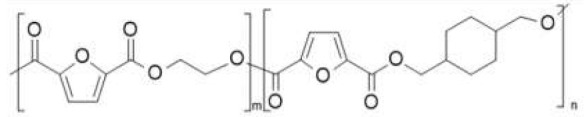	[30,31,32]
poly(ethylene 2,5-furandicarboxylate-*co*-ethylene 1,4-cyclohexanedicarboxylate) (PEF-*co*-PECH)	PEF	PECH	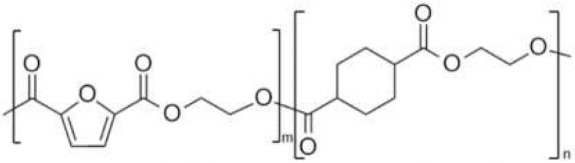	[33]
poly(ethylene 2,5-furandicarboxylate-*co-*2,2,4,4-tetramethyl-1,3-cyclobutanediol 2,5-furan dicarboxylate) (PEF-*co*-PCBDOF)	PEF	PCBDOF	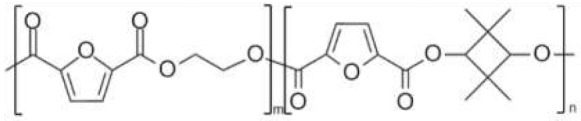	[34,35]
poly(ethylene 2,5-furandicarboxylate-*co*-1,4-cyclohexyldimethylene 2,5-furandicarboxylate-*co*-2,2,4,4-tetramethyl-1,3-cyclobutanediol 2,5-furandicarboxylate) (PEF-*co*-PCHDMF-*co*-PCBDOF)	PEF	PCHDMF PCBDOF	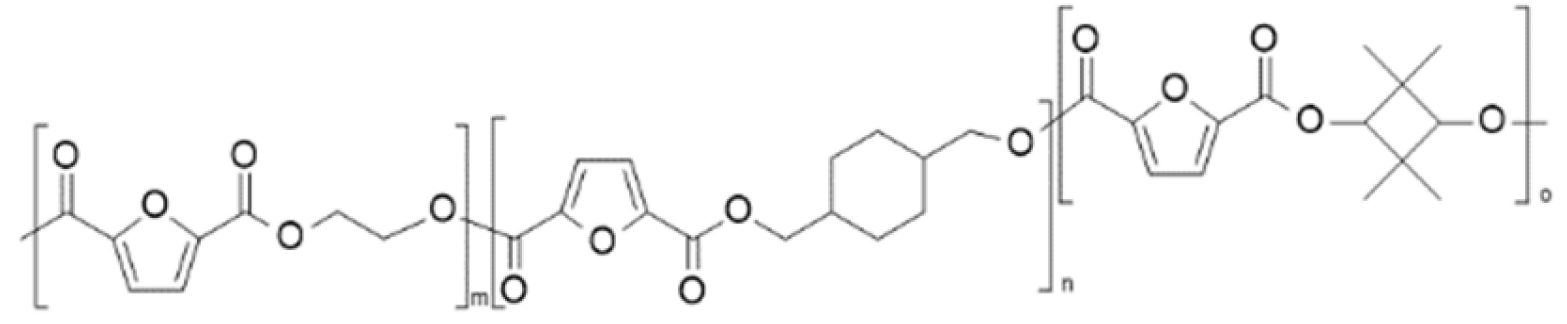	[36]
poly(ethylene 2,5-furandicarboxylate-*co*-pentylene 2,5-furandicarboxylate) (PEF-*co*-PPeF)	PEF	PPeF	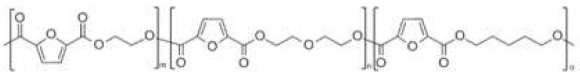	[37]
poly(ethylene 2,5-furan dicarboxylate-*co*-ethylene terephthalate) (PEF-*co*-PET)	PEF	PET	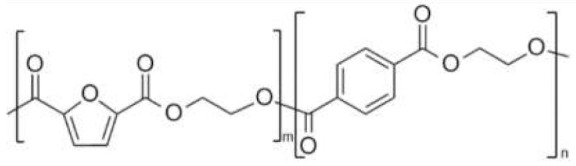	[38,39,40,41,42,43,44]
poly(ethylene 2,5-furan dicarboxylate-*co*-hexamethylene 2,5-furandicarboxylate) (PEF-*co*-PHF)	PEF	PHF	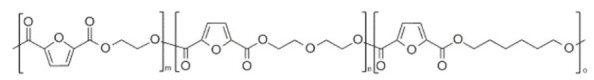	[45]
poly((poly(ethylene glycol)) 2,5-furandicarboxylate)-*co*-poly(isosorbide 2,5-furandicarboxylate) (PPEGF-*co*-PIsF)	PEGF	PIsF	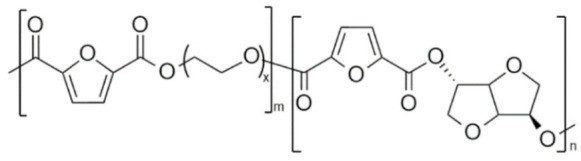	[46]
poly(ethylene 2,5-furan dicarboxylate)-*co*-poly((poly(ethylene glycol) 2,5-furandicarboxylate) (PEF-*co*-PPEGF)	PEF	PPEGF	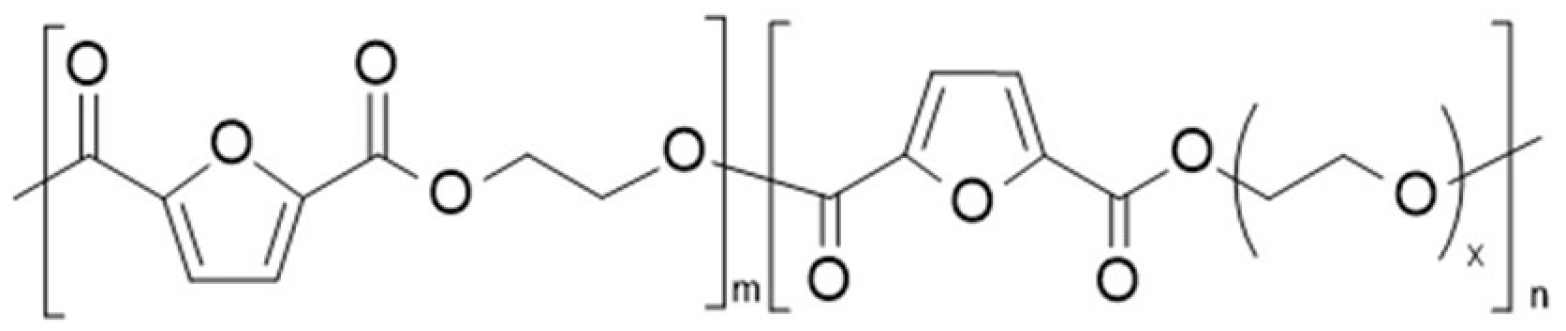	[47,48]
poly(ethylene 2,5-furandicarboxylate-*co*-*ε*-caprolactone) (PEF-*co*-PCL)	PEF	PCL	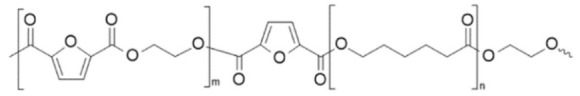	[49]
poly(ethylene 2,5-furandicarboxylate-*co*-ethylene adipate) (PEF-*co*-PEAd)	PEF	PEAd	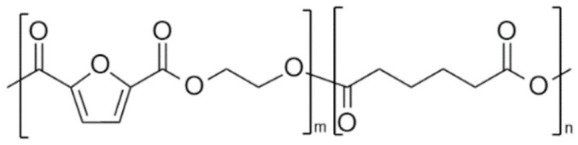	[50]
poly(ethylene 2,5-furan dicarboxylate-*co*-ethylene sebacate) (PEF-*co*-PESeb)	PEF	PESeb	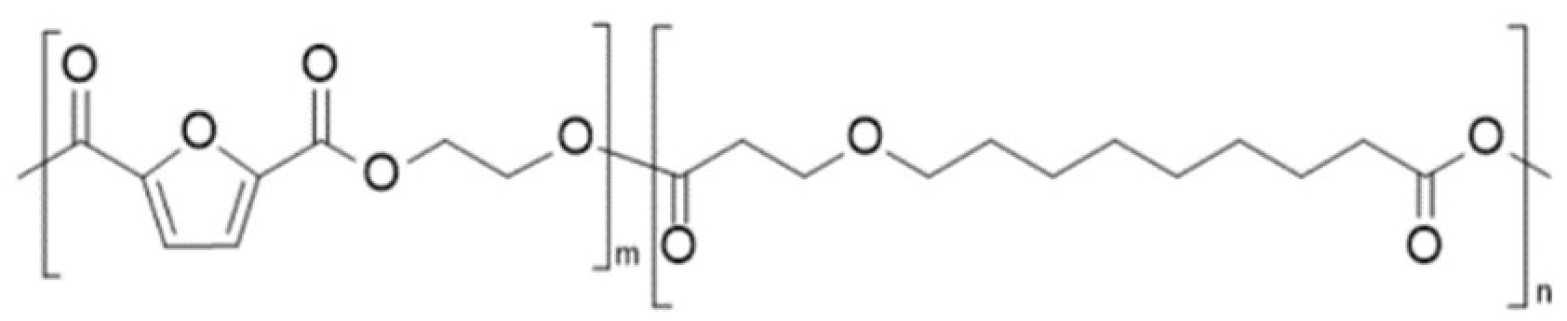	[51]
poly(ethylene 2,5-furan dicarboxylate-*co*-ethylene succinate) (PEF-*co*-PES)	PEF	PES	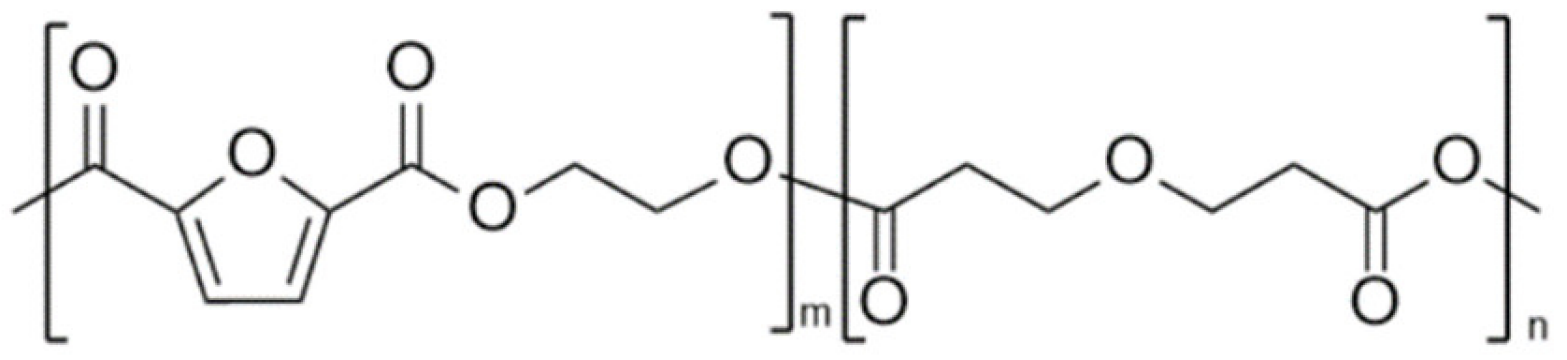	[52,53]
Poly(ethylene 2,5-furandicarboxylate-*co*-lactic acid) (PEF-*co*-PLA)	PEF	PLA	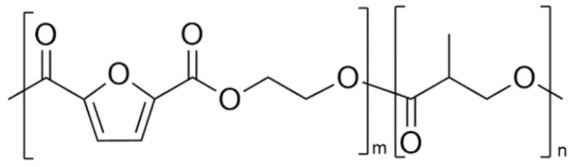	[54,55]
Poly(ethylene 2,5-furandicarboxylate-mb-poly(tetramethylene glycol)) (PEF-*co*-PPTMGF)	PEF	PPTMGF	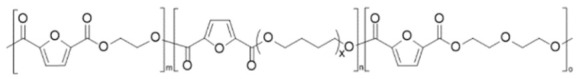	[56]
**PPF with comonomers containing cyclic units**				
Poly(propylene 2,5-furandicarboxylate-*co*-propylene 2,4-furan dicarboxylate) (PPF-*co*-2,4 PPF)	2,5-PPF	2,4-PPF	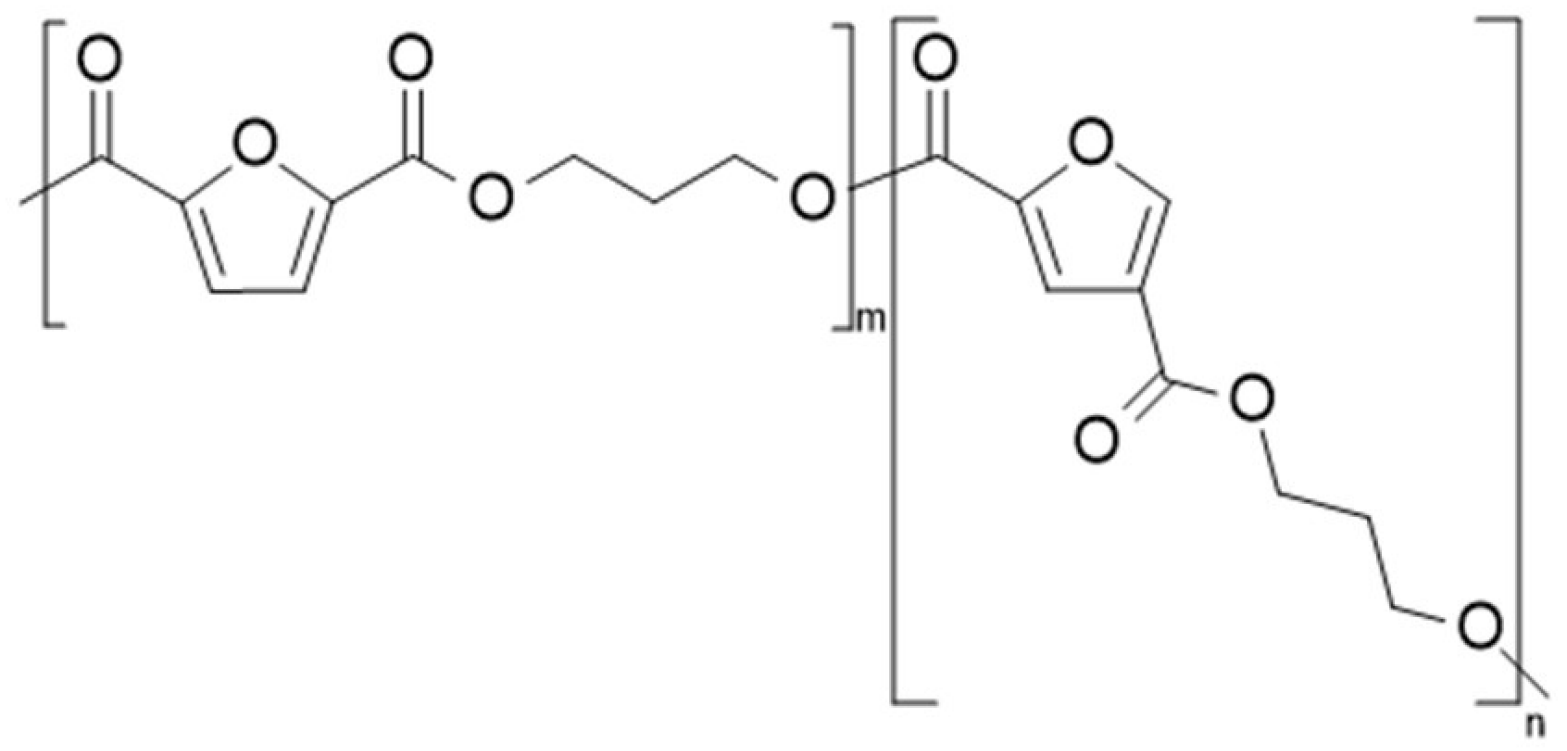	[28]
Poly(propylene 2,5-furandicarboxylate-*b*-dimerized fatty acid diol) (PPF-*b*-PFADDF)	PPF	FADD	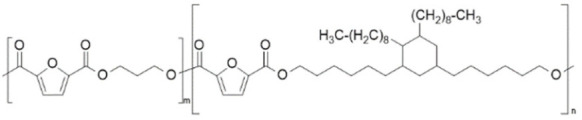	[57,58]
poly(propylene 2,5-furandicarboxylate-*co*-1,4-cyclohexanedimethylene 2,5-furandicarboxylate) (PPF-*co*-PCHDMF)	PPF	PCHDMF	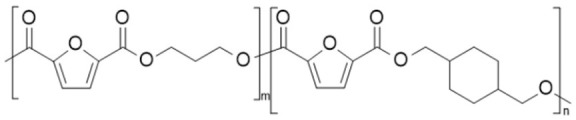	[59]
poly(propylene 2,5-furandicarboxylate-*co*-2,2,4,4-tetramethyl-1,3-cyclobutanediol 2,5-furandicarboxylate) (PPF-*co*-PCBDOF)	PPF	PCBDOF	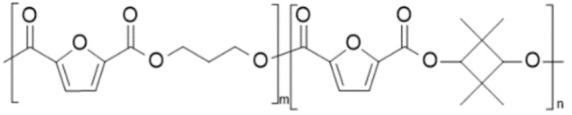	[34]
poly(propylene 2,5-furandicarboxylate-*co*-2-methyl-1,3-propanediol 2,5-furandicarboxylate) (PPF-*co*-PMePF)	PPF	PMePF	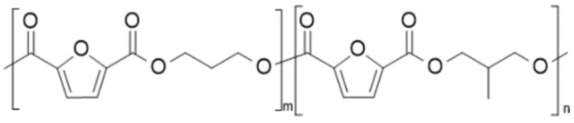	[60]
poly(propylene 2,5-furandicarboxylate-*co*-propylene cyclohexane dicarboxylate) (PPF-*co*-PPCH)	PPF	PPCH	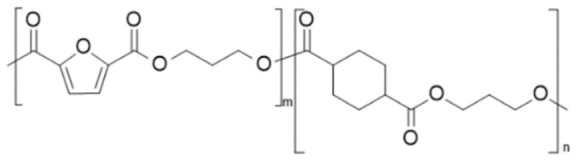	[61]
poly(propylene 2,5-furan dicarboxylate-*co*-propylene terephthalate) (PPF-*co*-PPT)	PPF	PPT	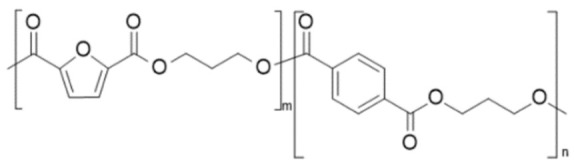	[42]
**PPF with comonomers containing linear units**				
poly(propylene 2,5-furandicarboxylate-*co*-succinate) (PPF-*co*-PPS)	PPF	PPS	** 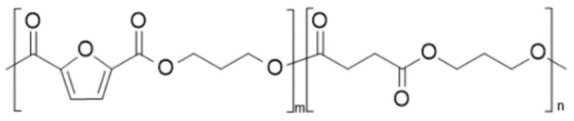 **	[62]
**PBF with comonomers containing cyclic units**				
Poly(butylene 2,5-furandicarboxylate-*co*-butylene 2,4-furan dicarboxylate) (PBF-*co*-2,4 PBF)	2,5-PBF	2,4-PBF	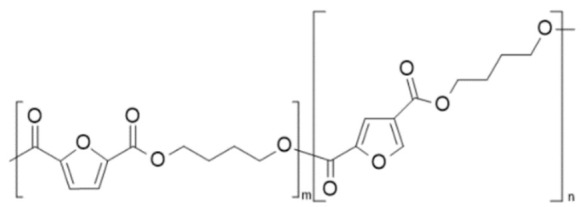	[28]
Poly(butylene 2,5-furan dicarboxylate-*co*-butylene bis-2,5-furan dicarboxylate) (PBF-*co*-PBbF)	PBF	PBbF	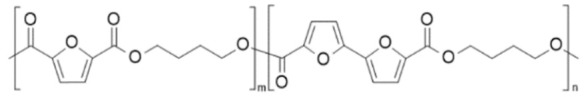	[63]
poly(butylene 2,5-furandicarboxylate-*co*-butylene terephthalate) (PBF-*co*-PBT)	PBF	PBT	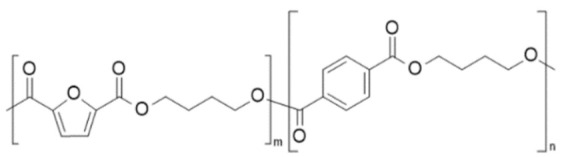	[42,64]
poly(butylene 2,5-furandicarboxylate-*co*-2,2,4,4-tetramethyl-1,3-cyclobutanediol 2,5-furandicarboxylate) (PBF-*co*-PCBDOF)	PBF	PCBDOF	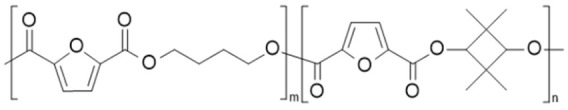	[34]
poly(butylene 2,5-furandicarboxylate-*co*-1,4-cyclohexanedimethylene 2,5-furandicarboxylate) (PBF-*co*-PCHDMF)	PBF	PCDHDMF	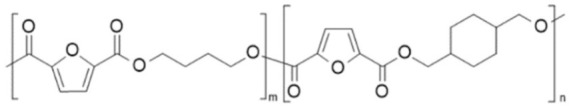	[65]
poly(butylene 2,5-furandicarboxylate-*co*-isomannide 2,5-furandicarboxylate) (PBF-*co*-PImF)	PBF	PImF	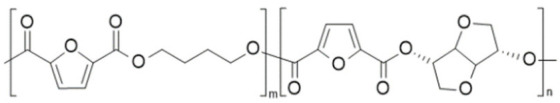	[66]
poly(butylene 2,5-furandicarboxylate-*co*-isosorbide 2,5-furandicarboxylate) (PBF-*co*-PIsF)	PBF	PIsF	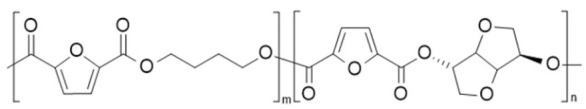	[67,68]
poly(butylene 2,5-furandicarboxylate-*co*-butylene isophthalate) (PBF-*co*-PBI)	PBF	PBI	** 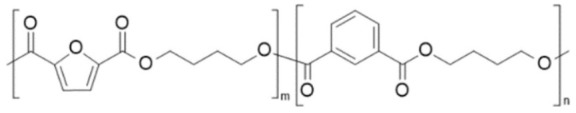 **	[69]
poly(butylene 2,5-furandicarboxylate-*co*-butylene succinate-*co*-isosorbide carbonate) (PBF-*co*-PIsC-*co*-PBS)	PBF	PBSu/PIsC	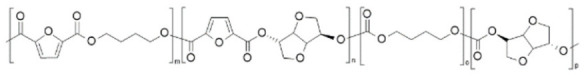	[70]
poly(butylene 2,5-furandicarboxylate-*co*-propylene 2,5-furandicarboxylate) (PBF-*co*-PPF)	PBF	PPF	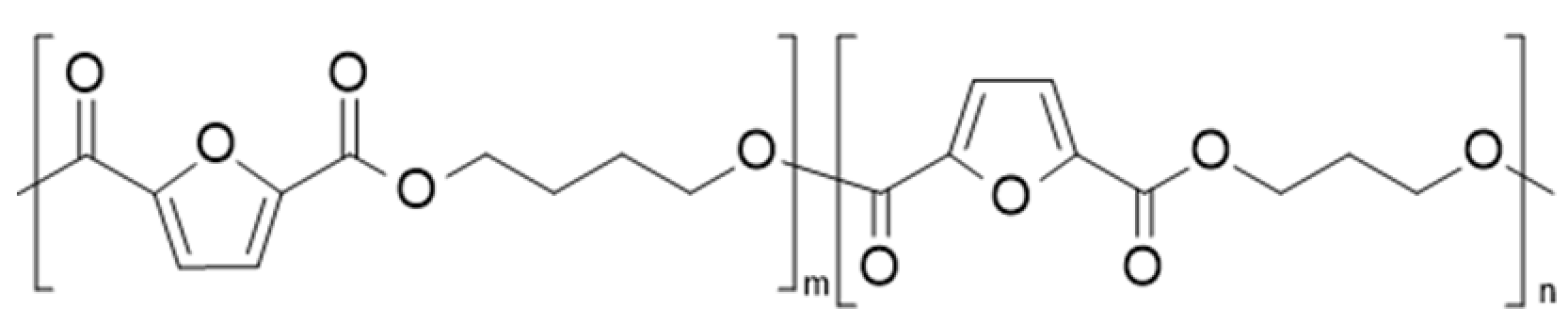	[71]
**PBF with comonomers containing linear units**				
poly(butylene 2,5-furan dicarboxylate-*co*-butylene adipate) (PBF-*co*-PBAd)	PBF	PBAd	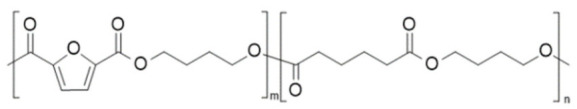	[72,73,74,75]
poly(butylene 2,5-furan dicarboxylate-*co*-butylene diglycolate) (PBF-*co*-PBdGA)	PBF	PBdGA	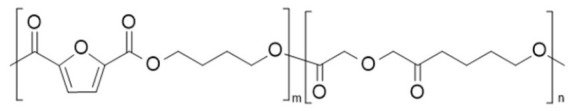	[76,77]
poly(butylene 2,5-furan dicarboxylate-*co*-butylene sebacate) (PBF-*co*-PBSeb)	PBF	PBSeb	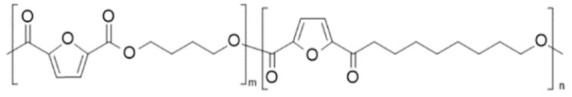	[78]
poly(butylene 2,5-furan dicarboxylate-*co*-butylene succinate) (PBF-*co*-PBS)	PBF	PBS	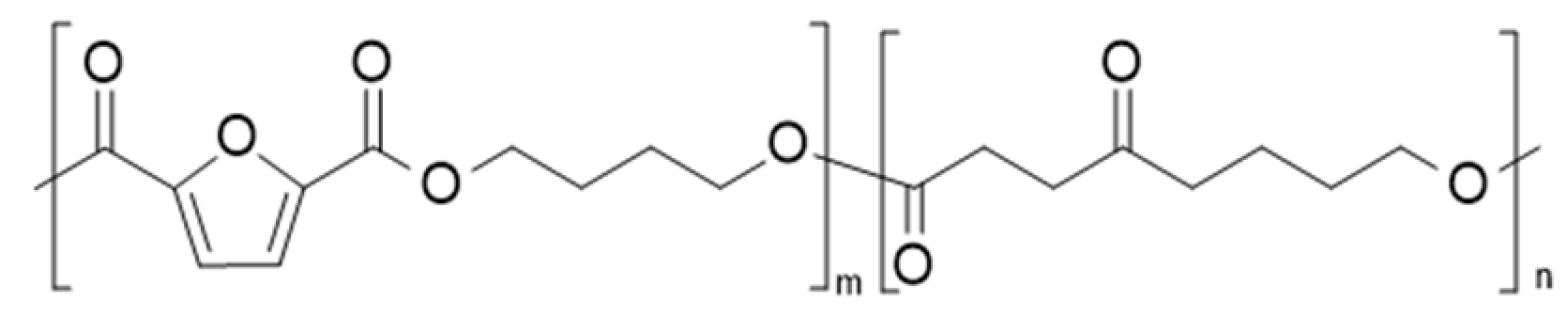	[74,75,79,80,81,82,83]
poly(butylene 2,5-furan dicarboxylate-*co*-butylene carbonate) (PBF-*co*-PBC)	PBF	PBC	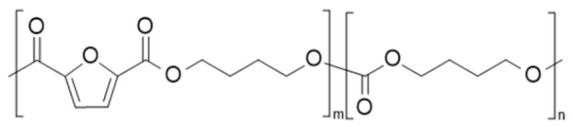	[84]
poly(butylene 2,5-furan dicarboxylate-*co*-*ε*-caprolactone) (PBF-*co*-PCL)	PBF	PCL	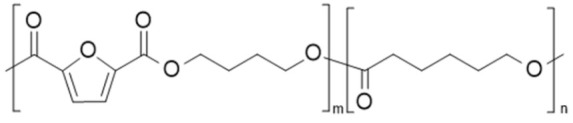	[85,86,87]
poly(butylene 2,5-furandicarboxylate)-*b*-poly(ethylene glycol) 2,5-furan dicarboxylate) (PBF-*b*-PEG)	PBF	PPEGF	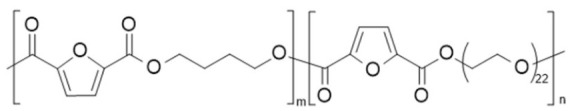	[88,89,90,91]
poly(butylene 2,5-furan dicarboxylate-*co*-glycolate) (PBF-*co*-PGA)	PBF	PGA	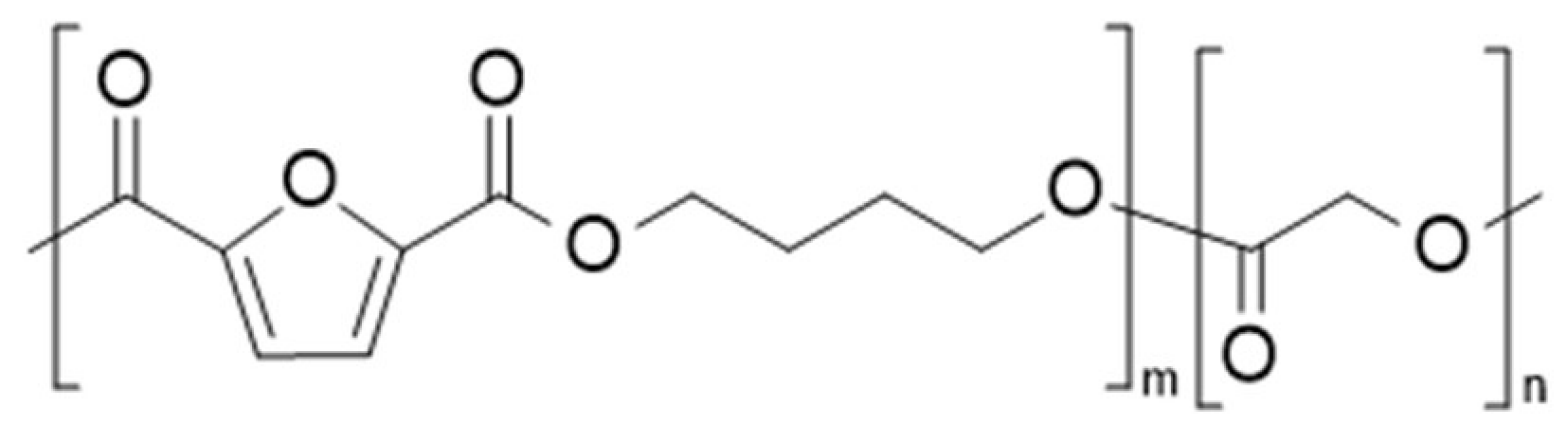	[92]
poly(butylene 2,5-furan dicarboxylate-*co*-poly(propylene oxide) 2,5-furan dicarboxylate) (PBF-*co*-PPPOF)	PBF	PPPOF	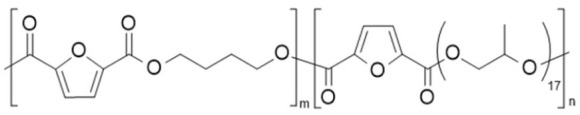	[93]
poly(butylene 2,5-furan dicarboxylate)-*b*-poly(tetramethylene glycol) 2,5-furan dicarboxylate) (PBF-*co*-PPTMGF)	PBF	PPTMGF	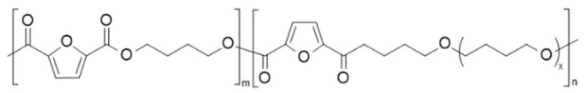	[94]
**PPeF copolymers**				
poly(pentylene 2,5-furandicarboxylate-*co*-*ε*-caprolactone) (PPeF-*co*-PCL)	PPeF	PCL	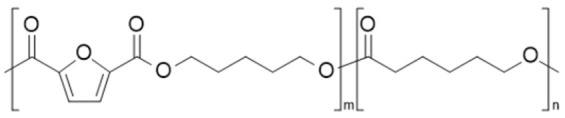	[95]
**PHF copolymers**				
poly(hexamethylene 2,5-furandicarboxylate-*co*-hexamethylene 2-carboxyethyl (phenyl) phosphinic acid) (PHF-*co*-PHPCEPPA)	PHF	PCEPPA	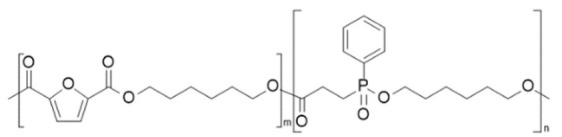	[96]
poly(hexamethylene 2,5-furandicarboxylate-*co*-caprolactone) (PHF-*co*-PCL)	PHF	PCL	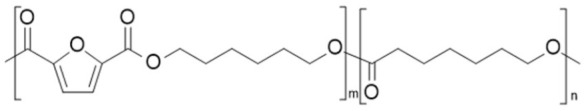	[95]
poly(hexylene 2,5-furandicarboxylate-*co*-hexylene terephthalate) (PHF-*co*-PHT)	PHF	PHT	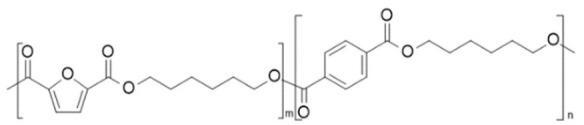	[42]
poly(hexylene 2,5-furan dicarboxylate-*co*-isosorbide-2,5-furan dicarboxylate) (PHF-*co*-PIsF)	PHF	PIsF	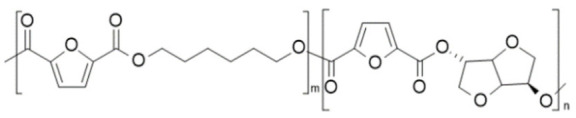	[97]
poly(lactic acid-*b*-hexylene 2,5-furan dicarboxylate-*b*-lactic acid) (PHF-*b*-PLA)	PHF	PLA	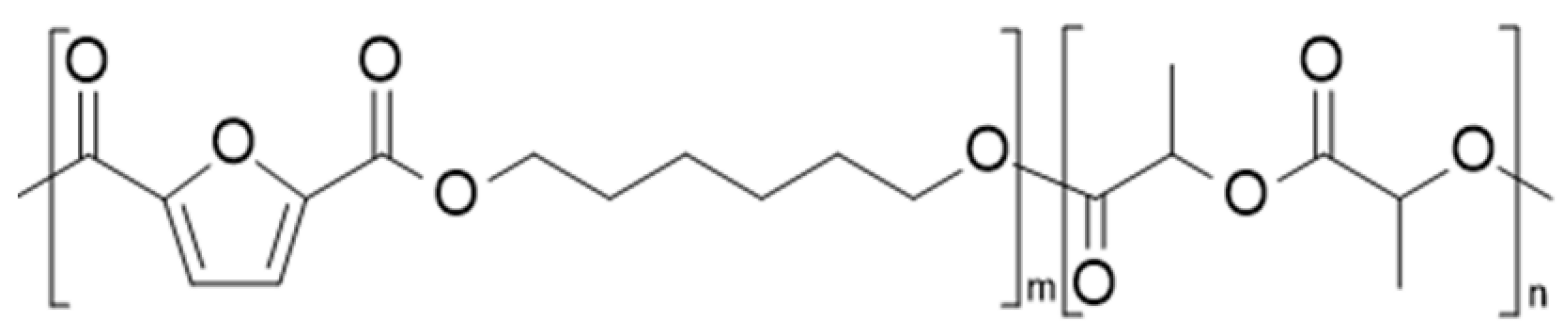	[98]
**Other furan-based copolymers**				
poly(1,4-cyclohexanedimethanol-*co*-isosorbide 2,5-furandicarboxylate) (PCHDMF-*co*-PIsF)	PCHDMF	PIsF	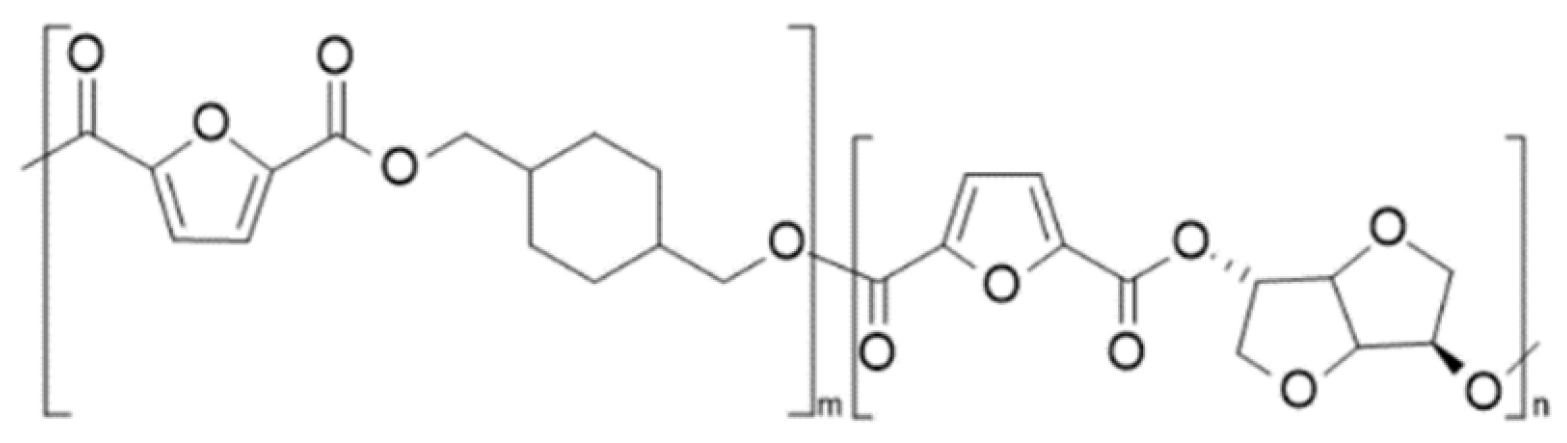	[99]
poly(decamethylene 2,5-furan dicarboxylate-*co*-isosorbide-2,5-furan dicarboxylate) (PDF-*co*-PIsF)	PDF	PIsF	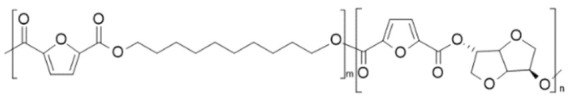	[100]
poly(neopentyl glycol 2,5-furandicarboxylate-*co*-poly(tetramethylene glycol) 2,5-furan dicarboxylate) (PNF-*co*-PPTMGF)	PNF	PPTMGF	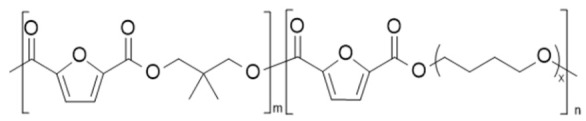	[101]
poly(neopentyl glycol 2,5-furandicarboxylate-*co*-neopentyl glycol succinate) (PNF-*co*-PNS)	PNF	PNGS	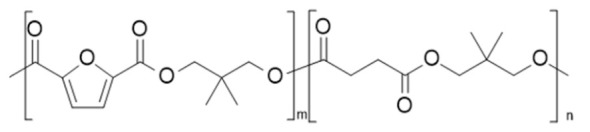	[102]
poly(isosorbide 2,5-furandicarboxylate-*co*-*ε*-caprolactone) (PIsF-*co*-PCL)	PIsF	PCL	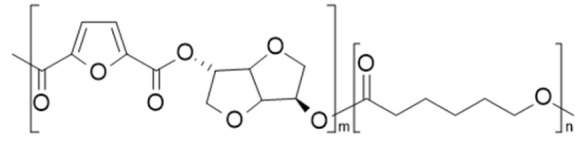	[67]
poly(2,5-furan dimethylene succinate-*co*-propylene succinate) (PFDMS-*co*-PPS)	PFDMS	PPS	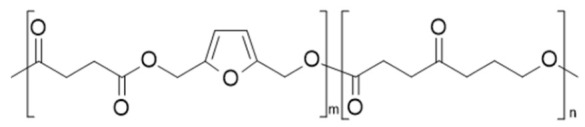	[103]
poly(octylene 2,5-furandicarboxylate-*co*-octylene terephthalate) (POF-*co*-POT)	POF	POT	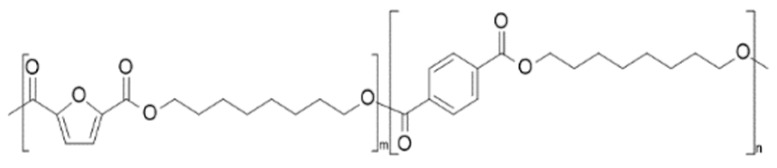	[42]
poly(p-acetobenzoic acid-*co*-4,4′-diacetoxybiphenyl 2,5-furan dicarboxylate) (PAA-*co*-PDABPHF)	PAA	PDABPHF	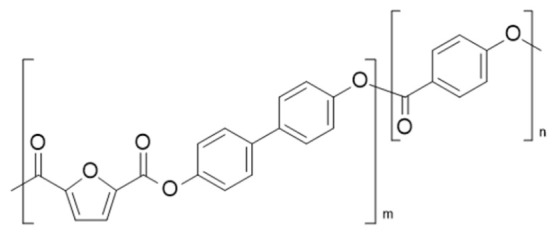	[104]
poly(di- * O * -2-(hydroxyethyl) resorcinol 2,5-furandicarboxylate-*co*-ethylene succinate)€(PRF-*co*-PES)	PRF	PES	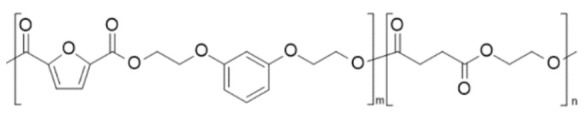	[105]
poly(di- * O * -2-(hydroxyethyl) resorcinol 2,5-furandicarboxylate-*co*-butylene succinate)€(PRF-*co*-PBS)	PRF	PBS	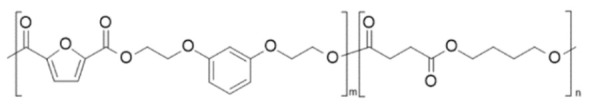	[105]

**Table 2:** Influence of the isosorbide carbonate and butylene succinate units in the tensile properties of PBF-*co*-PBS-*co*-PIsC copolymers [68,70].

Sample	Tensile Strength (*σ*_b_)	Young’s Modulus (*E*)	Elongation (*ε*_b_)	[η]
	MPa	MPa	%	dL/g
PIs80B20F	77 ± 3	1900 ± 60	15 ± 3	0.45
PIs80CBF20	29.7 ± 2.5	1117 ± 28	3 ± 1	0.50
PIs80CBF10S10	69 ± 8.1	1330 ± 38	7 ± 1	0.77
PIs60B40F	134 ± 3	1590 ± 42	32 ± 2	0.58
PIs60CBF40	40.3 ± 1.5	1186 ± 30	5 ± 1	0.58
PIs60CBF30S10	71.5 ± 2.2	1242 ± 31	23 ± 7	0.82

## Abbreviations

BDO	1,4-butanediol
CALB	*Candida arctica* lipase B
CBDO	2,2,4,4-tetramethyl-1,3-cyclobutanediol
CH	1,4-cyclohexane dicarboxylic acid
CHDM	1,4-Cyclohexanedimethanol
CL	ε-caprolactone
DMA	Dynamic Mechanical Analysis
DMFD	dimethyl furan dicarboxylate
DSC	Diffential Scanning Calorimetry
*E*	Young’s modulus
E’	Storage Modulus
EC	European Commision
EF	ethylene furanoate
EG	ethylene glycol
FADD	dimerized fatty acid diol
FDCA	furan dicarboxylic acid
FSC	Fast Scanning Calorimetry
HDO	1,6-hexanediol
Mw	molecular weight
PAA	poly(p-acetobenzoic acid)
PBAd	poly(butylene adipate)
PBbF	poly(butylene bis-2,5-furan dicarboxylate)
PBC	poly(butylene carbonate)
PBdGA	poly(Butylene Diglycolate)
PBF	poly(butylene 2,5-furan dicarboxylate)
PBI	poly(butylene isophthalate)
PBS	poly(butylene succinate)
PBSeb	poly(butylene sebacate)
PBT	poly(butylene terepthalate)
PC	polycarbonate
PCBDOF	poly(2,2,4,4-tetramethyl-1,3-cyclobutanediol 2,5-furan dicarboxylate)
PCHDMF	poly(1,4-cyclohexanedimethylene 2,5-furandicarboxylate)
PCL	poly(ε-caprolactone)
PDABPHF	poly(4,4′-diacetoxybiphenyl 2,5-furan dicarboxylate)
PDF	poly(decylene 2,5-furan dicarboxylate)
PDO	1,3-propanediol
PDoF	poly(dodecylene 2,5-furan dicarboxylate)
PEAd	poly(ethylene adipate)
PECH	poly(ethylene 1,4-cyclohexanedicarboxylate)
PeDO	1,5-pentanediol
PEF	poly(ethylene 2,5-furan dicarboxylate)
PEG	poly(ethylene glycol)
PEI	poly(ethylene isophthalate)
PES	poly(ethylene succinate)
PESeb	poly(ethylene sebacate)
PET	poly(ethylene terepthlalate)
PFDMS	poly(2,5-furandimethylene succinate)
PGA	poly(glycolic acid)
PhBS	phosphate buffer solution
PHCEPPA	poly(hexamethylene 2-carboxyethyl (phenyl) phosphinic acid)
PHF	poly(hexylene 2,5-furan dicarboxylate)
PHT	poly(hexylene terepthalate)
PIC	poly(isosorbide carbonate)
PImF	poly(isomannide furandicarboxylate)
PIsF	poly(isosorbide 2,5-furandicarboxylate)
PLA	poly(lactic acid)
PMePF	poly(2-methyl-1,3-propanediol 2,5-furandicarboxylate)
PNF	poly(neopentyl glycol 2,5-furandicarboxylate)
PNoF	poly(nonylene 2,5-furan dicarboxylate)
POF	poly(octylene 2,5-furan dicarboxylate)
POT	poly(octylene terepthlalate)
PPCH	poly(propylene cyclohexane dicarboxylate)
PPeF	poly(pentylene 2,5-furan dicarboxylate)
PPEGF	poly((poly(ethylene glycol)) 2,5-furandicarboxylate)-
PPF	poly(propylene 2,5-furan dicarboxylate)
PPO	poly(propylene oxide)
PPPOF	poly(poly(propylene oxide) 2,5-furan dicarboxylate))
PPS	poly(propylene succinate)
PPT	poly(propylene terepthalate)
PPTMGF	poly(tetramethylene glycol) 2,5-furan dicarboxylate)
PRF	poly(di-*O*-2-(hydroxyethyl) resorcinol 2,5-furandicarboxylate)
PTMG	poly(tetramethylene glycol)
ROP	ring opening polymerization
Sb_2_O_3_	antimony(III) oxide
SBF	simulated body fluid
Sn(oct)_2_	Stannous octoate
TBT	titanium(IV) butoxide
T_d, 5%_	Temperature that corresponds to 5% mass loss
T_d.max_	temperature at which degradation occurs with the fastest rate
T_g_	glass transition temperature
TGA	thermogravimetric analysis
T_m_	melting temperature
TPA	terepthalic acid
Xc	% crystallinity
ε_b_	elongation at break
σ_b_	tensile stress at break
σ_γ_	tensile stress at yield

## Appendix A

**Table A1:** Mechanical properties of FDCA-based homopolymers (ND = not defined).

Polymer	Tensile Strength (*σ*_b_)	Yield Point (*σ*_γ_)	Young’s Modulus (*E*)	Elongation (*ε*_b_)	Δ*H*_m_	M_w_	[η]	Reference
	MPa	MPa	MPa	%	J/g	g/mol	dL/g	
PEF	85 ± 9	-	2800 ± 120	5 ± 1	29.6	46,900	0.82	[30,34,35]
	35 ± 8	-	2450 ± 220	2.81 ± 0.69	-	1520	ND	[131]
	56 ± 9	-	2511 ± 148	7 ± 1	24.4	ND	0.43	[33]
	25.6	-	1555	1.5	-	43,140	ND	[42]
	82 ± 5	-	3340 ± 490	4 ± 1	5.9	ND	0.82	[37,45]
	72 ± 5	-	ND	3 ± 1	-	90,300	ND	[47]
	84 ± 2	-	3430 ± 160	3 ± 1	1.2	ND	ND	[56]
	39 ± 3	-	2067 ± 212	6 ± 2	ND	ND	0.3	[49]
PPF	53 ± 2	-	2700 ± 30	50 ± 7	-	6500	0.88	[34]
	31 ± 3	-	1363 ± 158	3 ± 1	7	ND	ND	[61]
	90 ± 6	-	2460 ± 280	222 ± 20	3.21	41,300	0.74	[59]
	42	-	1055	4.2	-	56,080	0.81	[42]
	72 ± 5	-	2080 ± 100	3 ± 1	-	252,000	ND	[51]
	70.3 ± 2	-	1085.2 ± 14.6	6.3 ± 0.3	-	24,846	0.65	[60]
	98.5 ± 0.4	-	2600 ± 53	5 ± 1	-	7128	0.95	[62]
PBF	65.6 ± 2.2	61.0 ± 1.8	1360 ± 32	310 ± 15	ND	62,000	1.06	[92]
	62 ± 3	ND	2000 ± 30	290 ± 6	ND	76,000	0.98	[34]
	56.8	ND	1483	5.2	ND	44,040	ND	[42]
	58.9 ± 2.2	ND	2000 ± 100	4 ± 0.3	ND	ND	0.77	[63]
	53 ± 2	39 ± 2	1502 ± 101	685 ± 32	30.6	ND	1.23	[68]
	20.2 ± 1.7	20.1 ± 1.5	907.7 ± 42.1	184.3 ± 17.8	51.7	ND	0.81	[71]
	73.9 ± 0.9	ND	1351 ± 64	289 ± 12	27.1	ND	1.02	[89]
	35 ± 2.6	ND	875 ± 18	55 ± 10	37	ND	ND	[72,94]
	38 ± 3.1	ND	926 ± 11	90 ± 8	ND	ND	ND	[94]
	29 ± 4	34 ± 5	1283 ± 126	102 ± 51	32	ND	0.621	[77]
	66 ± 2	ND	1360 ± 32	310 ± 15	ND	62,000	ND	[70]
	55.6 ± 1.6	ND	1860 ± 160	256 ± 19	33.7	ND	ND	[73]
PPeF	14 ± 2	ND	6 ± 1	320 ± 11	-	ND	0.82	[37]
PHF	30 ± 2	ND	1830 ± 170	237 ± 33	52.7	ND	0.72	[45]
	37.4	ND	833	156.7	ND	27,260	ND	[42]
	28.6 ± 0.7	ND	ND	188 ± 22	41.2	97,400	0.90	[96]
POF	26.5 ± 1.5	23.9 ± 1.7	310.5 ± 21	160 ± 15	63.9	62,085	0.43	[132]
PNoF	21.0 ± 1.6	19.0 ± 1.4	251.7 ± 19	149 ± 11	4.3	67,284	0.50	[132]
PDF	11.4 ± 1.2	10.6 ± 1.4	201.9 ± 15	135 ± 17	64.2	57,025	0.47	[132]
PDoF	10.8 ± 0.9	9.5 ± 1.1	180.7 ± 16	130 ± 10	69.6	68,965	0.49	[132]
PCHDMF	62 ± 4	ND	2100 ± 200	18 ± 4	49.2	30,400	0.72	[30,59]
PNoF	68.1 ± 1.5	ND	1976.9 ± 30	6.0 ± 0.6	29.5	ND	0.72	[102]
	74.5 ± 2.3	ND	2315.1 ± 13	4.9 ± 0.3	39.5	ND	0.72	[102]

**Table A2:** A summary of the biodegradation studies reported on furan-based copolyesters. ND = not defined.

Copolymer	Degradation Medium	Temperature (°C)	pH	Specimen	Time (days)	Maximum Mass Loss (%)	Reference
PEF-PLA	SBF	37	6.9	Square 12–50 mg	84	60	[54]
	SBF	35	7.4	20 × 20 × 2 mm	55	25	[55]
	Garden soil	ND	ND	20 × 20 × 2 mm	55	65	
PEF-PEG	PBS	37	7.2	1 cm × 3 cm ×(0.1–0.3) mm	100	15	[48]
PBF-PEG	PBS	37	7.4	ND	35	44	[89]
	NaOH 0.01 M	37	12	ND	35	100	
	H_2_O	37	7	ND	49	27	[90]
	PBS	37	7.2-7.4	ND	49	24	
	NaOH 0.0001 M	37	10	ND	49	51	
	NaOH 0.01 M	37	12	ND	3	100	
PBF-PPOF	PBS	37	7	Square 69–113 mg	84	1.5	[93]
	PBS/porcine pancreas lipase	37	7	Square 69–113 mg	84	2.3	
PBF-PGA	PBS	37	7.4	films	35	8	[92]
	PBS/porcine pancreas lipase	37	7.4	films	35	60	
PBF-PBDG	Mautre compost	60	ND	20 × 40 × 0.2 mm	62	43	[77]
PEF-PESu	PBS/R delemar, P cepacia	50	7.2	5 × 5 × 2 mm	30	12.5	[52]
PPF-PPSu	PBS/porcine pancreas lipase	37	7.4	2 × 2 cm × 0.3 mm	28	35	[62]
PBF-PBSu	Compost (ISO 14855-1:2005)	58	ND	ND	100	120	[82]
	PBS	25	7	ND	2	154	[75]
	Sodium acetate/sodium hydrogen phosphate buffer	25	4	ND	1.4	154	[74]
	Sodium acetate/sodium hydrogen phosphate buffer	25	12	ND	14	154	
	Compost (ISO 14855-1:2005)	58		Film 20 × 20 mm	91	100	
	Citric acid buffer	37	2	Discs diameter 10mm, thickness 200 um, mass 20–30 mg	30	10	[83]
	Sodium phosphate buffer	37	7.4	Discs diameter 10mm, thickness 200 um, mass 20–30 mg	30	5	
	Sodium phosphate buffer/porcine pancreas lipase	37	7.4	Discs diameter 10mm, thickness 200 um, mass 20–30 mg	30	17.5	
PNF-PNGS	PBS	37	7.4	Film 0.5 mm	70	7	[102]
	PBS/*Candida antarctica* lipase B	37	7.4	Film 0.5 mm	70	12	
PEF-PEAd	PBS/R oryzae, P cepacia	37	7.2	5 × 5 × 0.4 mm	30	100	[50]
PBF-PBAd	PBS/lipase from porcine pancreas	37	7.2	10 × 10 × 0.3 mm	28	95	[72]
	PBS	25	7	100 um thickness	5	154	[75]
	Sodium acetate/sodium hydrogen phosphate buffer	25	4	ND	1.8	154	[74]
	Sodium acetate/sodium hydrogen phosphate buffer	25	12	ND	52	154	
	Compost (ISO 14855-1:2005)	58		Film 20 × 20 mm	98	100	
PBF-PCL	Sodium phosphate buffer	37	7.4	10 mm diameter, 20–30 mg	1.5	40	[86]
	Sodium phosphate buffer/porcine pancreas lipase	37	7.4	10 mm diameter, 20–30 mg	40	40	
	PBS	37	7.4	2 × 2 × 0.03 cm	10	56	[85]
	PBS/porcine pancreas lipase	37	7.4	2 × 2 × 0.03 cm	20	56	
	PBS/*Candida Antarctica* lipase B	37	7.4	2 × 2 × 0.03 cm	32	56	
PPeF-PCL	PBS/P cepacia, R oryzae	37	7.2	5 × 5 cm × 0.4 mm	25	15	[95]
PHeF-PCL	PBS/P cepacia, R oryzae	37	7.2	5 × 5 cm × 0.4 mm	25	32	
PBF-PC	PBS/porcine pancreas lipase	37	7.4	2 × 2 × 0.5 mm	14.5	28	[84]
PBF-PIsF/PIC/PBSu	PBS	37	7.4	20 × 10mm, 20–30 mg	2.5	28	[70]
	PBS/porcine pancreas lipase	37	7.4	20 × 10mm, 20–30 mg	12.5	28	
PBF-PImF	Sodium phosphate buffer	37	7.4	10 mm diameter,20−30 mg weight disks	20	30	[66]
	Sodium phosphate buffer/porcine pancreas lipase	37	7.4	10 mm diameter,20−30 mg weight disks	50	30	
PDF-PIsF	Garden soil	ND	6.5	20 × 10 × 0.1 mm	15	161	[100]
